# Tomato-made edible COVID-19 vaccine TOMAVAC induces neutralizing IgGs in the blood sera of mice and humans

**DOI:** 10.3389/fnut.2023.1275307

**Published:** 2024-01-08

**Authors:** Zabardast T. Buriev, Shukhrat E. Shermatov, Dilshod E. Usmanov, Mukhammadjon K. Mirzakhmedov, Khurshida A. Ubaydullaeva, Venera S. Kamburova, Bakhtiyor K. Rakhmanov, Mirzakamol S. Ayubov, Adkham N. Abdullaev, Jakhongir B. Eshmurzaev, Behzod O. Mamajonov, Akmal A. Tulanov, Adolat A. Ismailova, Tatyana A. Petrova, Ramazan J. Rozumbetov, Tamara U. Aripova, Muzaffar I. Muminov, Khusnora Y. Ermatova, Dilbar A. Dalimova, Shahlo U. Turdikulova, Abdusattor Abdukarimov, Ibrokhim Y. Abdurakhmonov

**Affiliations:** ^1^Center of Genomics and Bioinformatics, Academy of Sciences of Uzbekistan, Tashkent, Uzbekistan; ^2^Institute of Immunology and Human Genomics, Academy of Sciences of Uzbekistan, Tashkent, Uzbekistan; ^3^Center of Advanced Technologies, Ministry of Higher Education, Science and Innovations of Uzbekistan, Tashkent, Uzbekistan

**Keywords:** SARS-CoV-2, Edible vaccine, RBD-specific neutralizing antibodies, viral neutralizing activity, efficiency, safety

## Abstract

Plant-based edible vaccines that provide two-layered protection against severe acute respiratory syndrome coronavirus 2 (SARS-CoV-2) outweigh the currently used parenteral types of vaccines, which predominantly cause a systemic immune response. Here, we engineered and selected a transgenic tomato genotype (TOMAVAC) that stably synthesized an antigenic S1 protein of SARS-CoV-2. Two-course spaced force-feeding of mice with ≈5.4 μg/ml TOMAVAC increased up to 16-fold the synthesis of RBD-specific NAbs in blood serum and the significant induction of S-IgA in intestinal lavage fluid. In a surrogate virus neutralization test, TOMAVAC-induced NAbs had 15–25% viral neutralizing activity. The results suggested early evidence of the immunogenicity and protectivity of TOMAVAC against the coronavirus disease 2019 (COVID-19) infection. Furthermore, we observed a positive trend of statistically significant 1.2-fold (average of +42.28 BAU/ml) weekly increase in NAbs in the volunteers' serum relative to the initial day. No severe side effects were observed, preliminarily supporting the safety of TOMAVAC. With the completion of future large-scale studies, higher-generation TOMAVAC should be a cost-effective, ecologically friendly, and widely applicable novel-generation COVID-19 vaccine, providing two-layered protection against SARS-CoV-2.

## Introduction

Vaccination is considered the most effective way to treat and prevent severe acute respiratory syndrome coronavirus 2 (SARS-CoV-2), which is currently infecting more than 650 million people around the globe (1–5). Presently, most commercial vaccines are parenteral and administered intramuscularly, inducing only systemic immunity (6–9). However, for air droplet-spreading SARS-CoV-2, parenteral vaccines are less effective for the protection of viral transmission (10, 11), enabling its successful replication in the epithelial cells of the upper respiratory tract. Because of ineffective primary-entrance cell viral neutralization by circulating IgG (NAbs), there is a risk of developing and spreading new SARS-CoV-2 variants of concern (VOCs) (10). The production of parenteral vaccines is resource-intensive, ecologically unfriendly, and requires special conditions for transportation, storage, and use (5, 8, 12–14).

Alternatively, mucosal vaccines, including plant-based edible types, activate both mucosal and systemic immune responses, providing adequate and long-term protection against viral transmission, disease progression, and antiviral prophylaxis (10, 11). They also offer an opportunity for targeted folding and post-translational modifications of antigenic proteins with the desired properties (5, 15). Several attempts have been made toward developing plant-based COVID-19 vaccines (5, 16–20), and many plant-carrier-based examples exist for various infectious diseases (8, 9, 12–14). Here, we present the results of our efforts in developing transgenic tomato plants expressing the RBD subunit of the S1 protein of SARS-CoV-2 with early evidence for the immunogenicity, neutralizing activity, and safety of the first tomato-based edible vaccine (TOMAVAC) against COVID-19.

## Methods

### TOMAVAC plant development

**Figure 1:**
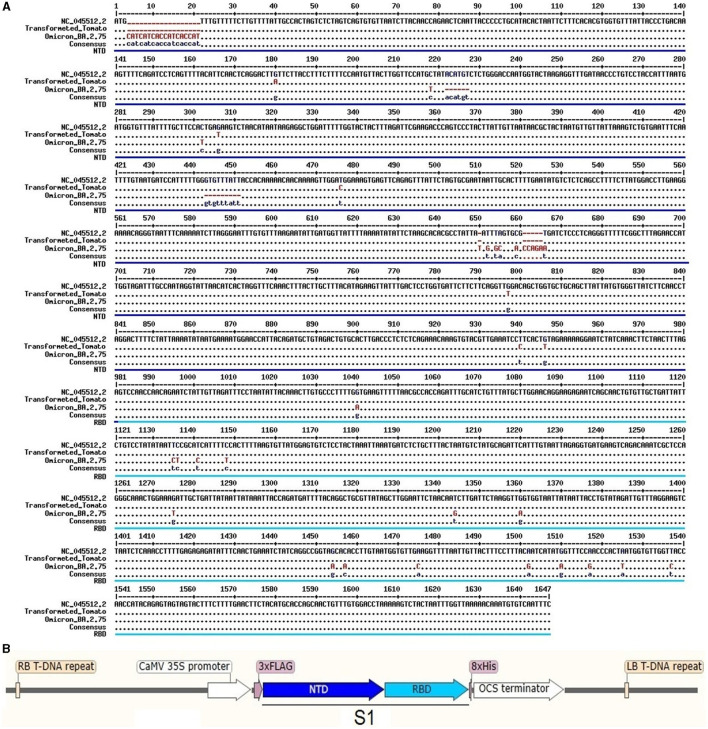
The S1 gene sequence of SARS-CoV-2 was used to construct a binary vector: **(A)** sequence alignment and **(B)** a scheme of vector construction used for tomato transformation.

A binary pART27 vector bearing the custom synthesized *S1* gene (Figure 1) of SARS-CoV-2, driven by the CaMV 35S viral promoter, was constructed and transformed into *A. tumefaciens* strain LB4404 (21). The *in fruit* transformation of tomatoes (*Solanum lycopersicum* cv. Bella-Rossa) was carried out by modified methods (22, 23). The success of the transformation into *A. tumefaciens* strain LB4404 and tomato genomes were confirmed and monitored by PCR amplification of the S1 gene from the genomic DNA of the transformants. Transgenic plant development and characterization, genomic DNA isolation, PCR, and qPCR analyses, including relative expression and qPCR-based transgene copy number identification, were carried out as described in our previous work (24), optimizing for tomatoes. S1-specific protein synthesis in transgenic plants was determined using western blotting and ELISA (Supplementary Tables 1–6). The use of plant material and the performance of experimental research on such plants in the study comply with guidelines for conducting genetic engineering research in the Center of Genomics and Bioinformatics, Academy of Sciences of Uzbekistan. The detailed step-by-step protocols can be found in the Supplementary material section of this article.

### TOMAVAC feeding in mice

In total, 100 (*n* = 100) 20-day-old outbred ICR CD-1 mice were kept in plastic cages and fed between 7–8 a.m. and 4–5 p.m. daily on a standard diet (Supplementary Methods) adaptation. After NAbs analysis of serum and initial verification of the noninfected status of the mice (sacrificing *n* = 4), 96 healthy mice (*n* = 96) were divided into three equal groups (*n* = 24 per group, male and female). Groups were (1) the TOMAVAC group, force-fed with 1 ml of homogenate (equivalent to ≈0.77 μg S1 protein) of the transformation event 4; (2) the untransformed tomato group, force-fed with 1 ml of homogenate of the original cv. Bella Rossa^®^ (3) a control group fed only with a standard diet; and (4) a positive control group parenterally vaccinated with AstraZeneca (ChAdOx1 NCoV-19; Cambridge, United Kingdom). A 7-day-spaced, two-dose force-feeding with tomatoes was performed using a syringe before every morning feeding (**Figure 3A**). A two-dose (day 0 and day 14), 7-day-spaced intramuscular AstraZeneca (75 μl of working solution) immunization was carried out in mice in compliance with asepsis rules. Each dose contained ≈16 × 10^6^ infectious units in a 75 μl of injection solution. To do this, 10 μl of the original AstraZeneca vaccine was diluted to 1,000 μl by adding sterile standard saline solution. Then, the obtained solution was diluted to a final concentration of 16 × 10^6^ infectious units by sterile solution.

For the analyses of NAbs, blood samples and intestinal lavage fluid were taken on day 14 (*n* = 24, six mice per group), day 28 (*n* = 24, six mice per group), day 42 (*n* = 24, six mice per group), and day 56 (*n* = 16, four mice per group). Mouse experiments were conducted with ethics board approval (no. 3/30062022) and in accordance with the US National Institutes of Health Guidelines for the Care and Use of Laboratory Animals (25). Animal experiments were conducted at the Drug Standardization Scientific Center, Pharmaceutical Institute, Tashkent, Uzbekistan (Supplementary Protocol).

### Determination of NAbs titer and neutralizing ability

RBD-specific serum IgG was assessed in three different groups using the SARS-CoV-2 (2019-nCoV) Spike RBD Antibody Titer Assay Kit (Mouse) according to the manufacturer's instructions (Krishgen Biosystems, Cerritos, California, United States). The neutralizing activity of RBD-specific antibodies was determined in blood serum on days 42 and 56 post-vaccination using the SARS-CoV-2 surrogate Virus Neutralization Test (sVNT) kit according to the manufacturer's instructions (GenScript, Piscataway, New Jersey, United States). RBD-specific secretory immunoglobulin A (S-IgA) quantified by ELISA using the Mouse Anti-2019 nCoV(S)IgA ELISA Kit (Wuhan Fine Biotech Co., Ltd., Wuhan, China) according to the manufacturer's instructions (see Supplementary Methods).

### Proof-of-concept human consumption study

All 14 healthy adults aged 30–52 years volunteered to participate in a human proof-of-concept experiment. All experiments were designed and carried out in accordance with the methods of relevant guidelines and regulations. The ethical board of the Center of Genomics and Bioinformatics, Academy of Sciences of Uzbekistan, approved the experimental protocols. Informed consent was obtained from all subjects and/or their legal guardian(s). Following the protocol approval, volunteers were asked to be randomly divided into two groups: (1) those who were invited to take TOMAVAC (*n* = 7) before dining and (2) those who were invited to have their regular food (*n* = 7). Both groups were instructed not to have any other vaccinations during the experiments (Supplementary Protocol). All procedures were explained clearly to all participants, and their approval of using the collected data for “research purposes” was obtained. Participants were informed about blood test results but deidentified for reporting purposes.

Briefly, the experimental group consumed 50 g of TOMAVAC on an empty stomach daily for 3 consecutive days, 20–30 min before dining. Blood samples were taken from all participants before consumption (day 0) and on days 7, 14, and 21. The NAbs levels were tested at the Institute of Immunology and Human Genomics, Uzbekistan, using the automatic chemiluminescence immunoassay analyzer MAGLUMI series, according to the manufacturer's instructions for human sera (Snibe Diagnostic, Pingshan, China).

### Statistical analysis

Expression and ELISA data in tomatoes were analyzed using an ordinary one-way ANOVA with Tukey's multiple comparisons. For mouse data, after checking the normal distribution using the Shapiro-Wilk test or data normalization using the log10 transformation, an ordinary one-way ANOVA was used with the recommended Tukey's multiple comparisons. A nonparametric Kruskal-Wallis (KW) one-way ANOVA was also applied to determine the statistical significance of abnormal data and seek additional statistical evidence to judge ordinary ANOVA findings. In the human study, due to data abnormality, the nonparametric Friedman's ANOVA test, followed by Dunn's multiple comparisons, was performed comparing the initial NAbs mean ranks to the repeated measurement mean ranks of NAbs from days 7, 14, and 21. Data analysis and visualization were performed using GraphPad Prism version 8.0.1 for Windows (www.graphpad.com; GraphPad Software, San Diego, California, United States). A threshold of *p* < 0.05 was considered significant (Supplementary Tables 7–9).

### Ethics of the research

All methods were performed in accordance with the relevant guidelines and regulations reviewed and approved by the Institutional Ethics Board of the Center (Supplementary Protocol). The use of plants in this study complied with relevant institutional, national, and international guidelines and legislation. All animal results are reported following the ARRIVE guidelines.

## Results

### TOMAVAC plant development

A binary vector on the pART27 backbone was successfully constructed, inserting the custom synthesized 1,735-bp long recombinant *S1* gene (1,643 bp; Figure 1A), flanking 75-bp 3xFlag-tag (DYKDDDDK-tag), and 24-bp histidine repeats (8xHis; Figure 1B). The *S1* gene sequence information was derived from the consensus sequence of 18 SARS-CoV-2 genotypes, which covered all six mutations (compared to NC_045512.2: G180A, G306T, T476C, G797T, T940C, and G946T) found in both GR and S clade genomes sequenced from Uzbekistan (21). Furthermore, a suspension culture of *A. tumefaciens* strain LBA4404 bearing *pART27::COVID-19_S1* was obtained with a success rate of 96%.

**Figure 2:**
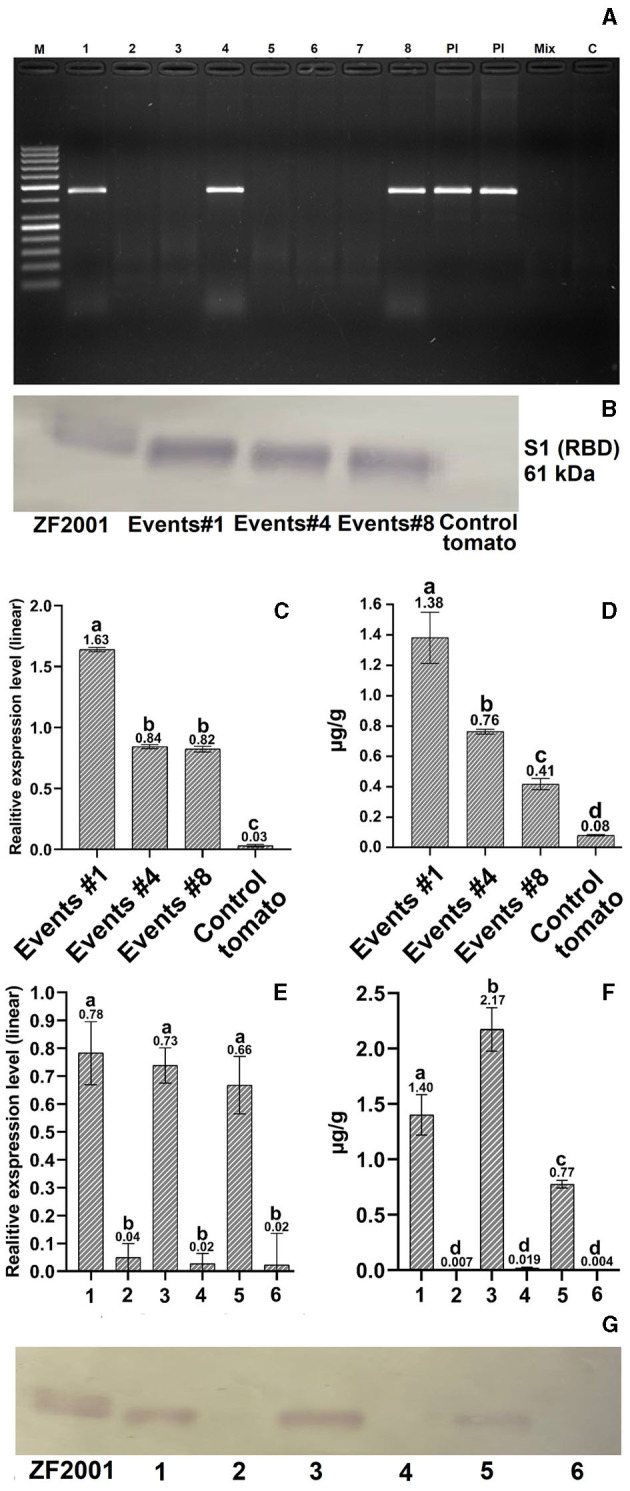
Molecular evaluation of transformed tomatoes. **(A)** PCR analysis: M – GeneRuler 50 bp DNA Ladder (Thermo Fisher, Waltham, MA, United States); 1–8 – transformed tomato plant DNA; Pl – S1 plasmid DNA (a positive control); Mix – Master mix without DNA (a negative control); C – a negative untransformed tomato plant DNA control. **(B, G)** Western blot quantification of expressed protein in transgenic tomatoes. RBD protein (ZF-UZ-VAC200123,28 with 61.7 kDa) and untransformed tomatoes were used as positive and negative controls **(C, E)** Relative expression analysis of transformed T1-generation tomato plants and tissues. The untransformed tomato was used as a negative control. **(D, F)** ELISA analysis of transformed T_1_-generation tomato plants: leaf tissue (1), unripened green tomato (2), ripened red tomato (3), and control tissues from untransformed tomato (2, 4, and 6). Different letters designate significant differences at the *p* ≤ 0.05 level of one-way ANOVA or Kruskal-Wallis one-way nonparametric ANOVA (Supplementary Tables 1, 3), with similar letters reporting nonsignificant differences. Refer to Supplementary Figures S1–S3 for the original gel images for **(A, B, G)**, cropped for presentation purposes.

A total of 46 T_0_ generation PCR-positive tomato seedlings were recovered out of 405 in-fruit agro-infiltrations, revealing 11% of successful transformation events. Three independent T1 generation tomato transformants (Figure 2A; see Supplementary Figure S1 for original gel image), selected for further analyses, showed stable insertion of the S1 gene in their genomes with relative linear expression levels on average of 0.82–1.63 of the S1 gene in tomato seedlings (Figure 2C; Supplementary Table 1). Tomatoes from single copy transformation event 4, expressing ≈0.77 μg/g S1 protein of SARS-CoV-2, were chosen for further immunization experiments (Supplementary Tables 2, 3).

### Immunogenicity and neutralization analyses in mice

**Figure 3:**
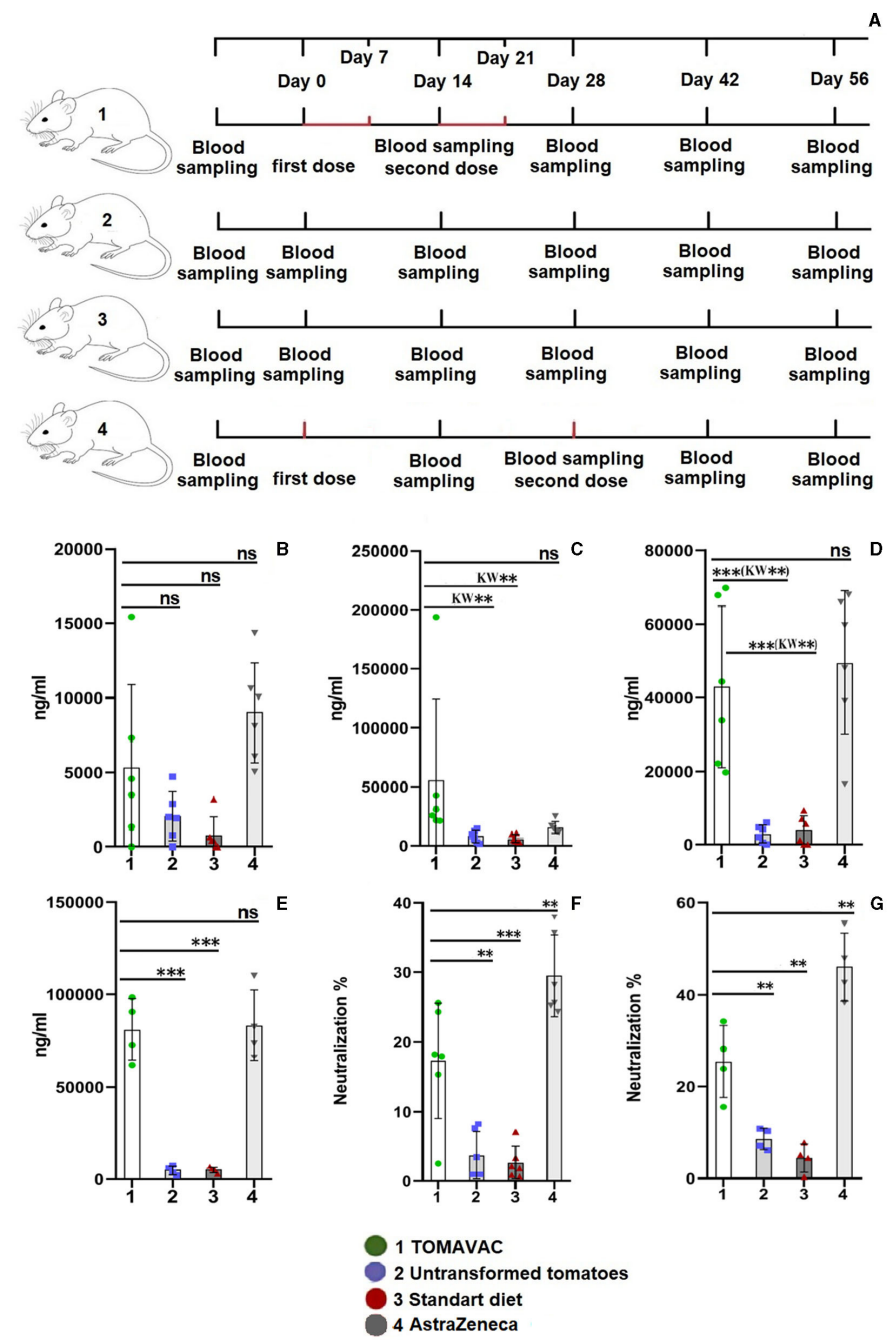
Titer and neutralizing activity of RBD-specific serum IgG in mice after oral vaccination. **(**A) – animal experiment scheme; **(B)** – titer on day 14 postvaccination; **(C)** – titer on day 28 postvaccination; **(D)** – titer on day 42 postvaccination; **(E)** – titer on day 56 postvaccination; **(F)** – the neutralizing activity of NAbs on day 42 postvaccination; and **(G)** – neutralizing activity on day 56 postvaccination. Statistical significance level (**–≤0.01, and ***–≤0.001) from ordinary one-way ANOVA; Kruskal–Wallis (KW) one-way ANOVA, KW*–≤0.05, KW**–≤0.01, KW***–≤0.001, Supplementary Table 7).

We designed a 7-day-spaced, two-dose force-feeding study (Figure 3A). In the TOMAVAC and AstraZeneca (ChAdOx1 NCoV-19; parenteral vaccination control) groups, analysis of the blood serum on day 14 showed insignificant induction of RBD-specific NAbs above 5,300 and 9,000 ng/ml (Figure 3B; Supplementary Table 7) compared to controls. After the second dose of immunization, however, the average titer of RBD-specific NAbs in the TOMAVAC-fed group increased more than 7–9 times (>55,500 ng/ml) in comparison to the controls on day 28 (*p* = 0.01, KW and *p* = 0.001, ANOVA; Supplementary Table 7; Figure 3C).

In the AstraZeneca group, however, the observed NAbs increase (14,669 ng/ml) was insignificant among groups (*p* > 0.2, KW and ANOVA). On day 42, a statistically significant (*p* ≤ 0.01, KW and ANOVA) 11- to 14-fold increase (average >43,000 ng/ml) in the titer of RBD-specific NAbs was revealed in the TOMAVAC group (Figure 3D, Supplementary Table 7) and AstraZeneca (average >49,500 ng/ml; *p* ≤ 0.02, KW and ANOVA) groups compared to non-vaccinated controls; however, the NAbs increase between the TOMAVAC and AstraZeneca groups was insignificant (*p* > 0.8, KW and ANOVA). Interestingly, relative to day 28, an average 24% decrease in NAbs level was observed in the TOMAVAC group. In contrast, on day 56, the titer of RBD-specific NAbs increased from 15- to 17-fold in the TOMAVAC (average >80,850; *p* ≤ 0.0001, ANOVA) and AstraZeneca (average >83,000; *p* ≤ 0.0001, ANOVA) groups compared to controls (Figure 3E; Supplementary Table 7).

The analysis of the neutralizing activity of RBD-specific NAbs showed that in the TOMAVAC group, the average neutralization activity was 15% (*p* ≤ 0.01, ANOVA or *p* ≤ 0.05, KW; Figure 3F) and 25% (*p* ≤ 0.01; ANOVA; Figure 3G: Supplementary Table 7) on days 42 and 56, respectively. However, the neutralizing activity of RBD-specific NAbs in the AstraZeneca group was significantly higher (29%−46% on the same periods; *p* ≤ 0.01; ANOVA; Figure 3G, Supplementary Table 7) than what was observed in the TOMAVAC group. In the control groups, the level of neutralization was within 5%−7% and was statistically insignificant (Figures 3F, G, Supplementary Table 7). No severe side effects were recorded in mice during or after the consumption of TOMAVAC. Two 90-day-old mice per group, including controls, died at week 8 in the mouse experiment. The cause of death is estimated to be natural, usually occurring with long-period feeding experiments (L.G. Gafurova, Tashkent Pharmaceutical Institute, personal communication).

**Figure 4:**
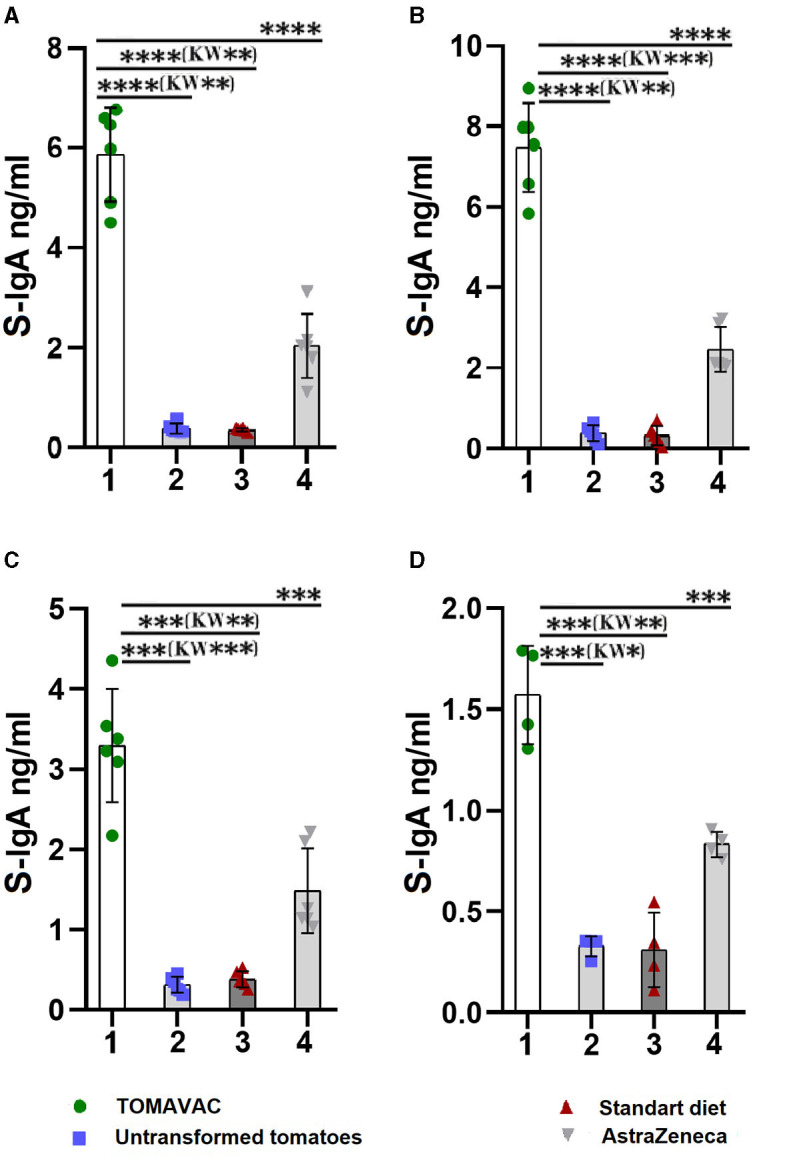
Titer of RBD-specific mucosal S-IgA in mice after oral vaccination. **(A)** – titer on day 14 post-vaccination; **(B)** – titer on day 28 post-vaccination; **(C)** – titer on day 42 post-vaccination; **(D)** – titer on day 56 post-vaccination. Statistical significance level (*–≤0.05, **–≤0.01, ***–≤0.001 and ****–≤0.0001) from ordinary one-way ANOVA; Kruskal-Wallis (KW) one-way ANOVA, KW*–≤0.05, KW**–≤0.01, KW***–≤0.001, Supplementary Table 8).

The analysis of the titer of RBD-specific S-IgA in the TOMAVAC group on day 14 showed a significant induction of 5.9 ng/ml (Figure 4A, Supplementary Table 8) compared to controls. We also detected the induction of RBD-specific S-IgA in the AstraZeneca group, but its level was almost three times lower than that of the TOMAVAC group. After the second dose of vaccination, we revealed a further elevation in S-IgA levels in the TOMAVAC (average 7.5; *p* < 0.0001, ANOVA) and AstraZeneca (average 3.5; *p* < 0.0001, ANOVA) groups compared to controls on day 28 (Supplementary Table 8; Figure 4B). On days 42 and 56, we found a two- to fourfold decrease in RBD-specific S-IgA levels in the TOMAVAC and AstraZeneca groups relative to day 28 (Figures 4C, D).

### Concept-of-proof human consumption analysis

In all volunteers consuming TOMAVAC (50 g or ≈38.5 μg S1 protein daily during 3 days), a significant (*p* ≤ 0.01, Dunn's multiple comparisons adjusted) positive trend of weekly increase (with an average of +42.28 BAU/ml/3 weeks) of NAbs was observed relative to the initial day levels (Supplementary Figures S4A, C). The increase in serum NAbs started on day 7 with an average of +35.39 BAU/ml (*p* ≤ 0.01, Dunn's multiple comparisons adjusted; Supplementary Table 9). NAbs increased on day 14 with an average of +37.73 BAU/ml (*p* = 0.06 in Dunn's multiple comparisons). Furthermore, the statistically significant average +53.44 BAU/ml NAbs increase was detected on day 21 (*p* = 0.01, Dunn's multiple comparisons adjusted; Supplementary Figures S4A, C).

In one participant, a +8.15 BAU/ml increase in NAbs level was determined on day 7; however, on days 14 and 21, a decrease (−11.85 and −8.14 BAU/ml, respectively; Supplementary Table 10) of NAbs was observed relative to the initial level. In another participant, although a positive increase relative to the initial day was recorded on days 7–21, an increased level of NAbs (+13.0 BAU/ml) on day 7 decreased (−8.9 and −5.2 BAU/ml) on days 14 and 21. In contrast, in the control group, a gradual negative trend of weekly decrease (average −28.7 BAU/ml; *p* ≤ 0.001, Freidman's ANOVA) was observed in the level of NAbs relative to the initial day (Supplementary Figures S4B, C; Supplementary Table 9). No severe side effects were recorded in volunteers during or after the consumption of TOMAVAC. The observed feeling of abdominal pressure on the consumption days was resolved after immunization (Supplementary Table 10).

## Discussion

The plant-made COVID-19 vaccine development efforts were recently well-reviewed by Su et al. (20). Many such parenteral candidate vaccines are in their early stages of application, with few commercialized for research and diagnostic purposes (20). In this study, we report the first edible COVID-19 tomato vaccine, TOMAVAC, derived by constitutively overexpressing the consensus sequence of the S1 protein of SARS-CoV-2, covering the sequence variation of SARS-CoV-2 spread in Uzbekistan (26). In contrast, recently, the SARS-CoV-2-S1-Fc fusion protein (16–18) or a SARS-CoV-2 perfusion spice trimer protein (19) was transiently expressed in tobacco and harvested for parenteral vaccination studies in animals.

In our study, among several stable transformation events, we selected the single-copy transgenic genotype with no antibiotic resistance marker gene (event 4), which reduces the chances of gene silencing and instability in the next generations and is the key to the biosafety and purity of the transgenic plant product (27). While our study successfully demonstrated the expression of the S1-specific protein in transgenic tomato plants, we did not observe any discernible phenotypic differences between the transgenic and non-transgenic tomato plants regarding growth, development, or overall appearance. Both sets of plants appeared phenotypically similar and exhibited normal growth patterns, suggesting that the synthesis of the S1 protein did not visibly affect the external morphology or growth characteristics of the transgenic plants compared to the non-transgenic ones. The fruits of transgenic and non-transgenic plants did not differ in appearance and taste.

TOMAVAC expressing ≈0.77 μg/g S1 protein of SARS-CoV-2 provided an opportunity to design and conduct scalable oral immunization experiments. One explanation for the almost threefold reduced amount of S1 protein in ripened tomatoes compared to the unripened green tomatoes (*p* < 0.0001, ANOVA) could be the influence of the fruit-maturation process. This requires further study and will be crucial for TOMAVAC dose standardization.

The receptor-binding domain (RBD) of the S1 protein was immunogenic and non-allergenic, with very subtle side effects when used as a parenteral protein subunit recombinant vaccine in a previous study (28). Recent studies (16–18) reported that the plant-produced S1 subunit protein vaccines against SARS-CoV-2 were immunogenic without any safety concerns in animal experiments. Previous studies also showed that three injections of 10 μg/dose of RBD-specific antigen of S1 protein adequately induced systemic immunity in mice (29). It was reported that two-dose parenteral vaccination with purified 10 μg and 25–50 μg of tobacco-produced SARS-CoV-2 RBD-Fc fusion protein caused high levels of NAbs in mice and monkeys, respectively (16–18). However, with edible plant-based vaccines, a higher dose of expressed antigen may be needed than that used for parenteral immunizations (13, 14).

Pogrebnyak et al. (30) reported that feeding with 500 mg of lyophilized transgenic tomato fruit (or 50 mg dry roots of transgenic tobacco) expressing 79 kDa SARS-CoV S protein increased fecal IgA levels in mice compared to controls. No increase in serum IgG was found in oral vaccination, although the systemic immune response was caused by a booster dose of S protein injection (30). In contrast, in our study, two courses of spaced weekly feeding of mice with a total of ≈7 g ripened TOMAVAC (≈5.4 μg antigen) bearing intact 61 kDa S1 protein were sufficient for obtaining a primary immune response, which was significantly boosted after the second dose feeding. Contrasting results compared to Pogrebnyak et al. (30) could be due to differences in source, size, sequence, quantity, and oral force-feeding protocol used in our study.

The difference in the increase of NAbs between the TOMAVAC and AstraZeneca groups was insignificant (*p* > 0.1; ANOVA), demonstrating a similar level of induction of systemic immunity via oral vaccination in our study. However, it is noteworthy to mention that due to known evidence of absorptional losses of the oral vaccines in the harsh gastrointestinal environment (13, 14) and to achieve a better NAbs induction correlation between parenteral and oral immunizations in mice, we used over a sixfold diluted dose (16 × 10^6^ infectious units) for AstraZeneca vaccine injection in our study compared to what was used (1 × 10^8^ infectious units) by Silva-Cayetano (31).

The cause of the 24% observed decrease in NAbs level on day 42 (relative to day 28) in the TOMAVAC group is unknown. However, it could be due to calibration differences between sample measurements, as we observed similar higher NAb indices (>5.8) for both negative control groups on day 28 than on day 42 (< 3.9). We also found nonsignificant differences in NAbs levels in the TOMAVAC group after data normalization (4.6 on day 28 vs. 4.61 on day 42; Supplementary Table S7).

The neutralization activity of the induced NAbs is pivotal against viral infection (32). Margolin et al. (19) reported that immunization with tobacco-produced SARS-CoV-2 perfusion spike trimer protein was less protective than mammalian cell culture-derived protein in their animal (hamster) model. Our *in vitro* neutralization experiment, based on antibody-mediated blockage of the interaction between the angiotensin-converting enzyme 2 (ACE2) receptor protein and the receptor-binding domain (32), preliminarily suggested an insufficient level of protective response of TOMAVAC against SARS-CoV-2, where we have observed higher neutralization (30%−46%) in parenteral vaccination control with AstraZeneca. This contrasting evidence requires additional studies with TOMAVAC oral vaccination in larger samples and longer durations, which is in progress. There is also a need for an *in vivo* assay of vaccinated animal models and live viruses for a better estimate of vaccine-induced protection against SARS-CoV2 infection.

Mucosal immunity significantly contributes to the defense against viral pathogens by providing a frontline barrier and an effective immune response. Immunization via oral vaccine administration, incorporating specific antigens, stimulates the synthesis of secretory immunoglobulin A (IgA) on mucosal surfaces (33, 34). Studies showed that oral immunization of mice with lactic acid bacteria expressing the SARS-CoV-2 spike (S) protein receptor-binding domain (RBD) S1 subunit increased the fecal IgA levels 1.4-fold higher in the immunized mice than controls after two boosting doses on day 22 (33). In our study, the level of S-IgA in intestinal lavage fluid after two doses of vaccination was 23-fold higher than the standard diet control group on day 28 (*p* < 0.0001; ANOVA). The high S-IgA levels in our study can be explained by higher concentrations of S-IgA within the intestinal mucosa than in feces.

Analysis of the antibody titer on day 56 revealed a decrease in the level of S-IgA in the TOMAVAC group by 3.75-fold compared to day 14 and in the AstraZeneca group by 2.4-fold. However, an increase in the level of S-IgA on days 14 and 28 may indicate the effectiveness of TOMAVAC against the penetration of viruses through the mucous membrane. The tandem-repeat dimeric RBD-based recombinant vaccine was revealed to be immunogenic, protective, and safe in 3-injection (25 μg/dose) clinical trials in humans (27, 35). There is, however, no evidence of edible amounts of S1 antigen that remain intact and adequate for induction of the immune response in the gastrointestinal tract [with 80% acidic and 20% enzymatic digestions (13)] of humans. Previous studies on potatoes reported that three doses of 100 g, containing a total of 1 mg of hepatitis virus B antigen (HBsAg), were partially effective. In contrast, a parenteral vaccination with 40 μg/dose HBsAg caused a sufficient immune response (14). However, there is a caution about administering large quantities of oral vaccine, presumably causing immune tolerance if the consumed antigen molecule does not give sufficient pathogenic signals (13, 14). Here, a 3-day consumption of TOMAVAC, containing ≈38.5 μg [total ≈116 μg vs. 75 μg parenterally used for human vaccination (28)] plant-expressed S1 antigen, was adequate to induce some NAbs in serum with a positive weekly increasing trend in volunteers vs. a negative weekly decreasing trend in controls (Supplementary Figure S4C). In two volunteers, we observed a decrease in day 7-induced NAbs on days 14 and 21, which remains unclear. This may be due to the personal immune system of volunteers, which requires further study, e.g., parenteral immunization responses in these individuals (36). It also remains unknown whether TOMAVAC protects against currently spread Delta/Omicron variants. However, the results justify future efforts to replace the plant-expressed S1 protein region with emerging VOCs, which are in progress in our laboratory.

### Limitations of the study

There are several limitations and demands for future studies, including (a) selection and seed increase of the next generation (T_1:5_) stable self-pollinated genotypes with efficient antigen synthesis in tomatoes (8, 12), (b) evaluation of the stability and duration of storage/shelf life of synthesized S1 protein in mature tomato fruits (8, 14), and (c) study of the influence of gastrointestinal tract condition (13) on tomato-delivered S1 protein to quantify and scale an optimal dosage amount [standardizing of an antigen (14)] for safe consumption of TOMAVAC, inducing efficient immunizations and sufficient protection (19) but not generating immune tolerance (12, 13).

Future studies are needed to investigate possible alterations in biochemical parameters, nutritional composition (such as differences in nutritional content, taste, texture, or other quality aspects), or physiological traits between transgenic and non-transgenic plants to better understand any potential impacts of S1 protein synthesis on plant performance. Future research is also needed, including longitudinal studies with extended follow-up periods, which are essential to comprehensively evaluating the post-vaccination stage effects, the durability of immune responses, and the vaccine's ability to offer sustained protection against COVID-19.

There is an opportunity to develop lyophilized (8, 14) TOMAVAC-based capsules (5) with standardized edible doses for safe delivery and enhanced immunogenicity (14), which demand future investigations with the availability of higher-generation stable transgenic tomato plants. These will help to produce sufficient standardized TOMAVAC for conducting large-scale human consumption clinical trials justifying (a) the efficiency of protection against current and emerging COVID-19 infections and (b) public perception and acceptance (12, 14), as well as proper regulatory measures (20), which require future investigations and investments.

## Conclusion

Early evidence from small-scale proof-of-concept study results promises an opportunity for performing inexpensive, eco-friendly, and safe plant-based edible vaccine immunization programs against COVID-19. This might offer longer-term, two-layered protection that limits viral transmission and disease progression, helping to secure global health.

## Data availability statement

The raw data supporting the conclusions of this article will be made available by the authors, without undue reservation.

## Ethics statement

The studies involving humans were approved by the Institutional Ethics Board of the Center of Genomics and Bioinformatics, Uzbekistan. The studies were conducted in accordance with the local legislation and institutional requirements. The participants provided their written informed consent to participate in this study. The animal study was approved by Institutional Ethics Board of the Center of Genomics and Bioinformatics, Uzbekistan.

## Author contributions

ZB: Conceptualization, Funding acquisition, Investigation, Methodology, Project administration, Supervision, Writing – review & editing. SS: Conceptualization, Investigation, Methodology, Validation, Visualization, Writing – review & editing. DU: Data curation, Investigation, Visualization, Writing – review & editing. MM: Investigation, Methodology, Visualization, Writing – review & editing. KU: Investigation, Methodology, Resources, Writing – original draft, Writing – review & editing. VK: Conceptualization, Investigation, Methodology, Visualization, Writing – review & editing. BR: Investigation, Resources, Writing – review & editing. MA: Data curation, Investigation, Methodology, Visualization, Writing – review & editing. ANA: Methodology, Resources, Visualization, Writing – review & editing. JE: Investigation, Resources, Visualization, Writing – review & editing. BM: Data curation, Investigation, Writing – review & editing. AT: Investigation, Project administration, Resources, Writing – review & editing. AI: Investigation, Validation, Writing – review & editing. TP: Investigation, Writing – review & editing. RR: Methodology, Writing – review & editing. TA: Investigation, Supervision, Writing – review & editing. MM: Investigation, Methodology, Validation, Writing – review & editing. KE: Methodology, Resources, Writing – review & editing. DD: Methodology, Project administration, Writing – review & editing. ST: Project administration, Supervision, Writing – review & editing. AA: Funding acquisition, Supervision, Writing – review & editing. IA: Conceptualization, Project administration, Supervision, Validation, Visualization, Writing – original draft, Writing – review & editing.
