# Supplementary material for: Tomato-made edible COVID-19 vaccine TOMAVAC induces neutralizing IgGs in the blood sera of mice and humans
Source: Front Nutr. 2024 Jan 8;10:1275307. doi: 10.3389/fnut.2023.1275307 (PMC10800535; doi:10.3389/fnut.2023.1275307)
Supplement: Supplementary file 1 [file Data_Sheet_1.docx]

Supplementary Material

Supplement to: Buriev ZT, Shermatov SE, Usmonov DE, et al. Tomato-made edible COVID-19 vaccine TOMAVAC induces neutralizing IgGs blood serums of mice and humans, submitted.

**Supplementary methods**

**Development of the kanamycin resistance gene-free pART27 vector**

The binary vector pART27 is widely used for the genetic transformation of plants and is commercially available through many companies. The length of the vector is 11667 bp. Our genetic cassette was cloned between Xho I and Xba I restriction sites. The synthetic cassette is driven by CaMV 35S viral promoter ended by OCS terminator sites (Fig. 1). The pART27 carries the RK2 minimal replicon for maintenance in Agrobacterium, the ColE1 origin of replication for high-copy maintenance in *Escherichia coli* and the Tn7 spectinomycin/streptomycin resistance gene as a bacterial selectable marker. The T-DNA carries the kanamycin resistance gene (nopaline synthase promoter-neomycin phosphotransferase-nopaline synthase terminator) distal to the right border relative to the lacZ' region.

The following detailed protocol was used to develop a kanamycin gene-free pART27 vector. Two pairs of primers were designed to amplify the upstream and downstream regions of the NTPII gene of the pART27 vector. The forward sequence of the first primer with the site of the AgeI restriction enzyme in the 5’-end was designed for the OCS terminator. The reverse sequence with the site of AflII restriction enzyme in the 5’ -end was designed for the NOS promoter. The forward sequence of the second primer with the site of the AflII restriction enzyme in the 5’ -end was designed for the NOS terminator, and the reverse sequence with the site of the BspEI restriction enzyme in the 5’ end was designed for IS1. We amplified from pART27 vector two amplicons with lengths 794 bp and 1428 bp using Phusion™ High-Fidelity DNA Polymerase (Thermo Fisher Scientific). The amplicons were verified by 1.5% agarose gel electrophoresis, and both sequences were cut out from agarose gel. According to the manufacturer’s instructions, the DNA fragments from agarose gel slices were purified using PureLink Quick Gel Extraction Kit (Thermo Fisher Scientific).

Two purified sequences were combined into one sequence by ligation reaction. The ligation reaction was conducted in the total volume of 20 µl with 2 µl 10x ligase buffer (Thermo Fisher Scientific), 1 µl ligase (Thermo Fisher Scientific), 5 µl 794 bp and 1428 bp amplicons. The reaction mix was incubated in a PCR machine at 16°C for 16 hours and at 70°C for 20 minutes and was verified by 1.5% agarose gel electrophoresis. At this stage, we obtained a 2222 bp ligation product.

The restriction reaction was conducted for the pART27 vector in a total volume of 10 µl, with 2 µl Tango Buffer (Thermo Fisher Scientific), 1 µl AgeI/BspEI restriction enzymes (New England Biolabs, USA), and 7 µl pART27 vector. The reaction mix was incubated at 25 °C for 3 hours and was verified by 1.5% agarose gel electrophoresis. The 9784 bp band from the pART27 reaction mix was cut out from agarose gel. The DNA fragment from the agarose gel slice was purified using PureLink Quick Gel Extraction Kit (Thermo Fisher Scientific) according to the manufacturer’s instructions. At this stage, we obtained a linear pART27 vector that does not contain the NTPII gene.

Two ligate the linear pART27 and 2222 bp DNA fragment, the ligation reaction was conducted in the total volume of 20 µl with 2 µl 10x ligase buffer (Thermo Fisher Scientific), 1 µl ligase (Thermo Fisher Scientific), 5 µl 2222 bp DNA fragment, and 12 µl linear pART27 vector. The reaction mix was incubated in a PCR machine at 16°C for 16 hours and at 70°C for 20 minutes and was verified by 1.5% agarose gel electrophoresis. The DNA fragment (kanamycin resistance gene free pART27 vector) from the agarose gel slice was purified using PureLink Quick Gel Extraction Kit (Thermo Fisher Scientific) according to the manufacturer’s instructions. This kanamycin resistance gene-free pART27 vector was used to develop the TOMAVAC gene construct as described below.

**Development of TOMAVAC genetic construct**

The 1735 bp long recombinant S1 gene (Fig. 1a) of SARS-CoV-2 was designed and purchased from GenScript (Piscataway, New Jersey, USA) within the vector Bac_pac_8_3xFLAG_pBac_pac8. Two separate restriction reactions were conducted for Bac_pac_8_3xFLAG_pBac_pac8 and pART27 vectors in a total volume of 10 µl, with 2 µl Tango Buffer (Thermo Fisher Scientific), 1 µl XhoI/XbaI restriction enzymes (Thermo Fisher Scientific) and 7 µl vectors. The reaction mix was incubated at 25 °C for 3 hours and was verified by 1.5% agarose gel electrophoresis. The 1735 bp long recombinant S1 gene band from Bac_pac_8_3xFLAG_pBac_pac8 reaction mix and an 11662 bp band from pART27 reaction mix were cut out from agarose gel. According to the manufacturer’s instructions, the DNA fragments from agarose gel slices were purified using PureLink Quick Gel Extraction Kit (Thermo Fisher Scientific).

The concentration of extracted DNA fragments was measured using a spectrophotometer and diluted to the required concentration. Before the S1 gene cassette was inserted, the kanamycin resistance marker gene sequence within the left and right border region was excised from pART27, as described above. Then, a ligation reaction inserted the recombinant S1 gene fragment into the pART27 vector. The ligation reaction was conducted in the total volume of 20 µl with 2 µl 10x ligase buffer (Thermo Fisher Scientific), 1 µl ligase (Thermo Fisher Scientific), 5 µl S1 gene, and 12 µl pART27 vector. The reaction mix was incubated in a PCR machine at 16°C for 16 hours and at 70°C for 20 minutes. The resulting pART27::COVID-19_S1 plasmid was transformed into a competent Top10 E. coli cell (Invitrogen, Waltham, MA USA) and grown in the liquid media according to the manufacturer's instructions. Plasmid DNAs were isolated and purified using Plasmid DNA Miniprep kits (Thermo Fisher, Waltham, MA, USA).

**Transformation of *A. tumefaciens***

Transformation of *A. tumefaciens* strain LB4404 was performed according to the modified method of Holsters *et al.* (1978) (21). The selection of transformed bacteria was carried out on a selective agar medium YEP (bacto-peptone – 10 g/l, sodium chloride – 5 g/l, yeast extract – 10 g/l, bacto-agar – 15 g/l, pH 7.2), containing rifampicin (20 mg/l) and spectinomycin (50 mg/l). All these reagents were purchased from Phytotechnology Laboratories, Lenexa, Kansas, USA. The success of the transformation into *A. tumefaciens* strain LB4404 was monitored by PCR amplification of the S1 gene from the genomic DNA of the transformed cells.

**Obtaining culture of the transformed *A. tumefaciens***

The culture of the transformed *A. tumefaciens,* confirmed by PCR, was obtained according to the modified method of Yasmeen *et al.* (2009) (22). To do this, the cells that have passed the selection are cultivated in a YEV liquid medium (meat extract – 5 g/l, yeast extract – 1 g/l, peptone – 5 g/l, sucrose – 5 g/l, MgSO_4_ – 493 mg/l, pH 7,2) rifampicin (20 mg/l) and spectinomycin (50 mg/l). The optical density of the resulting suspension was at least 0.6. All these reagents were purchased from Phytotechnology Laboratories, Lenexa, Kansas, USA.

**Tomato transformation**

*In fruit* transformation of tomatoes (*Solanum lycopersicum* cv. Bella-Rossa) was carried out according to the modified methods of Yasmeen *et al.* (2009) (22) and Orzaez *et al.* (2006) (23). Fruits were selected for transformation at an early stage of maturity. The fruits were transformed with a suspension of *A. tumefaciens* with an optical density of the suspension of at least 0.6. All tomatoes were injected twice for two consecutive days. Ten fruits were used for each treatment, and the experiments were carried out in triplicate. Fruits injected with saline were used as a negative control. After the injection, the tomato fruits were kept on a thermostat for 72 hours at 27–28° C. After 72 hours, the seeds were isolated from the transformed tomato fruits. Washed and dried seeds were kept at 4° C for seven days. Seeds that had passed the dormant period were soaked in water (14–15° C) for 24 hours, brought to room temperature 15–18° C, then planted in particular soil and germinated after four days. Using S1 gene-specific PCR, transformed tomato seedlings were selected. Next, the seedlings were grown under controlled phytotron conditions under the following conditions: 22° C for 16 hours during the day and 15° C for 8 hours at night at a relative humidity of 55–65%.

**Isolation of genomic DNA**

Tomato seedling genomic DNA was isolated using the DNeasy Plant Mini Kit (QIAGEN, Hilden, Germany) according to the manufacturer's instructions. Genomic DNA extracted from plant tissues was analyzed by electrophoresis in 0.9% agarose gel. The DNA concentration was assessed against the ratio of lambda phage DNA digested with Hind III restriction enzyme as a control (molecular weight marker).

**PCR analysis**

The reaction mixture for PCR in a volume of 50 µl was prepared according to the following protocol: 1 µl 50 ng/ml genomic DNA, 2.5 µl 10x PCR buffer, 2 µl MgCl_2_, 1 µl 10% PVP, 0.5 µl 10 mM dNTP mixture, 2.5 µl 10 ng/ml correct and reverse primers, 0.5 u. Taq polymerase, H_2_O up to 50 µl. PCR was performed on a ProFlex 96 Sample Block PCR/Thermal Cycler (Applied Biosystems, Waltham, MA USA) under the following conditions: 1 cycle, 94° C, 3 min; 45 cycles - 94° C, 30 s; 54° C, 30 s; 72° C, 45 s; 1 cycle - 72° C, 7 min. For PCR, primers were used, the sequence of which is given in Supplementary Table 4. The gel image of PCR products was photo-documented (Supplemental Fig. S1).

**Copy-number identification**

We followed the real-time qPCR (qRT–PCR) methodology (37) and calculations (38) to estimate the copy number of the *pART27::COVID-19_S1* vector integrated into the transformed tomato genomes. We cloned the *Sl_Lat*52 (35) gene fragment of tomato into the plasmid vector pCR4 TOPO-TA following the manufacturer’s protocol and instructions (Invitrogen, Waltham, MA USA), which contains the *NPTII* gene as a selectable marker. This way, a reference plasmid vector containing both *NPTII* and *Sl_Lat52* genes was obtained for constructing a standard curve based on absolute copies of the plasmid vector. 10-fold serial dilutions of the plasmid vector and amplified target genes were made before qRT–PCR.

qRT–PCR was carried out in a CFX96 Touch Real-Time PCR Detection System (Bio-Rad, Hercules, CA USA). PCR reactions were performed in a 12.5-μl volume with the following standard program recommended by the manufacturer: 95 °C for 10 min, followed by 40 cycles of 95 °C for 15 s, 60 °C for 1 min, and 72 °C for 01 min. Each 12.5 μl reaction mixture contained 6.25 μl Master Mix (2x), 0.25 μl (200 nM) of each primer (10 μM), one μl (40 nM) of probe (0.5 μM), four μl of template DNA sample (0.2 pg–20 ng) and 0.75 μl sterile deionized water.

Average C_t_ values were plotted against a log of absolute copy numbers to obtain standard curves. Six replicates were conducted to construct standard curves for each target gene. Efficiencies of amplification were calculated based on slopes of standard curves with the following formula: E = 10^(−1/slpoe)^−1 (38–40). The copy-number calculation was performed as described by Weng *et al.* (2004) (39) using the equation X_0_/R_0_ = 10[(Ct, X−IX)/SX)]−[(Ct, R−IR)/SR], where X is *nptII*, R is *Sl_Lat52*, I is an intercept of the standard curve, S is the slope of the standard curve for a target (X) and reference (R) genes (39). X_0_/R_0_ values were used directly (that is, without doubling) for copy-number estimation since our samples were T_1_-generation plants, as was the case in Weng *et al.* (2004) (39). The coefficient of variation in copy-number estimates was calculated from the coefficient of variation estimates for *Sl_Lat52* (endogenous control) and *NPTII* (target gene).

**Isolation of total RNA and RT-PCR**

Total RNA was isolated from tomato leaves using a combination of the protocols of Suzuki *et al.* (2001) (41) and Wu *et al.* (2002) (42). RNA integrity was checked and judged by the presence and intensity of ribosomal RNA bands on a 1% agarose gel containing 2.2 M formaldehyde in the presence of ethidium bromide. To purify samples from DNA impurities, RNA samples were treated with RNase-Free DNase Set (QIAGEN, Hilden, Germany) according to the manufacturer's instructions and then subjected to additional purification with phenol-chloroform (5:1; Ambion, Naugatuck, CT, USA) and ethanol precipitation. Total RNA concentration was measured with a NanoPhotometer N60 Touch Mobile (BioCat, Barcelona, Spain). The purity of the isolated RNA was determined spectrophotometrically by the ratios A_260/280_ and A_260/230_.

For analysis of target gene expression by reverse transcription (RT-PCR), complementary DNA (cDNA) was synthesized from total RNA samples by reverse transcriptase using the SuperScript™ III Reverse Transcriptase cDNA synthesis kit (Thermo Fisher, Waltham, MA USA) with random nonameric primers in manufacturer's protocol. To test for DNA contamination, PT-PCR reactions were performed with intron-specific A1341F/R primer pairs (43), following Blast verification of the target gene in the tomato genome (data not shown).

According to the manufacturer's instructions, a one-step reverse transcription reaction (RT-PCR) was performed using the SuperScript™ III Reverse Transcriptase kit (Thermo Fisher, Waltham, MA USA). The gene expression was determined by quantitative reverse transcription PCR using SYBR Green amplicon detection and the iTaq Universal SYBR Green Supermix kit (Bio-Rad, Hercules, CA USA). RT-PCR was performed on the CFX96 Touch Real-Time PCR Detection System (Bio-Rad, Hercules, CA USA) in a volume of 25 µl with the following cycling profile: 95°C for 10 min followed by 40 cycles at 95°C for 15 s and 65°C for 45 s. Each 25 µl reaction mixture contained 12.5 µl SYBR Green Master Mix (2×), 0.35 µl (140 nM) of each primer (10 µM), 6 µl cDNA template diluted 1:15, and 5.8 µl of sterile deionized water. The 2×SYBR Green PCR master mix contained No AmpErase UNG, AmpliTaq Gold DNA polymerase, deoxynucleoside triphosphates with dUTP, and SYBR Green magnesium chloride reaction buffer (Applied Biosystems, Waltham, MA USA).

Dissociation curves after real-time PCR were plotted for each primer pair and used to exclude primer dimers, genomic DNA contamination, and improper annealing. To prevent false negative results, a pair of primers specific for the plant mitochondrial mRNA NADH-ubiquinone oxidoreductase chain 5 (NAD5) was used to generate a 185 bp fragment. as an internal control PT-PCR. All reactions were performed in three technical replicates. The 2^-ΔΔCt^ method was used to calculate the relative expression of differentially expressed genes (44). All primers were designed using Primer3 and IDT OligoAnalyzer Tool of Integrated DNA Technologies Inc. (Coralville, IA, USA).

**Western blot**

Fresh (40 g) leaves were homogenized for protein purifications in 80 ml of extraction buffer (protease inhibitors cocktail added 1x Phosphate Buffered Saline, Sigma-Aldrich, St. Louis, MO USA), using mortar and pestle on ice. After homogenization, the slurry was centrifuged at 8,000g for 20 min at 4℃. After sequential microfiltration through 0.45 and 0.22 µm Minisart filters (Sartorius, Göttingen, Germany), the fluid was directly used for western blotting analysis. For separation and loading, equal amounts of protein (20–30 μg) were loaded into the 12.5% SDS-PAGE gel wells, along with a molecular weight marker. The gel was run for two hours at 100 V. The nitrocellulose membrane (Bio-Rad, Hercules, CA, USA) was used to transfer the protein from the gel.

For the antibody staining, the membrane was blocked for one hour at room temperature or overnight at four °C using a blocking buffer. The membrane was incubated in a blocking buffer with appropriate dilutions of primary anti-6xHistag antibody produced in Rabbit (Thermo Fisher, Waltham, MA, USA). Then, the membrane was washed with Tris Buffered Saline with Tween™ 20 (TBS-Tween; Sigma-Aldrich, St. Louis, MO USA) three times/15 minutes each. The membrane was incubated with the recommended dilution of conjugated secondary anti-rabbit AP antibody produced in goats (Thermo Fisher, Waltham, MA, USA) in a one-hour blocking buffer at room temperature. After this, it was rewashed with TBS-Tween solution three times/15 minutes each. We followed the kit manufacturer’s recommendations for signal development, removing the excess reagent and covering the membrane in transparent plastic wrap. An image was acquired using darkroom photographic development techniques for chemiluminescence detection (Supplemental Figs S2 and S3).

**ELISA analysis**

To detect the expression of antigenic proteins, ELISA analysis was used, which was carried out using the SARS-CoV-2 Spike S1-RBD IgG ELISA Detection Kit (GenScript, Piscataway, New Jersey, USA) in accordance with the manufacturer's instructions. To determine the S1 protein concentration in transformed tomatoes, a novel coronavirus (2019-CoV) IgG antibody detection kit, Wantai Biopharm (Beijing, China), was used.

**Concentration calculations**

To calculate synthesized amounts of S1 protein in tomato tissues, we created a standard curve using the known concentration of RBD-domain pure protein (ZF-UZ-VAC2001) (28, 33). S1 protein concentration of samples was calculated using *y = mx-c* where *x* is the OD_450nm_ of a sample with unknown concentration from ELISA analysis (Supplementary Table 2).

**Standard diet for mice**

Mice were kept in plastic cages under a temperature of 20-24°C with a relative air humidity of not more than 65% and fed on a standard diet. The standard daily diet (Private animal laboratory, Tashkent, Uzbekistan) contained 4 g cheese, 4 g carrot, 5 g bread, 1ml milk, 0.1 g salt, 1 g beet, 2 g potato, 1 g oatmeal, 2 ml vitamin-mineral supplements, 19 g special feed with vitamin-mineral additives, 2 g dog food, and 20 hay.

**Collection of** **intestinal lavage fluid samples**

Intestinal lavage fluid was collected on days 14, 28, 42 and 56. Animals were anesthetized by ether and sacrificed by decapitation. The small intestine was removed and flushed with 5 mL of PBS supplemented with a 1% (v/v) anti-protease cocktail (45,46). The intestinal content was rejected. The remaining intestinal content suspension was used to flush the small intestine twice. Intestinal fluid suspensions were centrifuged (2000×g, 30 min, 4° C). Aliquots of supernatant (5 ml) were frozen at -70° C for secretory immunoglobulin A (S-IgA) quantification by ELISA (45,46), using Mouse Anti-2019 nCoV(S)IgA ELISA Kit (Wuhan Fine Biotech Co., Ltd., Wuhan, China) according to the manufacturer's instructions.

**Data analysis (detailed)**

*Expression and ELISA data analysis*

Three biological replication materials were collected for expression and ELISA analyses from each plant/tissue. Total RNAs/proteins were isolated and purified, and qPCR/ELISA was run with three technical repeats per tissue/plant. Descriptive data statistics with means, standard deviations, and standard error of means were calculated. Statistical significance of observed differences between plants/tissues was analyzed and visualized using ordinary one-way ANOVA with Tukey’s multiple comparisons using GraphPad Prism version 8.0.1 for Windows (www.graphpad.com; GraphPad Software, San Diego, California, USA).

*Mice data analysis*

Blood serum NAbs and S-IgA level data, collected from the analyses of days 14 (n = 6 mice), day 28 (n = 6 mice), day 42 (n = 6 mice), and day 56 (n = 4 mice), were organized as a column data format and loaded into the GraphPad Prism version 8.0.0 for Windows. It should be mentioned that at week eight (day 56) after oral immunization, mice were 90 days old, and we observed the natural death of two mice per group, including controls. Therefore, for day 56, we collected data from only four mice (n = 4).

All data were first checked for statistical significance between groups for normal distribution using the Shapiro-Wilk test (α = 0.05). Data recorded from the blood serum NAbs and S-IgA level analyses on days 14 and 28 did not pass the Shapiro-Wilk test (α = 0.05). To apply ordinary one-way ANOVA and to achieve a better structure of normal distribution, all data, including those violating normal distribution from days 14 (p = 0.004) and 28 (p = 0.001), were normalized using Log10 transformation, where Log10(x+1) calculation has been applied with data set having zero value. We observed 7.5 and 80-fold normality improvement after Log10 transformation on days 14 and 28 data, respectively, although it remained statistically significant in the Shapiro-Wilk normality test (p = 0.03 and 0.02). This improvement helped to perform ordinary one-way ANOVA and preliminarily analyze the statistical significance of the differences observed in these data sets that could be compared and judged based on the nonparametric Kruskal Wallis one-way ANOVA.

All ordinary one-way ANOVA analyses were performed using GraphPad Prism’s ‘built-in’ functions, assuming ‘no mating or pairing experimental design’, ‘Gaussian distribution of residuals’, and ‘equal SDs’. GraphPad Prism recommended Tukey’s multiple comparisons test with a single pooled variance used for multiple pairwise comparisons. In that, the mean of each column was compared with the mean of every column, adjusting pairwise p-values for multiple comparisons at a family-wise (type I error) significance of 0.05 (95% confidence interval). As an option, descriptive statistics of the data were also calculated and reported (Supplementary Table 7).

Alternatively, because of the abnormal distribution structure of some data groups, the nonparametric Kruskal Wallis (KW) one-way ANOVA was also performed for all data sets to determine the statistical significance of data not passing the normality test as well as additional statistical evidence to judge significant levels obtained from ordinary one-way ANOVA. Although overall KW tests were significant at p<0.01, the analysis of Nabs and S-IgA levels, as well as neutralization activity of TOMOVAC-induced NAbs for day 56 (n = 4), as mentioned above, due to smaller samples than n = 5 in each group, resulting in nonsignificant pairwise comparisons. Therefore, we interpreted only ordinary one-way ANOVA significance for day 56 data, which passed the Shapiro-Wilk normality test to judge the differences recorded. The summary table, including all these analysis results, is presented in Supplementary Tables 7 and 8 below.

*Human consumption data analysis*

In human consumption data analysis, data were first checked for normal distribution of repeated measurements in TOMAVAC-consuming experimental and non-consuming control groups using the Shapiro-Wilk test (α = 0.05). Data recorded from the blood serum NAbs level analyses from the TOMAVAC-consuming experimental group did not pass the Shapiro-Wilk test (α = 0.05), being highly significant at p ≤ 0.01-0.001. Further, data were attempted to be normalized using Log10 transformation; however, it did not pass the normality test (p ≤ 0.01) even after transformation. Therefore, nonparametric Friedman’s ANOVA was performed using GraphPad Prism’s ‘built-in’ functions, with an experimental design of ‘each row represents matched or repeated measures data” and not assuming ‘Gaussian distribution of residuals.’ GraphPad Prism recommended Dunn’s multiple comparisons tests using statistical hypothesis testing for multiple pairwise comparisons In that, the mean rank of each measurement was compared with the mean rank of the initial day as a single control, adjusting pairwise p-values for multiple comparisons at a family-wise (type I error) significance of 0.05 (95% confidence interval). As an option, descriptive statistics of the data were also calculated and reported.

In contrast, control group data passed the normality test that allowed performing repeated measure (RM) one-way ANOVA, following Dunnett’s multiple comparisons. RM one-way ANOVA was performed using GraphPad Prism’s ‘built-in’ functions, with an experimental design of ‘each row represents matched or repeated measures data” and assuming ‘Gaussian distribution of residuals’ and ‘sphericity’. GraphPad Prism-recommended Dunnett’s multiple comparisons test with a single pooled variance was used for multiple pairwise comparisons. In that, the mean of each experimental measurement column was compared with the mean of the initial day measurement column, adjusting pairwise p-values for multiple comparisons at a family-wise (type I error) significance of 0.05 (95% confidence interval).

Alternatively, nonparametric Friedman’s ANOVA was also performed that detected better significance both on day 14 (p ≤ 0.05) and 21 (p ≤ 0.001). Nonparametric Friedman’s ANOVA was performed with methods and options described above for experimental group data analysis and used for data interpretation in the manuscript. The summary table, including all these analysis results, is presented in Supplementary Tables 9 and 10 as well as Supplemental Fig. S4 below.


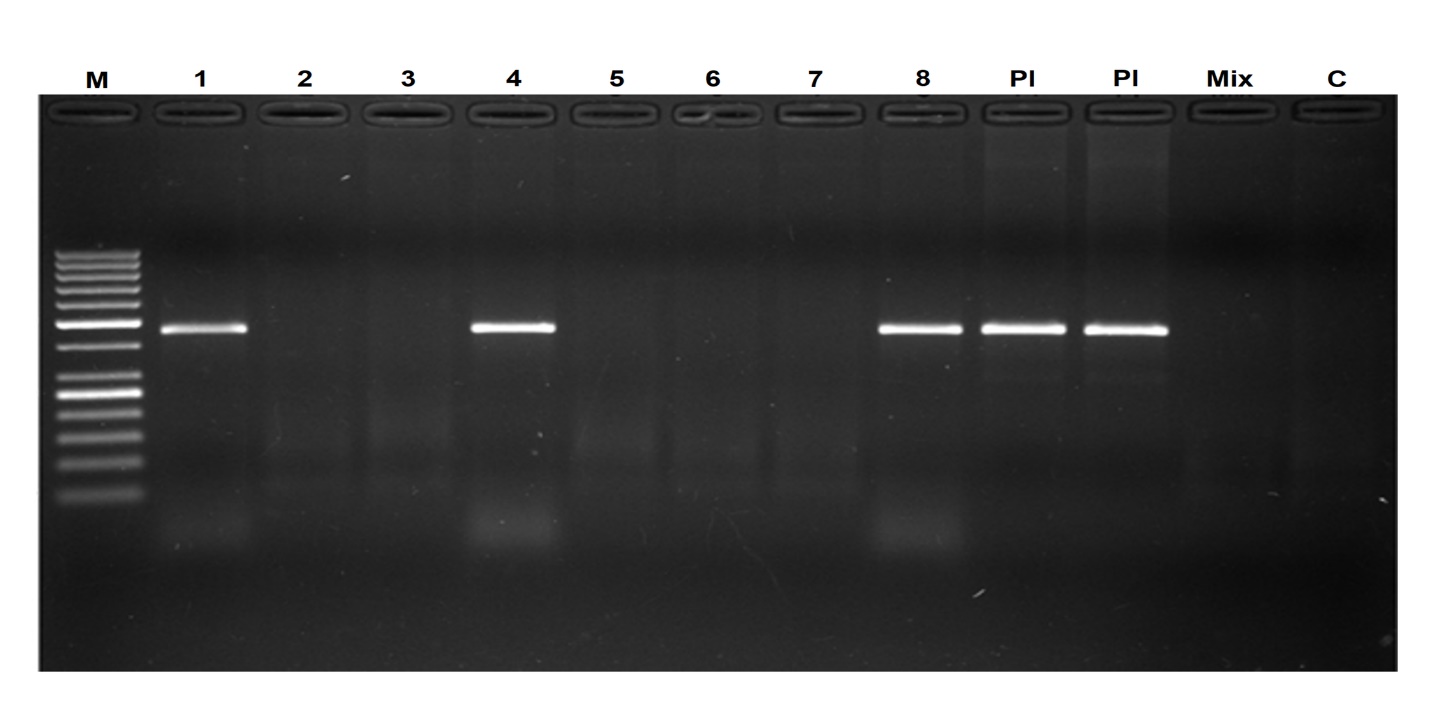


**Supplementary Figure S1.** Original agarose gel image for PCR-analysis of transformed tomatoes. **M** - GeneRuler 50 bp DNA Ladder (Thermo Fisher, Waltham, MA USA); **1-8** – transformed tomato plant DNA; **Pl** – S1 plasmid DNA (a positive control); **Mix** – Master mix without DNA (a negative control); **C** – a negative untransformed tomato plant DNA control.

**
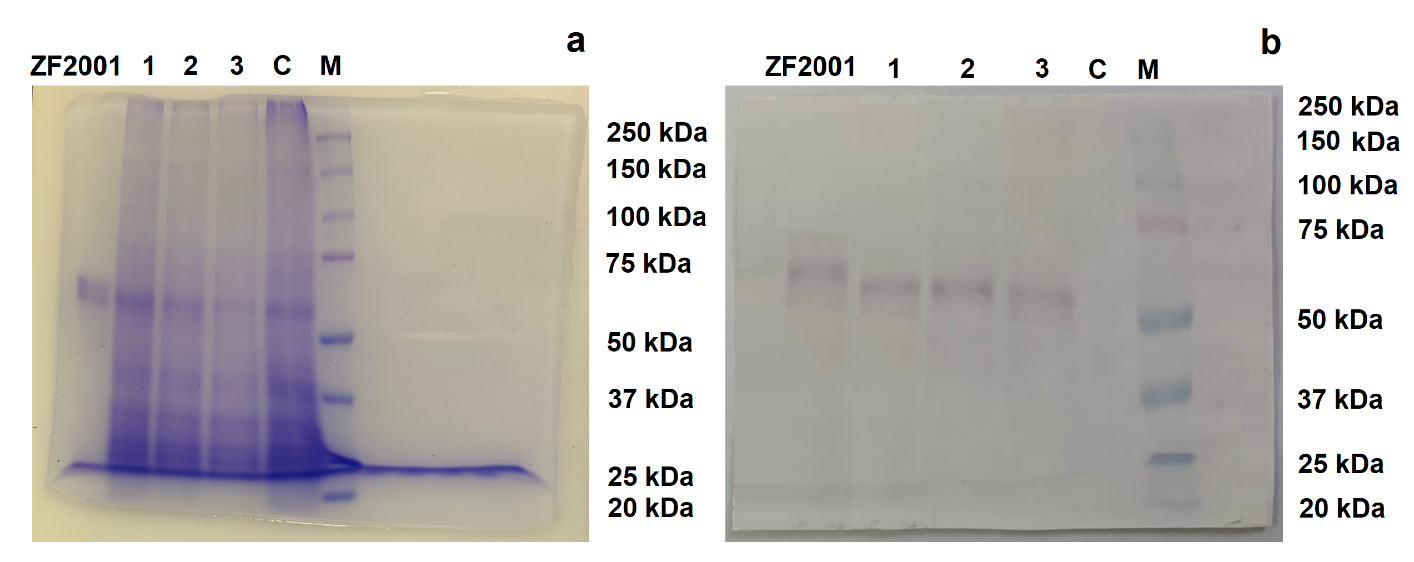
**

**Supplementary Figure S2.** Original western blot gel **(a)** and membrane **(b)** images for expressed protein in transgenic tomato genotypes (1–event 1, 2– event 4, and 3– event 8). RBD protein (ZF-UZ-VAC2001 (23,28) with 61.7 kDa) and untransformed tomatoes **(C)** were used as positive and negative controls. M-Precision Plus Protein Dual Color Standards, 10-250 кD.

**
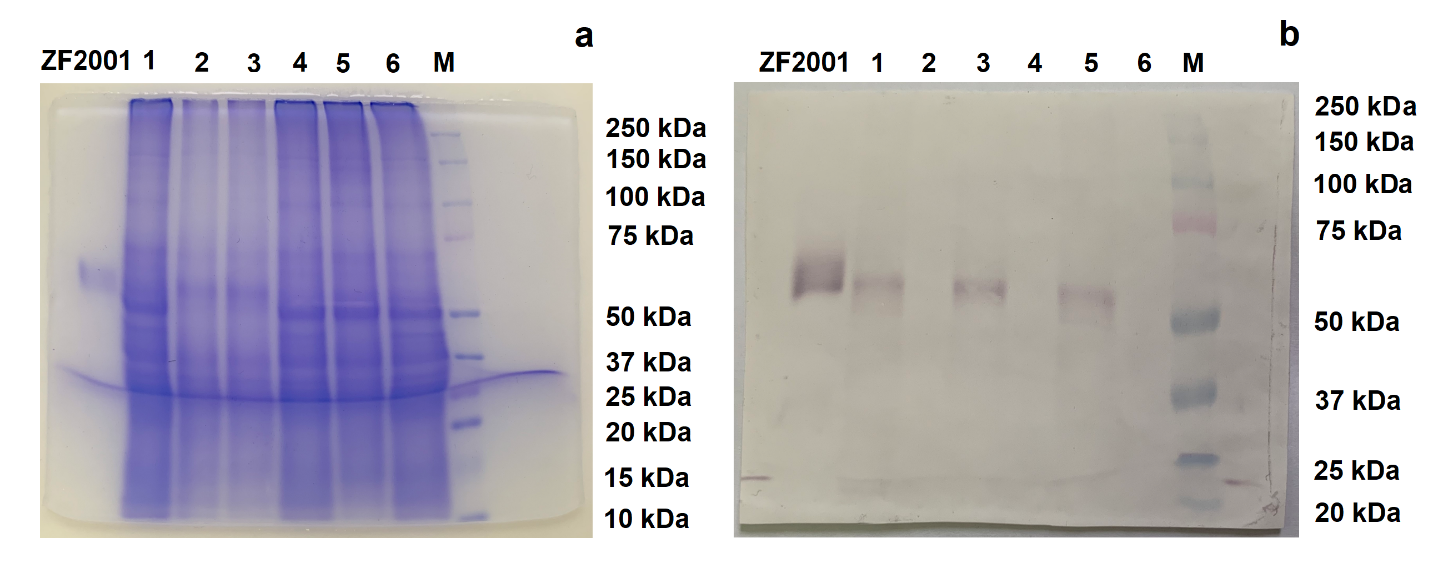
**

**Supplementary Figure S3.** Original western blot gel **(a)** and membrane **(b)** images in different tissues of T_1_-generation (event 4) tomato plants: leaf tissue (1), unripened green tomato (2), ripened red tomato (3), and control tissues from untransformed tomato (2, 4, and 6). RBD protein (ZF-UZ-VAC2001 (23,28) with 61.7 kDa) and untransformed tomatoes were used as positive and negative controls. M-Precision Plus Protein Dual Color Standards, 10-250 кD.


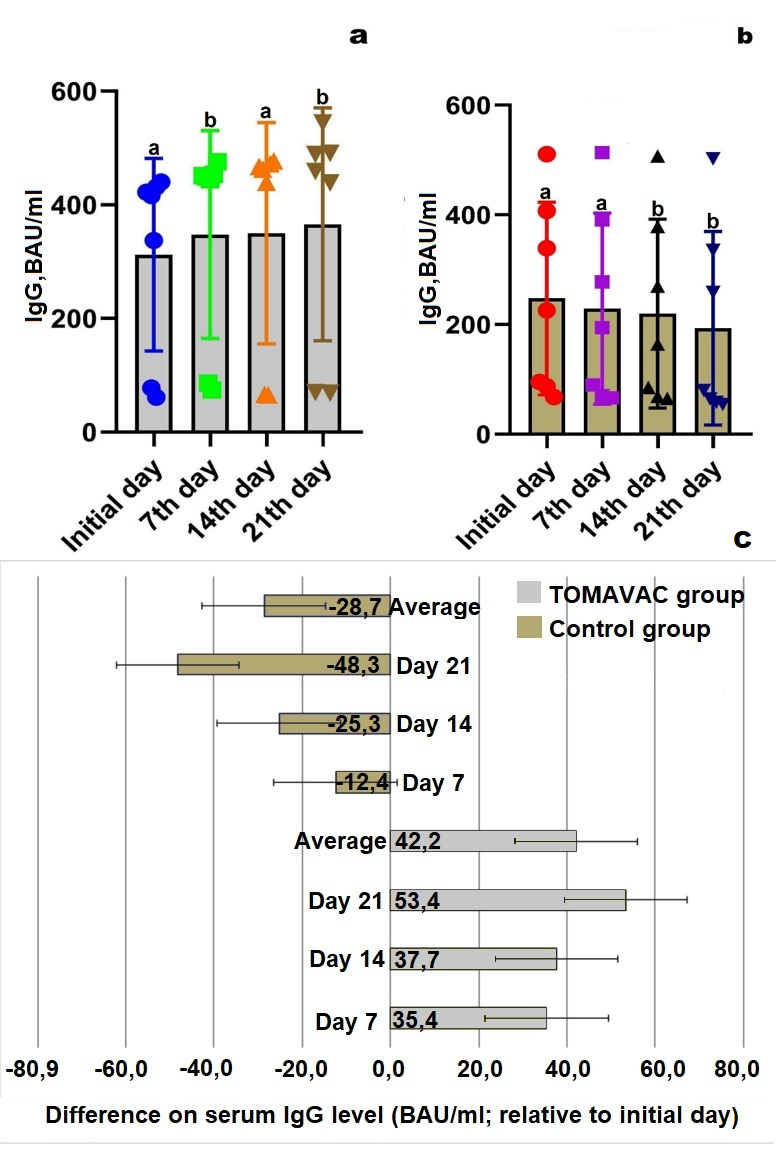


**Supplementary Figure S4.** A comparative titer of RBD-specific serum IgG in volunteers after vaccination. (**a**) – average serum IgG level (BAU/ml) in the TOMAVAC group; (**b**) – average serum IgG level (BAU/ml) in the control group; and (**c**) – the difference in serum IgG levels of TOMAVAC consumed and not consumed groups (BAU/ml; relative to initial day serum IgG of respective groups). Different letters (a and b) show statistical significance at p≤0.05 (relative to initial day NAbs level), using Friedman’s ANOVA (Supplementary Table 9).

**Supplementary Table 1.** Relative expression of S1 protein in tomatoes

| **No** | **Samples** | **Size** | **Min.** | **Max.** | **Mean** | **SD** | **SEM** | **Shapiro-Wilk test p-value (α = 0.05)** | **One-way ANOVA. summary** | **Adjusted P from Tukey’s multiple comparisons** | **Copy-number of the vector** |
| --- | --- | --- | --- | --- | --- | --- | --- | --- | --- | --- | --- |
| **Relative expression levels for independent transformation event tomato seedling tissues (liner)** | | | | | | | | | | |  |
| 1. | Event 1 | 3 | 1.51 | 1.75 | 1.63 | 0.12 | 0.95 | 0.70 | F = 59.01  P <0.0001  Rsq = 0.96 | 1 vs. 2 < 0.001 | 3 |
| 2. | Event 4 | 3 | 0.65 | 1.01 | 0.84 | 0.18 | 0.49 | 0.64 |  | 1 vs. 3 = 0.001 | 1 |
| 3. | Event 8 | 3 | 0.62 | 1.03 | 0.82 | 0.20 | 0.48 | 0.84 |  | 2 vs. 3 = 0.99 | 1 |
| 4. | Control tomato | 3 | 0.02 | 0.03 | 0.03 | 0.01 | 0.01 | 0.67 |  | 1 vs. 4 < 0.0001  2 vs. 4 < 0.001  3 vs. 4 < 0.001 | 0 |
| **Relative expression levels for different tissues of event 4 (liner)** | | | | | | | | | | |  |
| 1. | S1_tomato leaf | 9 | 0.32 | 1.14 | 0.78 | 0.34 | 0.11 | 0.01 | F = 32.29  P<0.0001  Rsq = 0.77  KW = 42.82  P<0.0001 | 1 vs. 3 = 0.99  1 vs. 3 = 0.99^KW^ | 1 |
| 2. | Leaf control | 9 | 0.02 | 0.06 | 0.04 | 0.01 | 0.01 | 0.48 |  | 2 vs. 1 < 0.0001  2 vs. 1 < 0.05^KW^ | 0 |
| 3. | S1_unripened tomato | 9 | 0.50 | 1.09 | 0.73 | 0.19 | 0.06 | 0.66 |  | 3 vs. 5 = 0.98  3 vs. 5 = 0.99^KW^ | 1 |
| 4. | Unripened control | 9 | 0.02 | 0.03 | 0.02 | 0.003 | 0.00 | 0.002 |  | 4 vs. 3 < 0.0001  4 vs. 3 < 0.001^KW^ | 0 |
| 5. | S1_ripened tomato | 9 | 0.32 | 1.06 | 0.66 | 0.31 | 0.10 | 0.12 |  | 5 vs. 1 = 0.83  5 vs. 1 = 0.99^KW^ | 1 |
| 6. | Ripened control | 9 | 0.02 | 0.03 | 0.02 | 0.003 | 0.00 | <0.0001 |  | 6 vs. 5 < 0.0001  6 vs. 5 < 0.001^KW^ | 0 |

Note: KW = Kruskal Wallis nonparametric ANOVA, used for lognormal data. For relative calculation, control values were set to zero.

**Supplementary Table 2.** Standard curve information for S1 protein amount calculations in transformed tomato samples.

| **No.** | **Known RBD (ZF-UZ-VAC2001) Protein Concentration (µg/ml)** | **Absorbance at OD_450nm_** |
| --- | --- | --- |
| 1. | 100 | 32.6 |
| 2. | 50 | 16.1 |
| 3. | 25 | 8.13 |
| 4. | 12.5 | 4.07 |
| 5. | 6.25 | 2.0375 |
| 6. | 3.125 | 1.01875 |
|  | **m** | **0.3255** |
|  | **c** | **-0.0223** |

**Note**: S1 protein concentration was calculated using *y = mx-c* where *x* is the OD_450nm_ of a sample with unknown concentration from ELISA analysis

**Supplementary Table 3.** Standard curve-based calculations of S1 protein amounts in tomatoes from ELISA ODs.

| **No** | **Samples** | **Size** | **Min.** | **Max.** | **Mean** | **SD** | **SEM** | **Shapiro-Wilk test p-value (α = 0.05)** | **One-way ANOVA. summary** | **Adjusted P from Tukey’s multiple comparisons** | **Copy-number of the vector** |
| --- | --- | --- | --- | --- | --- | --- | --- | --- | --- | --- | --- |
| **S1 antigen amounts for independent transformation event tomato seedling tissues** | | | | | | | | | | | |
| 1. | Event 1 | 3 | 1.10 | 1.67 | 1.38 | 0.29 | 0.167 | 0.98 | F = 41.88  P <0.0001  Rsq = 0.94 | 1 vs. 2 = 0.004 | 3 |
| 2. | Event 4 | 3 | 0.74 | 0.79 | 0.77 | 0.03 | 0.016 | 0.64 |  | 1 vs. 3 = 0.0002 | 1 |
| 3. | Event 8 | 3 | 0.35 | 0.48 | 0.42 | 0.06 | 0.037 | 0.95 |  | 2 vs. 3 = 0.08 | 1 |
| 4. | Control tomato | 3 | 0.07 | 0.09 | 0.08 | 0.01 | 0.004 | >0.99 |  | 1 vs. 4 < 0.0001  2 vs. 4 = 0.002  3 vs. 4 = 0.09 | 0 |
| **S1 antigen amounts for different tissue of event 4** | | | | | | | | | | | |
| 1. | S1_tomato leaf | 3 | 1.077 | 1.707 | 1.40 | 0.32 | 0.18 | 0.91 | F = 67.27  P<0.0001  Rsq = 0.97 | 1 vs. 3 = 0.03 | 1 |
| 2. | Leaf control | 3 | 0.005 | 0.011 | 0.007 | 0.004 | 0.002 | - |  | 1 vs. 2 <0.0001 | 0 |
| 3. | S1_unripened tomato | 3 | 1.783 | 2.411 | 2.17 | 0.34 | 0.20 | 0.26 |  | 3 vs. 5 = <0.0001 | 1 |
| 4. | Unripened control | 3 | 0.010 | 0.037 | 0.02 | 0.02 | 0.01 | - |  | 3 vs. 4 <0.0001 | 0 |
| 5. | S1_ripened tomato | 3 | 0.717 | 0.836 | 0.77 | 0.06 | 0.03 | 0.94 |  | 5 vs. 1 = 0.02 | 1 |
| 6. | Ripened control | 3 | 0.002 | 0.008 | 0.005 | 0.003 | 0.002 | 0.78 |  | 5 vs. 6 = 0.004 | 0 |

**Note**: Supplementary Table 2 data were used for amount calculations.

**Supplementary Table 4.** The primer pairs and probes for PCR amplification, vector construction, and quantitative real-time PCR.

| **Name** | **Orientation** | **Sequence (5'-3')** | **Length(nt)** | **Amplicon size (bp)** | **Reference** |
| --- | --- | --- | --- | --- | --- |
| NTD_1f_qPCR | Forward | CTCAGGACTTGTTCTTACCTTTCT | 24 | 98 | In this study |
| NTD_1r_qPCR | Reverse | GACAGGGTTATCAAACCTCTTAGT | 24 |  |  |
| NTD_1probe | Probe | 6-FAM-TCCATGCTATACATGTCTCTGGGACCA-BHQ1 | 27 |  |  |
| RBD_2F_qPCR | Forward | AGAGGTGATGAAGTCAGACAAAT | 23 | 100 | In this study |
| RBD_2R_qPCR | Reverse | AAGCTATAACGCAGCCTGTAA | 21 |  |  |
| RBD_2probe | Probe | HEX-CCAGGGCAAACTGGAAAGATTGCT-BHQ1 | 24 |  |  |
| NAD-5-F | Forward | GATGCTTCTTGGGGCTTCTTGTT | 23 | 181 | Menzel *et al.*(47) |
| NAD-5-R | Reverse | CTCCAGTCACCAACATTGGCATAA | 24 |  |  |
| NAD-5-Probe | Probe | VIC-AGGATCCGCATAGCCCTCGATTTATGTG-BHQ1 | 28 |  |  |
| LAT52_F | Forward | AGACCACGAGAACGATATTTGC | 22 | 92 | Yang *et al*.(35) |
| LAT52_R | Reverse | TTCTTGCCTTTTCATATCCAGACA | 24 |  |  |
| LAT52_P | Probe | FAM6-CTCTTTGCAGTCCTCCCTTGGGCT-BHQ1 | 24 |  |  |
| 35S-F | Forward | GTGGCTCCTACAAATGCCATCAT | 23 | 135 | In this study |
| 35S-R | Reverse | AAGACGTGGTTGGAACGTCTTC | 22 |  |  |
| probe_35S | Probe | VIC-CAAAGATGGACCCCCACCCACG-BHQ 1 | 22 |  |  |
| LAT52_F_BamH1_XhoI | Forward | aaaggatcctcgagGTTGCAATGCAGGAACATCA | 34 | 249 | In this study |
| LAT52_R_SacI_XbaI | Reverse | aaagagctctctagaATGAAGAAAAGTGGATTGGC | 35 |  |  |
| 4_S1-fragment_F | Forward | TCGAAGACCCAGTCCCTACT | 20 | 553 | In this study |
| 4_S1-fragment_R | Reverse | GAGGGTCAAGTGCACAGTCT | 20 |  |  |

**Note**: NTD – the terminal domain of the spike protein of the SARS-CoV-2; RBD – the receptor binding domain of the SARS-CoV-2; NAD-5 – Internal control primers in the mitochondrial *NADH* *dehydrogenase subunit 5* gene; LAT52 – *LAT52* gene of tomato; 35S – CaMV 35S promoter; and 4_S1- S1 gene of the SARS-CoV-2;

**Supplementary Table 5.** Standard curve for CaMV 35S promoter and tomato-specific *Sl_Lat52* genes from a pART27-Sl_Lat52 plasmid vector.

| **DNA amount** | | **Average**  **Ct values*** | **SD** | **SE** | **CV (%)** | **Slope** | **Intercept** | **R2** | **Efficiency**** |
| --- | --- | --- | --- | --- | --- | --- | --- | --- | --- |
| **ng** | **copy** |  |  |  |  |  |  |  |  |
| **35S promoter** | | | | | | -3.16 | 35.49 | 0.996 | 107% |
| 20 | 1319088116 | 8.45 | 0.11 | 0.05 | 0.01 |  |  |  |  |
| 2 | 131908811 | 12.15 | 0.21 | 0.09 | 0.02 |  |  |  |  |
| 0.2 | 13190881 | 14.30 | 0.45 | 0.19 | 0.03 |  |  |  |  |
| 0.02 | 1319088 | 17.55 | 0.25 | 0.10 | 0.01 |  |  |  |  |
| 0.002 | 131908 | 21.32 | 0.35 | 0.15 | 0.02 |  |  |  |  |
| 0.0002 | 13190 | 24.45 | 0.48 | 0.20 | 0.02 |  |  |  |  |
| **Solanum lycopersicum_LAT52** | | | | | | -3.41 | 34.56 | 0.998 | 96.45% |
| 20 | 1319088116 | 7.56 | 0.12 | 0.05 | 0.02 |  |  |  |  |
| 2 | 131908811 | 11.22 | 0.78 | 0.33 | 0.07 |  |  |  |  |
| 0.20 | 13190881 | 14.25 | 0.42 | 0.18 | 0.03 |  |  |  |  |
| 0.02 | 1319088 | 18.45 | 0.65 | 0.27 | 0.04 |  |  |  |  |
| 0.002 | 131908 | 21.25 | 0.72 | 0.30 | 0.03 |  |  |  |  |
| 0.0002 | 13190 | 24.56 | 0.56 | 0.23 | 0.02 |  |  |  |  |

**Note**: SD – standard deviation; SE – standard error = SD/SQRT_(n)_, where *n* is the sample size. *qRT-PCR reaction for each dilution was repeated six times, and average values were shown in this table. **Efficiency of PCR was calculated using the following formula: E = 10^(-1/Slope)^ -1 and presented in percentage.

**Supplementary Table 6.** Estimated numbers of CaMV 35S promoters in the first-generation (T_1_) tomato lines.

| **Samples** | **Average Ct values*** | **SD** | **SE** | **CV(%)** | **X_0_/R_0_**** | **Estimated copy number** | **CV***** | **CV bruto** |
| --- | --- | --- | --- | --- | --- | --- | --- | --- |
| CaMV 35S Promoter_1 | 24.54 | 0.16 | 0.07 | 0.01 | 3.1015789127473816 | 3 | 0.006634695936334784 | 0.006 |
| CaMV 35S Promoter_4 | 28.73 | 0.21 | 0.09 | 0.01 | 1.1329128898450334 | 1 | 0.010066647960943882 | 0.010 |
| CaMV 35S Promoter_8 | 28.38 | 0.76 | 0.32 | 0.03 | 1.2266189715032605 | 1 | 0.027054929004352853 | 0.027 |
| CaMV 35S Promoter_control | 36.41 | 0.56 | 0.23 | 0.02 | 0.004799196873686667 | 0 | 0.016237701820819836 | 0.016 |
| Lat52_1 | 24.42 | 0.03 |  |  |  |  |  |  |
| Lat52_4 | 27.45 | 0.19 |  |  |  |  |  |  |
| Lat52_8 | 27.19 | 0.16 |  |  |  |  |  |  |
| Lat52_control | 26.89 | 0.14 |  |  |  |  |  |  |

**Note**: SD – standard deviation; SE – standard error [SD/SQRT(n)]; CV-coefficient of variation [SD/mean value]; *qRT-PCR reaction for each sample was repeated six times and averages values were shown in this table; **X_0_/R_0_ = 10^[(Ct, X-IX)/SX)]-[(Ct, R-IR)/SR]^, where X is CaMV 35S promoter*,* R is *Lat52*, I is an intercept of the standard curve, S is the slope of the standard curve for a target (X) and reference (R) genes^37^; ***Coefficient of variation in copy number estimates was calculated from the coefficient of variation estimates for LAT52 (endogenous control) and *CaMV 35 S promoter* (target gene) using s = (cv)(mean value _CaMV 35S promoter_ /mean value_LAT52_), where cv = SQRT[(CV_CaMV 35S promoter_ ) 2 +(CV_LAT52_) 2].

**Supplementary Table 7.** Statistical analysis of NAbs level data from oral immunization of TOMAVAC in mice.

| **No.** | **Groups** | **Size, n** | **Min.** | **Max.** | **Range** | **Mean** | **SD** | **SEM** | **Normality, Shapiro-Wilk test p-value (α = 0.05)** | **One-way ANOVA, summary** | **Adjusted P from Tukey’s multiple comparisons** | **Kruskal Wallis test** | **Adjusted P from Dunn’s multiple comparisons** |
| --- | --- | --- | --- | --- | --- | --- | --- | --- | --- | --- | --- | --- | --- |
| **NAbs level on day 14 (Raw data), day 14** | | | | | | | | | | | | | |
| 1. | TOMAVAC | 6 | 0 | 15439 | 15439 | 5333 | 5563 | 2271 | 0.29 | F = 7.074  P = 0.002  Rsq = 0.51 | 1 vs. 2 = 0.36 | KW = 13.2  P = 0.004 | 1 vs. 2 = 0.99 |
| 2. | Untransformed tomato | 6 | 0 | 4705 | 4705 | 2026 | 1647 | 672 | 0.85 |  | 1 vs. 3 = 0.12 |  | 1 vs. 3 = 0.31 |
|  |  |  |  |  |  |  |  |  |  |  | 1 vs. 4 = 0.26 |  | 1 vs. 4 = 0.75 |
| 3. | Standard diet control | 6 | 0 | 3165 | 3165 | 768 | 1201 | 490 | 0.004 |  | 2 vs. 3 = 0.91 |  | 2 vs. 3 = 0.60 |
|  |  |  |  |  |  |  |  |  |  |  | 2 vs. 4 = 0.009 |  | 2 vs. 4 = 0.07 |
| 4. | AstraZeneca | 6 | 5048 | 14324 | 9276 | 9035 | 3378 | 1379 | 0.85 |  | 3 vs. 4 = 0.002 |  | 3 vs. 4 = 0.003 |
| **NAbs level on day 14 (Normalized with LOG10 transformation), day 14** | | | | | | | | | | | | | |
| 1. | TOMAVAC | 6 | 3.30 | 4.24 | 0.94 | 3.76 | 0.33 | 0.13 | 0.99 | F = 6.2  P = 0.004  Rsq = 0.48 | 1 vs. 2 = 0.46 | KW = 11.0  P = 0.01 | 1 vs. 2 = 0.99 |
| 2. | Untransformed tomato | 6 | 3.30 | 3.83 | 0.52 | 3.57 | 0.18 | 0.07 | 0.95 |  | 1 vs. 3 = 0.06 |  | 1 vs. 3 = 0.23 |
|  |  |  |  |  |  |  |  |  |  |  | 1 vs. 4 = 0.57 |  | 1 vs. 4 = 0.99 |
| 3. | Standard diet control | 6 | 3.30 | 3.71 | 0.41 | 3.42 | 0.15 | 0.06 | 0.03 |  | 2 vs. 3 = 0.61 |  | 2 vs. 3 = 0.99 |
|  |  |  |  |  |  |  |  |  |  |  | 2 vs. 4 = 0.05 |  | 2 vs. 4 = 0.19 |
| 4 | AstraZeneca | 6 | 3.70 | 4.15 | 0.45 | 3.93 | 0.16 | 0.07 | 0.91 |  | 3 vs. 4 = 0.003 |  | 3 vs. 4 = 0.009 |
| **NAbs level on day 28 (Raw data), day 28.** | | | | | | | | | | | | | |
| 1. | TOMAVAC | 6 | 20673 | 193393 | 172721 | 55505 | 68027 | 27772 | 0.001 | Not performed | - | KW = 17.0  P = 0.001 | 1 vs. 2 = 0.006 |
| 2. | Untransformed tomato | 6 | 1780 | 13685 | 11905 | 7375 | 4757 | 1942 | 0.71 |  | - |  | 1 vs. 3 = 0.001 |
|  |  |  |  |  |  |  |  |  |  |  | - |  | 1 vs. 4 = 0.76 |
| 3. | Standard diet control | 6 | 3272 | 9926 | 6654 | 5838 | 2934 | 1198 | 0.08 |  | - |  | 2 vs. 3 = 0.99 |
|  |  |  |  |  |  |  |  |  |  |  | - |  | 2 vs. 4 = 0.43 |
| 4 | AstraZeneca | 6 | 10582 | 24469 | 13887 | 14669 | 5204 | 2124 | 0.08 |  | - |  | 3 vs. 4 = 0.18 |
| **NAbs level on day 28 (Normalized with LOG10 transformation), day 28** | | | | | | | | | | | | | |
| 1. | TOMAVAC | 6 | 4.36 | 5.29 | 0.94 | 4.60 | 0.36 | 0.15 | 0.02 | F = 11.63  P = 0.0001  Rsq = 0.63 | 1 vs. 2 = 0.0004 | KW = 15.6  P = 0.001 | 1 vs. 2 = 0.0076 |
| 2. | Untransformed tomato | 6 | 3.58 | 4.20 | 0.62 | 3.92 | 0.25 | 0.10 | 0.65 |  | 1 vs. 3 = 0.0002 |  | 1 vs. 3 = 0.0027 |
|  |  |  |  |  |  |  |  |  |  |  | 1 vs. 4 = 0.0182 |  | 1 vs. 4 = 0.4751 |
| 3. | Standard diet control | 6 | 3.72 | 4.08 | 0.35 | 3.87 | 0.15 | 0.06 | 0.16 |  | 2 vs. 3 = 0.9855 |  | 2 vs. 3 = 0.99 |
|  |  |  |  |  |  |  |  |  |  |  | 2 vs. 4 = 0.3710 |  | 2 vs. 4 = 0.8499 |
| 4. | Astrazeneca | 6 | 4.025 | 4.389 | 0.3640 | 4.14 | 0.1356 | 0.05535 | 0.28 |  | 3 vs. 4 = 0.2204 |  | 3 vs. 4 = 0.4751 |
| **NAbs level on day 42 (Raw data), day 42.** | | | | | | | | | | | | | |
| 1. | TOMAVAC | 6 | 19731 | 69937 | 50206 | 43013 | 21949 | 8961 | 0.27 | F = 16.77  P<0.0001  Rsq = 0.71 | 1 vs. 2 = 0.0008 | KW = 17.4  P<0.001 | 1 vs. 2 = 0.02 |
| 2. | Untransformed tomato | 6 | 0 | 6015 | 6015 | 2899 | 2418 | 987,3 | 0.60 |  | 1 vs. 3 = 0.0010 |  | 1 vs. 3 = 0.03 |
|  |  |  |  |  |  |  |  |  |  |  | 1 vs. 4 = 0.87 |  | 1 vs. 4 = 0.99 |
| 3. | Standard diet control | 6 | 0 | 9344 | 9344 | 3879 | 4015 | 1639 | 0.23 |  | 2 vs. 3 = 0.99 |  | 2 vs. 3 = 0.99 |
|  |  |  |  |  |  |  |  |  |  |  | 2 vs. 4 = 0.0001 |  | 2 vs. 4 = 0.01 |
| 4. | Astrazeneca | 6 | 16425 | 68053 | 51628 | 49576 | 19625 | 8012 | 0.41 |  | 3 vs. 4 = 0.0002 |  | 3 vs. 4 = 0.02 |
| **NAbs level on day 42 (Normalized with LOG10 transformation), day 42** | | | | | | | | | | | | | |
| 1. | TOMAVAC | 6 | 4.34 | 4.86 | 0.52 | 4.61 | 0.22 | 0.09 | 0.40 | F = 27.42  P<0.0001  Rsq = 0.80 | 1 vs. 2 <0.0001 | KW = 17.3  P<0.001 | 1 vs. 2 = 0.02 |
| 2. | Untransformed tomato | 6 | 3.30 | 3.90 | 0.60 | 3.64 | 0.25 | 0.10 | 0.45 |  | 1vs. 3 <0.0001 |  | 1 vs. 3 = 0.03 |
|  |  |  |  |  |  |  |  |  |  |  | 1 vs. 4 = 0.99 |  | 1 vs. 4 = 0.99 |
| 3. | Standard diet control | 6 | 3.30 | 4.06 | 0.75 | 3.67 | 0.34 | 0.14 | 0.17 |  | 2 vs. 3 = 0.99 |  | 2 vs. 3 = 0.99 |
|  |  |  |  |  |  |  |  |  |  |  | 2 vs. 4 <0.0001 |  | 2 vs. 4 = 0.01 |
| 4. | Astrazeneca | 6 | 4.215 | 4.833 | 0.6174 | 4.653 | 0.2329 | 0.0951 | 0.07 |  | 3 vs. 4 <0.0001 |  | 3 vs. 4 = 0.02 |
| **NAbs level on day 56 (Raw data), day 56** | | | | | | | | | | | | | |
| 1. | TOMAVAC | 4 | 61470 | 98358 | 36888 | 80858 | 16783 | 8392 | 0.71 | F = 47.82  P<0.0001  Rsq = 0.92 | 1 vs. 2 <0.0001 | KW = 11.3  P = 0.0011 | 1vs. 2 = 0.13 |
| 2. | Untransformed tomato | 4 | 2017 | 7827 | 5811 | 4971 | 2637 | 1318 | 0.74 |  | 1 vs. 3 <0.0001 |  | 1vs. 3 = 0.13 |
|  |  |  |  |  |  |  |  |  |  |  | 1 vs. 4 = 0.99 |  | 1 vs. 4 = 0.99 |
| 3. | Standard diet control | 4 | 2944 | 7133 | 4189 | 5175 | 1772 | 885,8 | 0.97 |  | 2 vs. 3 = 0.99 |  | 2 vs. 3 = 0.99 |
|  |  |  |  |  |  |  |  |  |  |  | 2 vs. 4 <0.0001 |  | 2vs. 4 = 0.09 |
| 4. | Astrazeneca | 4 | 66089 | 110061 | 43972 | 83091 | 19185 | 9593 | 0.47 |  | 3 vs. 4<0.0001 |  | 3 vs. 4 = 0.09 |
| **NAbs level on day 56 (Normalized with LOG10 transformation), day 56** | | | | | | | | | | | | | |
| 1. | TOMAVAC | 4 | 4.80 | 5.00 | 0.20 | 4.91 | 0.09 | 0.05 | 0.69 | F = 101.9  P<0.0001  Rsq = 0.96 | 1 vs. 2 <0.0001 | KW = 11.3  P<0.001 | 1 vs. 2 = 0.1049 |
| 2. | Untransformed tomato | 4 | 3.60 | 3.99 | 0.39 | 3.82 | 0.18 | 0.09 | 0.71 |  | 1 vs. 3 <0.0001 |  | 1 vs. 3 = 0.1049 |
|  |  |  |  |  |  |  |  |  |  |  | 1 vs. 4 = 0.99 |  | 1 vs. 4 = 0.99 |
| 3. | Standard diet control | 4 | 3.69 | 3.96 | 0.27 | 3.85 | 0.11 | 0.06 | 0.83 |  | 2 vs. 3 = 0.98 |  | 2 vs. 3 = 0.99 |
|  |  |  |  |  |  |  |  |  |  |  | 2 vs. 4 <0.0001 |  | 2 vs. 4 = 0.1049 |
| 4. | Astrazeneca | 4 | 4.820 | 5.042 | 0.2215 | 4.911 | 0.0952 | 0.04763 | 0.68 |  | 3 vs. 4 <0.0001 |  | 3 vs. 4 = 0.1049 |
| **Neutralization activity of induced IgG (Raw data), day 42** | | | | | | | | | | | | | |
| 1. | TOMAVAC | 6 | 2.56 | 25.64 | 23.08 | 17.33 | 8.27 | 3.38 | 0.30 | F = 32.12  P<0.0001  Rsq = 0.82 | 1 vs. 2 = 0.0014 | KW = 17.2  P = 0.0007 | 1 vs. 2 = 0.26 |
| 2. | Untransformed tomato | 6 | 0,00 | 8,21 | 8,21 | 3,21 | 3,87 | 1,58 | 0.06 |  | 1 vs. 3 = 0.0010 |  | 1 vs. 3 = 0.28 |
|  |  |  |  |  |  |  |  |  |  |  | 1 vs. 4 = 0.0056 |  | 1 vs. 4 = 0.85 |
| 3. | Standard diet control | 6 | 0.64 | 7.05 | 6.41 | 2.68 | 2.35 | 0.96 | 0.14 |  | 2 vs. 3 = 0.99 |  | 2 vs. 3 = 0.99 |
|  |  |  |  |  |  |  |  |  |  |  | 2 vs. 4<0.0001 |  | 2 vs. 4 = 0.003 |
| 4. | Astrazeneca | 6 | 24.36 | 37.95 | 13.59 | 29.51 | 5.833 | 2.381 | 0.10 |  | 3 vs. 4 <0.0001 |  | 3 vs. 4 = 0.003 |
| **Neutralization activity of induced IgG (Normalized with LOG10 transformation), day 42** | | | | | | | | | | | | | |
| 1. | TOMAVAC | 6 | 1.10 | 1.55 | 0.45 | 1.41 | 0.16 | 0.07 | 0.06 | F = 11.69  P = 0.0001  Rsq = 0.63 | 1 vs. 2 = 0.0012 | KW = 15.1  P<0.0018 | 1 vs. 2 = 0.0539 |
| 2. | Untransformed tomato | 6 | 1.00 | 1.26 | 0.26 | 1.11 | 0.12 | 0.05 | 0.05 |  | 1 vs. 3 = 0.0233 |  | 1 vs. 3 = 0.0607 |
|  |  |  |  |  |  |  |  |  |  |  | 1 vs. 4 = 0.96 |  | 1 vs. 4 = 0.99 |
| 3. | Standard diet control | 6 | 1.03 | 1.23 | 0.21 | 1.10 | 0.07 | 0.03 | 0.30 |  | 2 vs. 3 = 0.56 |  | 2 vs. 3 = 0.99 |
|  |  |  |  |  |  |  |  |  |  |  | 2 vs. 4 = 0.0004 |  | 2 vs. 4 = 0.0225 |
| 4. | Astrazeneca | 6 | 1.38 | 1.58 | 0.19 | 1.46 | 0.08 | 0.03 | 0.14 |  | 3 vs. 4 = 0.008 |  | 3 vs. 4 = 0.0256 |
| **Neutralization activity of induced IgG (Raw data), day 56** | | | | | | | | | | | | | |
| 1. | TOMAVAC | 4 | 15.51 | 34.17 | 18.66 | 25.44 | 7.85 | 3.92 | 0.96 | F = 43.70  P<0.0001  Rsq = 0.91 | 1 vs. 2 = 0.0063 | KW = 13.5  P<0.001 | 1 vs. 2 = 0.99 |
| 2. | Untransformed tomato | 4 | 6.10 | 10.85 | 4.75 | 8.60 | 2.35 | 1.17 | 0.31 |  | 1 vs. 3 = 0.0011 |  | 1 vs. 3 = 0.16 |
|  |  |  |  |  |  |  |  |  |  |  | 1 vs. 4 = 0.0013 |  | 1 vs. 4 = 0.99 |
| 3. | Standard diet control | 4 | 0.34 | 7.83 | 7.49 | 4.42 | 3.10 | 1.55 | 0.80 |  | 2 vs. 3 = 0.73 |  | 2 vs. 3 = 0.99 |
|  |  |  |  |  |  |  |  |  |  |  | 2 vs. 4<0.0001 |  | 2 vs. 4 = 0.07 |
| 4. | Astrazeneca | 4 | 38.32 | 55.45 | 17.14 | 46.04 | 7.391 | 3.695 | 0.89 |  | 3 vs. 4<0.0001 |  | 3 vs. 4 = 0.004 |
| **Neutralization activity of induced IgG (Normalized with LOG10 transformation), day 56** | | | | | | | | | | | | | |
| 1. | TOMAVAC | 4 | 1.41 | 1.65 | 0.24 | 1.54 | 0.10 | 0.05 | 0.84 | F = 31.92  P<0.0001  Rsq = 0.88 | 1 vs. 2 = 0.0028 | KW = 12.9  P<0.0001 | 1 vs. 2 = 0.8249 |
| 2. | Untransformed tomato | 4 | 1.21 | 1.32 | 0.11 | 1.27 | 0.06 | 0.03 | 0.32 |  | 1 vs. 3 = 0.0001 |  | 1 vs. 3 = 0.1049 |
|  |  |  |  |  |  |  |  |  |  |  | 1 vs. 4 = 0.2408 |  | 1 vs. 4 = 0.99 |
| 3. | Standard diet control | 4 | 1.01 | 1.25 | 0.24 | 1.15 | 0.10 | 0.05 | 0.62 |  | 2 vs. 3 = 0.2520 |  | 2 vs. 3 = 0.99 |
| 4. | Astrazeneca | 4 | 1.583 | 1.744 | 0.1605 | 1.659 | 0.06901 | 0.03451 | 0.95 |  | 2 vs. 4 = 0.0001 |  | 2 vs. 4 = 0.1049 |

**Note**: based on these analyses, the statistical significance levels were reported as * – ≤ 0.05, ** –≤ 0.01, and *** – ≤ 0.001 for ordinary one-way ANOVA analysis, and KW* – ≤ 0.05, KW**– ≤ 0.01, KW***– ≤ 0.001) for Kruskal Wallis (KW) one-way ANOVA. SD is the standard deviation; SEM is the standard error of the mean.

**Supplementary Table 8.** Statistical analysis of S-IgA level data from oral immunization of TOMAVAC in mice.

| **No.** | **Groups** | **Size, n** | **Min.** | **Max.** | **Range** | **Mean** | **SD** | **SEM** | **Normality, Shapiro-Wilk test p-value (α = 0.05)** | **One-way ANOVA, summary** | **Adjusted P from Tukey’s multiple comparisons** | **Kruskal Wallis test** | **Adjusted P from Dunn’s multiple comparisons** |
| --- | --- | --- | --- | --- | --- | --- | --- | --- | --- | --- | --- | --- | --- |
| **S-IgA** **level on day 14 (Raw data), day 14** | | | | | | | | | | | | | |
| 1 | TOMAVAC | 6 | 4.50 | 6.80 | 2.30 | 5.90 | 0.95 | 0.39 | 0.20 | F=121.4  P<0.0001  Rsq=0.95 | 1 vs 2 <0.0001 | KW=19.47  P<0.001 | 1 vs 2 =0.001 |
| 2 | Untransformed tomato | 6 | 0.32 | 0.57 | 0.26 | 0.38 | 0.10 | 0.04 | 0.02 |  | 1 vs 3 <0.0001 |  | 1 vs 3 =0.002 |
|  |  |  |  |  |  |  |  |  |  |  | 1 vs 4 <0.0001 |  | 1 vs 4 =0.85 |
| 3 | Standard diet control | 6 | 0.31 | 0.39 | 0.08 | 0.36 | 0.03 | 0.01 | 0.61 |  | 2 vs 3 = 0.99 |  | 2 vs 3 >0.99 |
|  |  |  |  |  |  |  |  |  |  |  | 2 vs 4 =0.0004 |  | 2 vs 4 = 0.16 |
| 4 | AstraZeneca | 6 | 1.10 | 3.10 | 2.00 | 2.00 | 0.65 | 0.26 | 0.54 |  | 3 vs 4 =0.0003 |  | 3 vs 4 =0.17 |
| **S-IgA** **level on day 28 (Raw data), day 28** | | | | | | | | | | | | | |
| 1 | TOMAVAC | 6 | 5.80 | 8.90 | 3.10 | 7.50 | 1.10 | 0.45 | 0.80 | F=166.1  P<0.0001  Rsq=0.96 | 1 vs 2 <0.0001 | KW=20  P<0.001 | 1 vs 2 =0.003 |
| 2 | Untransformed tomato | 6 | 0.11 | 0.65 | 0.54 | 0.37 | 0.20 | 0.08 | 0.97 |  | 1 vs 3 <0.0001 |  | 1 vs 3 <0.001 |
|  |  |  |  |  |  |  |  |  |  |  | 1 vs 4 <0.0001 |  | 1 vs 4 =0.85 |
| 3 | Standard diet control | 6 | 0.04 | 0.70 | 0.67 | 0.32 | 0.23 | 0.10 | 0.85 |  | 2 vs 3 = 0.99 |  | 2 vs 3 >0.99 |
|  |  |  |  |  |  |  |  |  |  |  | 2 vs 4 <0.0001 |  | 2 vs 4 = 0.25 |
| 4 | AstraZeneca | 6 | 2.00 | 3.20 | 1.20 | 2.50 | 0.56 | 0.23 | 0.01 |  | 3 vs 4 <0.0001 |  | 3 vs 4 = 0.11 |
| **S-IgA** **level on day 42 (Raw data), day 42** | | | | | | | | | | | | | |
| 1 | TOMAVAC | 6 | 2.17 | 4.36 | 2.18 | 3.30 | 0.71 | 0.29 | 0.76 | F=58.14  P<0.001  Rsq=0.89 | 1 vs 2 <0.001 | KW=19.45  P=0.0002 | 1 vs 2 =0.0006 |
| 2 | Untransformed tomato | 6 | 0.19 | 0.45 | 0.26 | 0.32 | 0.10 | 0.04 | 0.72 |  | 1 vs 3 <0.001 |  | 1 vs 3 =0.0042 |
|  |  |  |  |  |  |  |  |  |  |  | 1 vs 4 <0.001 |  | 1 vs 4 =0.99 |
| 3 | Standard diet control | 6 | 0.25 | 0.53 | 0.27 | 0.38 | 0.10 | 0.04 | 0.62 |  | 2 vs 3 > 0.99 |  | 2 vs 3 >0.99 |
|  |  |  |  |  |  |  |  |  |  |  | 2 vs 4 =0.001 |  | 2 vs 4 = 0.07 |
| 4 | Astrazeneca | 6 | 1.04 | 2.22 | 1.18 | 1.48 | 0.53 | 0.22 | 0.03 |  | 3 vs 4 =0.002 |  | 3 vs 4 = 0.27 |
| **S-IgA** **level on day 56 (Raw data), day 56** | | | | | | | | | | | | | |
| 1 | TOMAVAC | 4 | 1.30 | 1.79 | 0.49 | 1.57 | 0.24 | 0.12 | 0.25 | F=56.27  P<0.001  Rsq=0.93 | 1 vs 2 <0.001 | KW=12.88  P<0.0001 | 1 vs 2 =0.03 |
| 2 | Untransformed tomato | 4 | 0.25 | 0.35 | 0.10 | 0.33 | 0.05 | 0.02 | 0.00 |  | 1 vs 3 <0.001 |  | 1 vs 3 =0.008 |
|  |  |  |  |  |  |  |  |  |  |  | 1 vs 4 <0.001 |  | 1 vs 4 >0.99 |
| 3 | Standard diet control | 4 | 0.11 | 0.55 | 0.43 | 0.31 | 0.18 | 0.09 | 0.93 |  | 2 vs 3 = 0.99 |  | 2 vs 3 >0.99 |
|  |  |  |  |  |  |  |  |  |  |  | 2 vs 4 =0.003 |  | 2 vs 4 = 0.71 |
| 4 | Astrazeneca | 4 | 0.76 | 0.91 | 0.15 | 0.83 | 0.06 | 0.03 | 0.99 |  | 3 vs 4 =0.003 |  | 3 vs 4 =0.26 |

**Note**: based on these analyses, the statistical significance levels were reported as * – ≤ 0.05, ** –≤ 0.01, and *** – ≤ 0.001 for ordinary one-way ANOVA analysis, and KW* – ≤ 0.05, KW**– ≤ 0.01, KW***– ≤ 0.001) for Kruskal Wallis (KW) one-way ANOVA. SD is the standard deviation; SEM is the standard error of the mean.

**Supplementary Table 9.** Statistical analysis of data from oral immunization of TOMAVAC in human consumption study.

| **No.** | **Groups** | **Size, n** | **Minimum** | **Maximum** | **Range** | **Mean** | **SD** | **SEM** | **Normality, Shapiro-Wilk test p-value (α = 0.05)** | **RM One-way ANOVA, summary** | **Friedman test** | **Adjusted p-values from Dunn’s multiple comparisons** |
| --- | --- | --- | --- | --- | --- | --- | --- | --- | --- | --- | --- | --- |
| **TOMAVAC human consumption (Raw data)** | | | | | | | | | | | | |
| 1. | Initial day | 7 | 61.1 | 440.4 | 379.3 | 312.3 | 169.4 | 64.01 | 0.009 | Not performed due to data normality issue | F = 9.0  P = 0.03 |  |
| 2. | 7^th^ day | 7 | 74.07 | 475.7 | 401.6 | 347.7 | 183.1 | 69.22 | 0.001 |  |  | 1 vs. 2 = 0.007 |
| 3. | 14^th^ day | 7 | 65.18 | 477.4 | 412.2 | 350.1 | 194.7 | 73.57 | <0.001 |  |  | 1 vs. 3 = 0.06 |
| 4. | 21^st^ day | 7 | 68.86 | 544.5 | 475.6 | 365.8 | 205.1 | 77.53 | 0.009 |  |  | 1 vs. 4 = 0.01 |
| **TOMAVAC human consumption (Log10 data)** | | | | | | | | | | | | |
| 1. | Initial day | 7 | 1.786 | 2.644 | 0.8578 | 2.39 | 0.3795 | 0.1435 | 0.003 | Not performed due to data normality issue | F = 9.0  P = 0.03 |  |
| 2. | 7^th^ day | 7 | 1.87 | 2.677 | 0.8077 | 2.442 | 0.3692 | 0.1395 | <0.001 |  |  | 1 vs. 2 = 0.007 |
| 3. | 14^th^ day | 7 | 1.814 | 2.679 | 0.8648 | 2.424 | 0.4144 | 0.1566 | <0.001 |  |  | 1 vs. 3 = 0.06 |
| 4. | 21^st^ day | 7 | 1.838 | 2.736 | 0.898 | 2.443 | 0.4124 | 0.1559 | 0.001 |  |  | 1 vs. 4 = 0.01 |
| **Human consumption control (Raw data)** | | | | | | | | | | | | |
| 1. | Initial day | 7 | 67.59 | 510.7 | 443.2 | 247.6 | 175.6 | 66.38 | 0.32 | F = 6.8  P = 0.003;  1 vs. 2 = 0.32  1 vs. 3 = 0.10  1 vs. 4 <0.001;  pairwise Dunnett’s comparisons | F = 19.29  P < 0.001 |  |
| 2. | 7^th^ day | 7 | 66.48 | 513.9 | 447.4 | 229 | 174.2 | 65.83 | 0.28 |  |  | 1 vs. 2 > 0.99 |
| 3. | 14^th^ day | 7 | 65.23 | 507.4 | 442.2 | 219.9 | 172.4 | 65.15 | 0.21 |  |  | 1 vs. 3 = 0.04 |
| 4. | 21^st^ day | 7 | 54.21 | 502 | 447.8 | 193.2 | 176.5 | 66.71 | 0.06 |  |  | 1 vs. 4 < 0.001 |
| **Human consumption control (Log10 data)** | | | | | | | | | | | | |
| 1. | Initial day | 7 | 1.83 | 2.708 | 0.8783 | 2.279 | 0.3582 | 0.1354 | 0.28 | F = 6.01  P = 0.005;  1 vs. 2 = 0.57  1 vs. 3 = 0.27  1 vs. 4 <0.002;  pairwise Dunnett’s comparisons | F = 19.29  P < 0.001 |  |
| 2. | 7^th^ day | 7 | 1.823 | 2.711 | 0.8882 | 2.236 | 0.3656 | 0.1382 | 0.32 |  |  | 1 vs. 2 > 0.99 |
| 3. | 14^th^ day | 7 | 1.814 | 2.705 | 0.8909 | 2.216 | 0.3658 | 0.1383 | 0.34 |  |  | 1 vs. 3 = 0.04 |
| 4. | 21^st^ day | 7 | 1.734 | 2.701 | 0.9667 | 2.121 | 0.4098 | 0.1549 | 0.10 |  |  | 1 vs. 4< 0.001 |

**Note**: the serum IgG levels (BAU/ml) in both experimental and control groups were statistically significant (a vs. b at p≤0.05) or nonsignificant (a vs. a), relative to initial day NAbs level, using Friedman’s ANOVA. SD is the standard deviation; SEM is the standard error of the mean.

**Supplementary Table 10.** Preliminary consumption study of “ТОМАVAC” on volunteered people.

| **№** | **Volunteer ID** | | **Age/Sex** | | **Initial serum IgG,**  **BAU/ml**  **(13.05.2022)** | **After TOMOVAC consumption, serum IgG level (difference), BAU/ml** | | | | ***Consumption time** | **Side effects observed** |
| --- | --- | --- | --- | --- | --- | --- | --- | --- | --- | --- | --- |
|  |  |  |  | |  | **On day 7**  **(20.05.2022)** | **On day 14**  **(27.05.2022)** | **On day 21**  **(3.06.2022)** | **Average difference** |  |  |
|  | | **Experimental group*** | | | | | | | | | |
| 1. | TVP-ID01 | | 43/M | | 416.11 | 453.70 (+37.59) | 472.04 (+55.93) | 438.71 (+22.60) | +38.71 | Before breakfast | Bloating |
| 2. | TVP-ID02 | | 47/M | | 337.04 | 444.07(+107.04) | 477.41 (+140.37) | 490.74 (+153.71) | +133.71 | Before breakfast | None |
| 3. | TVP-ID03 | | 40/M | | 431.48 | 475.70 (+44.22) | 467.04 (+35.56) | 458.89 (+27.41) | +35.73 | Before breakfast | Bloating |
| 4. | TVP-ID04 | | 30/M | | 77.96 | 86.11 (+8.15) | 66.11 (-11.85) | 69.82 (-8.14) | -3.95 | During the day | None |
| 5. | TVP-ID05 | | 31/M | | 61.10 | 74.07 (+12.97) | 65.18 (+4.08) | 68.86 (+7.75) | +8.27 | During the day | Bloating |
| 6. | TVP-ID07 | | 36/M | | 422.22 | 451.48 (+29.26) | 439.63 (+17.41) | 488.89 (+66.67) | +37.78 | Before breakfast | None |
| 7. | TVP-ID08 | | 30/M | | 440.37 | 448.89 (+8.52) | 462.96 (+22.59) | 544.45 (+104.08) | +45.06 | During the day | none |
|  |  | | | Average difference | | +35.39 | +37.73 | +53.44 | +42.19 | - | - |
|  | | **Control group*** | | | | | | | | | |
| 8. | TVP-ID11 | | 52/F | | 67.59 | 66.48 (-1.11) | 65.23 (-2.36) | 64.07 (-3.52) | -2.33 | - | - |
| 9. | TVP-ID22 | | 49/M | | 225.92 | 194.44 (-31.29) | 164.32 (-61.61) | 79.63 (-146.30) | -79.73 | - | - |
| 10. | TVP-ID33 | | 28/M | | 87.04 | 89.63 (+2.59) | 85.33 (-1.71) | 58.33 (-28.71) | -9.28 | - | - |
| 11. | TVP-ID44 | | 35/M | | 510.74 | 513.89 (+3.15) | 507.42 (-3.32) | 502.04 (-8.70) | -2.96 | - | - |
| 12. | TVP-ID55 | | 29/M | | 339.26 | 277.78 (-18.52) | 269.68 (-26.62) | 258.70 (37.60) | -27.58 | - | - |
| 13. | TVP-ID77 | | 27/M | | 95.19 | 70.00 (-25.19) | 68.72 (-26.47) | 54.21 (-40.98) | -30.88 | - | - |
| 14. | TVP-ID88 | | 50/M | | 407.40 | 390.74 (-16.66) | 378.54 (-54.86) | 335.19 (-72.22) | -47.91 | - | - |
|  |  | | | Average difference | | -12.43 | -25.28 | -48.29 | -28.67 | - | - |

**Note**: *- Participants consumed 50 grams (corresponding to 38.5 µg S1 protein) of tomatoes (TOMAVAC) from PCR-positive, single-copy transgenic tomato plant synthesizing S1 protein (0.77 µg/g) of SARS-CoV-2 during the first three days on an empty stomach 20-30 minutes before the breakfast or during the day before taking meal. The levels of neutralizing IgG were tested using the automatic chemiluminescence immunoassay analyzer MAGLUMI series, according to the manufacturer's instructions for human sera (Snibe Diagnostic, Pingshan, China). **The serum IgG levels (BAU/ml) in both experimental and control groups were statistically significant (p≤0.001) from ANOVA analysis using Dunnett's Multiple Comparison methods, where a Student's t-statistic for each experimental or control group was compared to the respective initial day serum IgG levels (see Fig. 4). M – Male; F- Female.

Supplemental References

1. Yang L, Pan A, Jia J, Ding J, Chen J, Cheng H, Zhang C, Zhang D. Validation of a tomato-specific gene, LAT52, used as an endogenous reference gene  in qualitative and real-time quantitative PCR detection of transgenic tomatoes. *J Agric Food Chem* (2005) 53:183–190. doi: 10.1021/jf0493730
2. Yi CX, Zhang J, Chan KM, Liu XK, Hong Y. Quantitative real-time PCR assay to detect transgene copy number in cotton  (*Gossypium hirsutum*). *Anal Biochem* (2008) 375:150–152. doi: 10.1016/j.ab.2007.11.022
3. Weng H, Pan A, Yang L, Zhang C, Liu Z, Zhang D. Estimating number of transgene copies in transgenic rapeseed by real-time PCR assay with HMG I/Y as an endogenous reference gene. *Plant Mol Biol Report* (2004) 22:289–300. doi: 10.1007/BF02773139
4. Ginzinger DG. Gene quantification using real-time quantitative PCR: an emerging technology hits the mainstream. *Exp Hematol* (2002) 30:503–512. doi: 10.1016/s0301-472x(02)00806-8
5. Suzuki Y, Makino A, Mae T. An efficient method for extraction of RNA from rice leaves at different ages using benzyl chloride. *J Exp Bot* (2001) 52:1575–1579. doi: 10.1093/jexbot/52.360.1575
6. Wu Y, Llewellyn DJ, Dennis ES. A quick and easy method for isolating good-quality RNA from cotton (Gossypium hirsutum L.) tissues. *Plant Mol Biol Report* (2002) 20:213–218. doi: 10.1007/BF02782456
7. Cronn RC, Small RL, Haselkorn T, Wendel JF. Rapid diversification of the cotton genus (Gossypium: Malvaceae) revealed by analysis of sixteen nuclear and chloroplast genes. *Am J Bot* (2002) 89:707–725. doi: 10.3732/ajb.89.4.707
8. Livak KJ, Schmittgen TD. Analysis of relative gene expression data using real-time quantitative PCR and the 2(-Delta Delta C(T)) Method. *Methods* (2001) 25:402–408. doi: 10.1006/meth.2001.1262
9. Burns P, Oddi S, Forzani L, et al. Variability in gut mucosal secretory IgA in mice along a working day. *BMC Res Notes* (2018) 11:98. doi: 10.1186/s13104-018-3213-0
10. Rodrigues AC, Cara DC, Fretez SH, Cunha FQ, Vieira EC, Nicoli JR, et al. *Saccharomyces boulardii* stimulates sIgA production and the phagocytic system of gnotobiotic mice. *J Appl Microbiol.* (2000) 89(3):404–14. doi: 10.1046/j.1365-2672.2000.01128.x
11. Menzel W, Jelkmann W, Maiß E. Detection of four apple viruses by multiplex RT-PCR assays with coamplification of plant mRNA as internal control. *J Virol Methods* (2002) 99:81–92. doi: 10.1016/S0166-0934(01)00381-0
