# Supplementary material for: Tomato-made edible COVID-19 vaccine TOMAVAC induces neutralizing IgGs in the blood sera of mice and humans
Source: Front Nutr. 2024 Jan 8;10:1275307. doi: 10.3389/fnut.2023.1275307 (PMC10800535; doi:10.3389/fnut.2023.1275307)
Supplement: Supplementary file 2 [file Data_Sheet_2.docx]

Supplementary Material

The authors have provided this trial protocol to give readers additional information about their work.

Supplement to: Buriev ZT, Shermatov SE, Usmonov DE, et al. Tomato-made edible COVID-19 vaccine TOMAVAC induces neutralizing IgGs blood serums of mice and humans. Journal, submitted.

**Table of content**

[1 1. PROTOCOL SUMMARY 5](#_Toc152787575)

[1.1 1.1. Synopsis 5](#_Toc152787576)

[1.1.1 Objectives, Estimands, and Endpoints 6](#_Toc152787577)

[1.1.2 Overall Design 7](#_Toc152787578)

[1.1.3 Number of Participants 7](#_Toc152787579)

[1.1.4 Intervention Groups and Duration 7](#_Toc152787580)

[1.1.5 Data Monitoring Committee or Other Independent Oversight Committee 7](#_Toc152787581)

[1.1.6 Statistical Methods 7](#_Toc152787582)

[1.2 1.2. Scheme 8](#_Toc152787583)

[1.3. Schedule of Activities 9](#_Toc152787584)

[1.3.1. Stage 1 Sentinel Cohorts 9](#_Toc152787585)

[1.3.2. Stage 1 Nonsentinel Cohorts and Stage 2 Cohorts 11](#_Toc152787586)

[2. INTRODUCTION 14](#_Toc152787587)

[2.1. Study Rationale 14](#_Toc152787588)

[2.2. Background 14](#_Toc152787589)

[2.2.1. Clinical Overview 14](#_Toc152787590)

[2.3. Benefit/Risk Assessment 14](#_Toc152787591)

[2.3.1. Risk Assessment 15](#_Toc152787592)

[2.3.2. Benefit Assessment 16](#_Toc152787593)

[2.3.3. Overall Benefit/Risk Conclusion 16](#_Toc152787594)

[3. OBJECTIVES, ESTIMANDS, AND ENDPOINTS 16](#_Toc152787595)

[4. STUDY DESIGN 17](#_Toc152787596)

[4.1 Overall Design 17](#_Toc152787597)

[4.1.1. Stage 1 17](#_Toc152787598)

[4.1.2. Stage 2 18](#_Toc152787599)

[4.2. Scientific Rationale for Study Design 18](#_Toc152787600)

[4.3. Justification for Dose 18](#_Toc152787601)

[4.4. End of Study Definition 18](#_Toc152787602)

[5. STUDY POPULATION 18](#_Toc152787603)

[5.1. Inclusion Criteria for Animals 18](#_Toc152787604)

[5.2. Exclusion Criteria for Animals 19](#_Toc152787605)

[5.3. Inclusion Criteria for Humans 19](#_Toc152787606)

[5.4. Exclusion Criteria for Humans 19](#_Toc152787607)

[5.5. Screen Failures 20](#_Toc152787608)

[5.6. Criteria for Temporarily Delaying Enrolment/Randomization/Study Intervention Administration 20](#_Toc152787609)

[6. STUDY INTERVENTION 21](#_Toc152787610)

[1.3 6.1. Study Intervention(s) Administered 21](#_Toc152787611)

[6.1.1. Administration 21](#_Toc152787612)

[6.2. Measures to Minimize Bias: Randomization and Blinding 21](#_Toc152787613)

[6.2.1. Allocation to Study Intervention 21](#_Toc152787614)

[6.2.2. Blinding of Site Personnel 21](#_Toc152787615)

[6.2.3. Blinding of the Center 21](#_Toc152787616)

[6.3. Study Intervention Compliance 22](#_Toc152787617)

[6.4. Concomitant Therapy 22](#_Toc152787618)

[6.4.1. Prohibited During the Study 22](#_Toc152787619)

[6.4.2. Permitted During the Study 22](#_Toc152787620)

[6.5. Dose Modification 22](#_Toc152787621)

[6.6. Intervention After the End of the Study 22](#_Toc152787622)

[7. DISCONTINUATION OF STUDY INTERVENTION AND PARTICIPANT DISCONTINUATION/WITHDRAWAL 22](#_Toc152787623)

[7.1. Discontinuation of Study Intervention 22](#_Toc152787624)

[7.2. Participant Discontinuation/Withdrawal from the Study 23](#_Toc152787625)

[7.2.1. Withdrawal of Consent 23](#_Toc152787626)

[7.3. Lost to Follow-up 23](#_Toc152787627)

[8. STUDY ASSESSMENTS AND PROCEDURES 24](#_Toc152787628)

[8.1. Efficacy and/or Immunogenicity Assessments in Animals 24](#_Toc152787629)

[8.2. Efficacy and/or Immunogenicity Assessments in Humans 24](#_Toc152787630)

[8.2.1. Biological Samples 25](#_Toc152787631)

[8.3. Safety Assessments 25](#_Toc152787632)

[8.3.1. Diary 25](#_Toc152787633)

[8.3.2. Stopping Rules 27](#_Toc152787634)

[8.3.3. Surveillance of Events That Could Represent Enhanced COVID-19 Disease 28](#_Toc152787635)

[8.4. Adverse Events and Serious Adverse Events 28](#_Toc152787636)

[8.4.1. Time Period and Frequency for Collecting AE and SAE Information 28](#_Toc152787637)

[8.4.2. Method of Detecting AEs and SAEs 29](#_Toc152787638)

[8.4.3. Follow-up of AEs and SAEs 29](#_Toc152787639)

[8.4.4. Regulatory Reporting Requirements for SAEs 29](#_Toc152787640)

[8.4.5. Medication Errors 29](#_Toc152787641)

[8.5. Treatment of Overdose 30](#_Toc152787642)

[8.6. Pharmacokinetics 30](#_Toc152787643)

[8.7. Pharmacodynamics 30](#_Toc152787644)

[8.8. Genetics 30](#_Toc152787645)

[8.9. Biomarkers 30](#_Toc152787646)

[8.10. Immunogenicity Assessments 30](#_Toc152787647)

[8.11. Health Economics 30](#_Toc152787648)

[8.12. Study Procedures 30](#_Toc152787649)

[8.12.1. Sentinel Cohorts for Humans 30](#_Toc152787650)

[8.12.2. Nonsentinel Cohorts for Humans 34](#_Toc152787651)

[8.12.3. Study procedure for Animal Sentinel Cohorts for TOMAVAC 38](#_Toc152787652)

[8.12.4. Study procedure for Animal Sentinel Cohorts for AstraZeneca 40](#_Toc152787653)

[8.12.5. Study Procedure for Animal Nonsentinel Cohorts 42](#_Toc152787654)

[8.13. Unscheduled Visit for Suspected Grade 4 Reaction 44](#_Toc152787655)

[8.14. COVID-19 Disease Surveillance (All Participants) 45](#_Toc152787656)

[8.14.1. Potential COVID-19 Illness Visit: (Optimally Within 3 Days After Potential COVID-19 Illness Onset) 45](#_Toc152787657)

[8.13.2. Potential COVID-19 Convalescent Visit: (28 to 35 Days After Potential COVID-19 Illness Visit) 45](#_Toc152787658)

[9. SUPPORTING DOCUMENTATION AND OPERATIONAL CONSIDERATIONS 47](#_Toc152787659)

[9.1. Appendix A: Regulatory, Ethical, and Study Oversight Considerations 47](#_Toc152787660)

[9.1.1. Regulatory and Ethical Considerations 47](#_Toc152787661)

[9.1.2. Informed Consent Process 47](#_Toc152787662)

[9.1.3. Data Protection 48](#_Toc152787663)

[9.1.4. Data Quality Assurance 48](#_Toc152787664)

[9.1.5. Source Documents 49](#_Toc152787665)

[9.1.6. Study and Site Start and Closure 49](#_Toc152787666)

[9.1.8. Center’s Qualified Medical Personnel 49](#_Toc152787667)

[9.2.1. Definition of AE 50](#_Toc152787668)

[9.2.2. Definition of SAE 50](#_Toc152787669)

[9.2.3. Recording/Reporting and Follow-up of AEs and/or SAEs 51](#_Toc152787670)

[9.2.4. Reporting of SAEs 53](#_Toc152787671)

[9.3. Appendix C: Abbreviations 54](#_Toc152787672)

# 1. PROTOCOL SUMMARY

## 1.1. Synopsis

**Short Title:** A Proof-of-Concept Animal and Human Study to Describe the Safety, Tolerability, Immunogenicity, and Potential Efficacy of Edible Vaccine Candidates Against COVID-19 in Mice and Healthy Adults

**Rationale**

Since the discovery of SARS-CoV-2 in 2019, the virus has swept the entire planet. On December 23, 2022, more than 652 million cases were registered, and the number of deaths is approaching 6,7 million. Vaccination is considered the most effective way to treat and prevent COVID-19.

According to WHO, as on November 22, 2022, 11 vaccines have been approved for unrestricted use and 9 for restricted use. Additionally, 175 candidate vaccines are in clinical trials, of which 11 are in stage 4 clinical trials, six are in phase 3 clinical trials, 9 are in stage 2 and 2/3 clinical trials, 45 are in stage 1 and 1/2 clinical trials, and 199 are in preclinical studies. Many commercially available vaccines are parenteral and administered intramuscularly to induce only systemic immunity against the infectious SARS-CoV-2.

There is a need to develop a new generation of edible COVID-19 vaccines that harness both mucosal and systemic immunity protecting against viral transmission and disease with the advantages of production, storage, and administration over conventional parenteral vaccination.

Center of Genomics and Bioinformatics of the Academy Sciences of Uzbekistan has developed an edible tomato vaccine candidate using *in fruit* transformation of tomato into which a genetic construct containing the subunit 1 gene of the coronavirus S protein was inserted. As a result, PCR-positive tomatoes (TOMAVAC) were obtained that stably express SARS-CoV-2 S1 protein in the T_1_ generation. The SARS-CoV-2 vaccine candidate TOMAVAC will be tested in this study.

This study is intended to investigate the safety, immunogenicity, and potential efficacy of this prophylactic TOMAVAC vaccine against COVID-19.

### Objectives, Estimands, and Endpoints

| **Objectives** | **Estimands** | **Endpoints** |
| --- | --- | --- |
| **Phase I** | | |
| **Primary:** | **Primary:** | **Primary:** |
| To describe the safety and tolerability profiles of the prophylactic TOMAVAC vaccine in animals after two doses | Among animals that received both doses of TOMAVAC vaccine, the percentage of animals that experienced:   - Local reactions up to 7 days after each dose - Systemic events up to 7 days after each dose - Adverse events (AEs) within one month of the last dose - Serious AEs (SAEs) within one month of the last dose | - Local reactions (erythema and allergic reactions) - Systemic events (fever, fatigue, headache, chills, vomiting, diarrhea) - AEs - SAEs |
| **Secondary:** | **Secondary:** | **Secondary:** |
| To describe the immune responses elicited by prophylactic TOMAVAC vaccines in mice after 1 and 2 doses | In animals complying with the key protocol criteria at the following time points after receipt of study intervention:  Sentinel Cohorts:  14th day after Dose 1;  14th, 28th and 42th days Dose 2  Nonsentinel Cohorts:  14th day after Dose 1;  14th, 28th and 42th days Dose 2 |  |
|  | - Geometric mean titers (GMTs) of NAbs and S-IgA at each time point - Geometric mean fold rise (GMFR) from before vaccination to each subsequent time point after vaccination - Neutralizing activity in a surrogate virus neutralization test (sVNT) |  |
| **Phase II** | | |
| **Primary:** | **Primary:** | **Primary:** |
| To describe the safety and tolerability profiles of the prophylactic TOMAVAC vaccine in healthy adult volunteers after one dose | Among animals that received both doses of TOMAVAC vaccine, the percentage of animals that experienced:   - Local reactions up to seven days after each dose - Systemic events up to seven days after each dose - Adverse events (AEs) within one month of the last dose - Serious AEs (SAEs) within one month of the last dose | - Local reactions (redness and swelling) - Systemic events (fever, fatigue, headache, chills, vomiting, and diarrhea) - AEs - SAEs |
| **Secondary:** | **Secondary:** | **Secondary:** |
| Describing the immune responses elicited by prophylactic TOMAVAC vaccines in healthy adult volunteers after a single dose | In healthy adult volunteers complying with the key protocol criteria at the following time points after receipt of study intervention:  Sentinel Cohorts:  7^th^, 14^th^, 21^th^ and 28th days Dose 1  Nonsentinel Cohorts:  7^th^, 14^th^, 21^th^ and 28th days Dose 1 |  |
|  | - Geometric mean titers (GMTs) at each time point - Geometric mean fold rise (GMFR) from before vaccination to each subsequent time point after vaccination - Neutralizing activity in a surrogate virus neutralization test (sVNT) |  |

### Overall Design

This is a randomized, placebo-controlled study in animals and healthy adult volunteers.

The study will evaluate the safety, tolerability, immunogenicity, and potential efficacy of edible vaccine candidates against COVID-19:

As a 2-dose (separated by 14 days) or single-dose schedule

In 2 groups, including animals (mice) and healthy adult volunteers 30-52 years old.

The research consists of three stages. Step 1: evaluation of safety, tolerability, immunogenicity, and potential efficacy in animals; Step 2: Evaluation of safety, tolerability, immunogenicity, and potential efficacy in a limited group of volunteers. These stages and the transition between them are described in detail in the diagram.

### Number of Participants

Each group in Stage 1 will comprise 24 mice (with an equal number of males and females), and three potential groups are foreseen; (1 - TOMAVAC group, force-fed with 1 ml homogenate (equivalent to ≈0.77 µg S1 protein) from a single copy transgenic tomato event 4 before morning standard diet feeding; 2 - untransformed tomato group, force-fed 1 ml homogenate of untransformed tomato from cv. Bella Rossa® before morning standard diet feeding; 3 - control group, fed only with a standard diet; and 4 – a positive control, parenterally vaccinated with AstraZeneca vaccine (equivalent to ≈16×10^6^ infectious units); if all groups are fully enrolled, this corresponds to a total of 96 animals.

The group in Stage 2 will comprise 14 participants (seven receiving an active vaccine and seven receiving a placebo).

### Intervention Groups and Duration

The study may evaluate single-dose and 2-dose schedules of edible vaccine candidates for active immunization against COVID-19. Animals are expected to participate for up to a maximum of approximately two months. Participants are expected to participate for up to a maximum of approximately 1,5 months.

### Data Monitoring Committee or Other Independent Oversight Committee

The study will utilize an IRC, an internal committee of the Center of Genomics and Bioinformatics of the Academy of Sciences of Uzbekistan that will review data to allow dose escalation or changes to the continuation of specific groups.

An external data monitoring committee (DMC) will be formed to review cumulative unblinded data throughout the study.

### Statistical Methods

The study sample size for the first two stages of the study is not based on any statistical hypothesis testing.

For each vaccine group, the primary safety objective will be evaluated by descriptive summary statistics for local reactions, systemic events, and AEs and SAEs. A 3-tier approach will be used to summarise AEs.

The secondary immunogenicity objectives will be evaluated descriptively by GMT, GMFR, and the 95% confidence intervals (CIs) for SARS-CoV-2-specific surrogate virus-neutralizing titers.

## 1.2. Scheme

Proof-of-concept animal study (safety & immunogenicity)


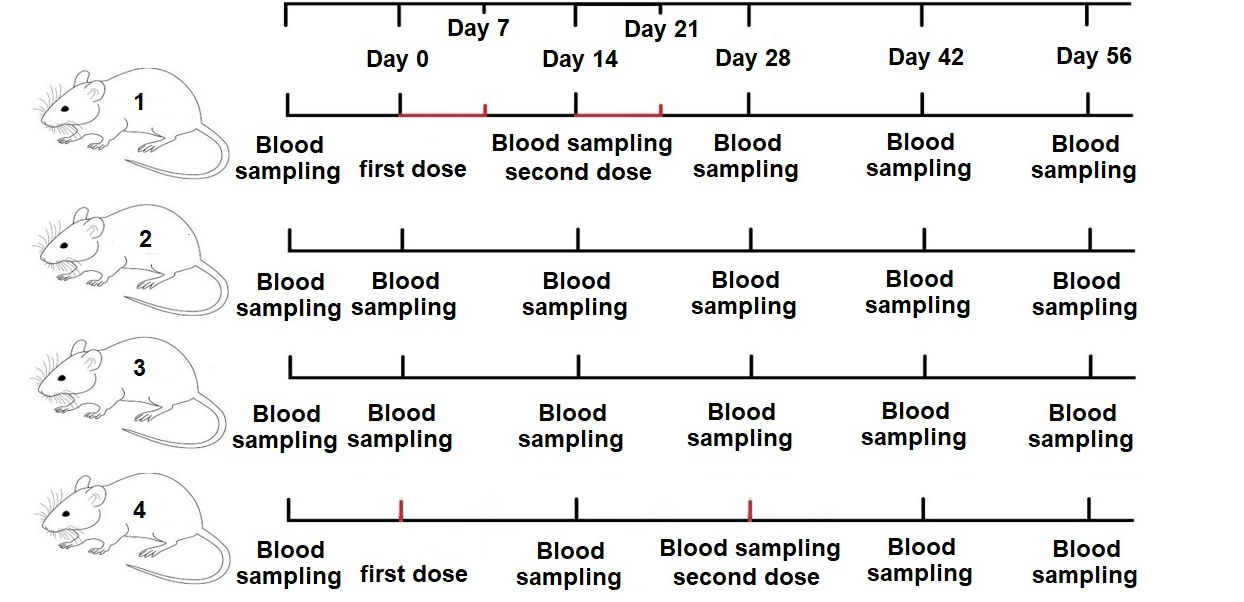


1–TOMAVAC-fed; 2–untransformed tomato-fed; 3–standar diet-fed; 4–AstraZeneca-injected.

Proof-of-concept human study (safety & immunogenicity)


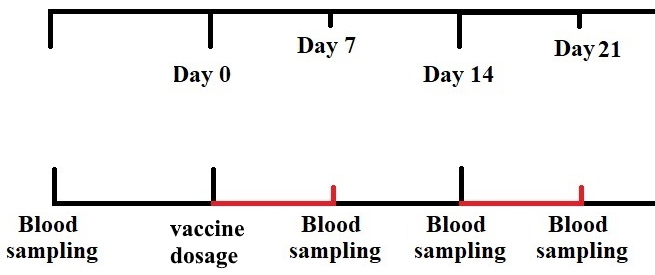


## 1.3. Schedule of Activities

The SoA table provides an overview of the protocol visits and procedures. Refer to the STUDY ASSESSMENTS AND PROCEDURES section of the protocol for detailed information on each procedure and assessment required for compliance with the protocol.

The investigator may schedule visits (unplanned visits) in addition to those listed in the SoA table to conduct evaluations or assessments required to protect the well-being of the participants.

### 1.3.1. Stage 1 Sentinel Cohorts

**For animals**

An unscheduled inspection of animals is carried out in case of the death of animals in any of the three groups.

| **Visit Number** | **Screening** | **1** | **2** | **3** | **4** | **5** | **6** | **7** | **8** | **9** | **10** | **Unplanned** |
| --- | --- | --- | --- | --- | --- | --- | --- | --- | --- | --- | --- | --- |
| **Visit Description** | **Screening** | **Vac 1** | **Next Day Follow-up Vac 1** | **1-Week Follow-up Vac 1** | **2-Week Follow-up Vac 1** | **Vac 2** | **1-Week Follow-up Vac 2** | **2-Week Follow-up Vac 2** | **3-Week Follow-up Vac 2** | **4-Week Follow-up Vac 2** | **6-Week Follow-up Vac 2** | **In case of the death of animals in any of the three groups** |
| **Visit Window (Days)** | **0 to 14 Days Before Vac 1** | **Day 1** | **1 Day After Vac 1** | **7 Days After Vac 1** | **14 Day After Vac 1** | **14 Day After Vac 1** | **7 Day After Vac 2** | **14 Day After Vac 2** | **21 Day After Vac 2** | **28 Day After Vac 2** | **42 Day After Vac 2** | **Optimally Within 3 Days After the death of animals in any of the three groups** |
| Assign animal number | X |  |  |  |  |  |  |  |  |  |  |  |
| Obtain details of medications currently taken. | X |  |  |  |  |  |  |  |  |  |  |  |
| Perform physical examination | X | X | X | X | X | X | X | X | X | X | X | X |
| Serological test for prior COVID-19 infection | X |  |  |  |  |  |  |  |  |  |  |  |
| Collect blood and intestinal lavage fluid samples for immunogenicity assessment. |  |  |  |  | X |  |  | X |  | X | X |  |
| Assess acute reactions for at least 30 minutes after study intervention administratio[n](#bookmark14). |  | X |  |  |  | X |  |  |  |  |  |  |

**For Volunteers**

An unplanned potential COVID-19 illness visits and unexpected potential COVID-19 convalescent visit are required anytime between Visit 1 (Vaccination 1) and Visit 8 (6-week follow-up visit) that COVID-19 is suspected.

| **Visit Number** | **Screening** | **1** | **2** | **3** | **4** | **5** | **6** | **7** | **8** | **Unplanned** | **Unplanned** |
| --- | --- | --- | --- | --- | --- | --- | --- | --- | --- | --- | --- |
| **Visit Description** | **Screening** | **Vac 1** | **Next Day Follow-up Vac 1** | **1-Week Follow-up Vac 1** | **2-Week Follow-up Vac 1** | **3-Week Follow-up Vac 2** | **4-Week Follow-up Vac 2** | **5-Week Follow-up Vac 2** | **6-Week Follow-up Vac 2** | **Potential COVID-19 Illness Telehealth Visit** | **Potential COVID-19 Convalescent Visit** |
| **Visit Window (Days)** | **0 to 14 Days Before Vac 1** | **Day 1** | **1 Day After Vac 1** | **7 Days After Vac 1** | **14 Day After Vac 1** | **7 Day After Vac 2** | **14 Day After Vac 2** | **21 Day After Vac 2** | **28 Day After Vac 2** | **Optimally Within 3 Days After Potential COVID-19 Illness Onset** | **28 to 35 Days After Potential COVID-19 Illness Visit** |
| Obtain informed consent | X |  |  |  |  |  |  |  |  |  |  |
| Assign participant number | X |  |  |  |  |  |  |  |  |  |  |
| Obtain demography and medical history data. | X |  |  |  |  |  |  |  |  |  |  |
| Obtain details of medications currently taken. | X |  |  |  |  |  |  |  |  |  |  |
| Perform a physical examination. | X | X | X | X | X | X | X | X | X | X | X |
| Serological test for prior COVID-19 infection | X |  |  |  |  |  |  |  |  |  |  |
| Collect blood samples for immunogenicity assessment. |  |  |  | X | X | X |  |  |  |  |  |
| Assess acute reactions for at least 30 minutes after study intervention administratio[n](#bookmark14). |  | X |  |  |  | X |  |  |  |  |  |
| Provide participants with a 7-day e-diary, thermometer, and measuring device. |  | X |  |  |  |  |  |  |  |  |  |
| Review e-diary data (daily review is optimal during the active diary period) |  |  | X | X | X | X | X | X | X |  |  |
| Collect AEs and SAEs as appropriate. |  | X | X | X | X | X | X | X | X | X | X |

### 1.3.2. Stage 1 Nonsentinel Cohorts and Stage 2 Cohorts

**For animals**

An unscheduled inspection of animals is carried out in case of the death of animals in any of the three groups.

| **Visit Number** | **Screening** | **1** | **2** | **3** | **4** | **5** | **6** | **7** | **8** | **9** | **10** | **Unplanned** |
| --- | --- | --- | --- | --- | --- | --- | --- | --- | --- | --- | --- | --- |
| **Visit Description** | **Screening** | **Vac 1** | **Next Day Follow-up Vac 1** | **1-Week Follow-up Vac 1** | **2-Week Follow-up Vac 1** | **Vac 2** | **1-Week Follow-up Vac 2** | **2-Week Follow-up Vac 2** | **3-Week Follow-up Vac 2** | **4-Week Follow-up Vac 2** | **6-Week Follow-up Vac 2** | **In case of the death of animals in any of the three groups** |
| **Visit Window (Days)** | **0 to 14 Days Before Vac 1** | **Day 1** | **1 Day After Vac 1** | **7 Days After Vac 1** | **14 Day After Vac 1** | **14 Day After Vac 1** | **7 Day After Vac 2** | **14 Day After Vac 2** | **21 Day After Vac 2** | **28 Day After Vac 2** | **42 Day After Vac 2** | **Optimally Within 3 Days After the death of animals in any of the three groups** |
| Assign animal number | X |  |  |  |  |  |  |  |  |  |  |  |
| Obtain details of medications currently taken. | X |  |  |  |  |  |  |  |  |  |  |  |
| Perform a physical examination. | X | X | X | X | X | X | X | X | X | X | X | X |
| Serological test for prior COVID-19 infection | X |  |  |  |  |  |  |  |  |  |  |  |
| Collect blood and intestinal lavage fluid samples for immunogenicity assessment. |  |  |  |  | X |  |  | X |  | X | X |  |
| Assess acute reactions for at least 30 minutes after study intervention administratio[n](#bookmark14). |  | X |  |  |  | X |  |  |  |  |  |  |

**For Volunteers**

An unplanned potential COVID-19 illness visits and unexpected potential COVID-19 convalescent visit are required anytime between Visit 1 (Vaccination 1) and Visit 8 (6-week follow-up visit) that COVID-19 is suspected.

| **Visit Number** | **Screening** | **1** | **2** | **3** | **4** | **5** | **6** | **7** | **8** | **Unplanned** | **Unplanned** |
| --- | --- | --- | --- | --- | --- | --- | --- | --- | --- | --- | --- |
| **Visit Description** | **Screening** | **Vac 1** | **Next Day Follow-up Vac 1** | **1-Week Follow-up Vac 1** | **2-Week Follow-up Vac 1** | **3-Week Follow-up Vac 2** | **4-Week Follow-up Vac 2** | **5-Week Follow-up Vac 2** | **6-Week Follow-up Vac 2** | **Potential COVID-19 Illness Telehealth Visit** | **Potential COVID-19 Convalescent Visit** |
| **Visit Window (Days)** | **0 to 14 Days Before Vac 1** | **Day 1** | **1 Day After Vac 1** | **7 Days After Vac 1** | **14 Day After Vac 1** | **7 Day After Vac 2** | **14 Day After Vac 2** | **21 Day After Vac 2** | **28 Day After Vac 2** | **Optimally Within 3 Days After Potential COVID-19 Illness Onset** | **28 to 35 Days After Potential COVID-19 Illness Visit** |
| Obtain informed consent | X |  |  |  |  |  |  |  |  |  |  |
| Assign participant number | X |  |  |  |  |  |  |  |  |  |  |
| Obtain demography and medical history data. | X |  |  |  |  |  |  |  |  |  |  |
| Obtain details of medications currently taken. | X |  |  |  |  |  |  |  |  |  |  |
| Perform a physical examination. | X | X | X | X | X | X | X | X | X | X | X |
| Serological test for prior COVID-19 infection | X |  |  |  |  |  |  |  |  |  |  |
| Collect blood samples for immunogenicity assessment. |  |  |  | X | X | X |  |  |  |  |  |
| Assess acute reactions for at least 30 minutes after study intervention administratio[n](#bookmark14). |  | X |  |  |  | X |  |  |  |  |  |
| Provide participants with a 7-day e-diary, thermometer, and measuring device. |  | X |  |  |  |  |  |  |  |  |  |
| Review e-diary data (daily review is optimal during the active diary period) |  |  | X | X | X | X | X | X | X |  |  |
| Collect AEs and SAEs as appropriate. |  | X | X | X | X | X | X | X | X | X | X |

# 2. INTRODUCTION

The TOMAVAC edible COVID-19 vaccine is currently being investigated to prevent COVID-19 in mice and healthy adults.

## 2.1. Study Rationale

The study aims to carry out a proof-of-concept animal and human study and describe the safety, tolerability, immunogenicity, and potential efficacy of TOMAVAC edible COVID-19 vaccine candidates against COVID-19 in mice and healthy adults. There are currently no edible vaccines to prevent infection with SARS-CoV-2. Given the global crisis of COVID-19 and the fast expansion of the disease, developing an effective edible vaccine is essential.

## 2.2. Background

Since the discovery of severe acute respiratory syndrome coronavirus 2 (SARS-CoV-2) in 2019, the virus has spread around the globe. According to Johns Hopkins University, as of December 23, 2022, more than 650 million cases were registered, and the number of deaths is approaching 6,7 million (1). Vaccination is considered the most effective way to treat and prevent COVID-19 (2-5). According to WHO, as on November 22, 2022, 11 vaccines are approved for unrestricted use, 9 for restricted use, and 175 candidate vaccines are in clinical trials, of which 11 are in stage 4 clinical trials, six are in phase 3 clinical trials, 9 in stage 2 and 2/3 clinical trials, 45 in stage 1 and 1/2 clinical trials, and 199 in preclinical studies (6,7). All possible platforms are used to develop vaccines against COVID-19; however, vaccines based on DNA, mRNA, and protein subunits (including receptor binding domain (RBD) of S1 protein) are most often used (2,6). Many of these commercially available vaccines are parenteral and administered intramuscularly to induce only systemic immunity against the infectious SARS-CoV-2 (8,9).

However, coronavirus spreads via air droplets among people, where commonly used parenteral vaccines are less effective for the protection of viral transmission (10), enabling its successful replication in the epithelial cells of the upper respiratory tract. Because of ineffective primary-entrance cell viral neutralization by circulating IgG of the systemic immune system, there is a risk of developing and spreading new SARS-CoV-2 variants (10). Moreover, the production of such injection-type vaccines is resource-intensive and ecologically unfriendly (5,13), which require special conditions for transportation, storage, and use (5,8,12-14).

Alternatively, plant-based edible COVID-19 vaccines can be developed and used^5,12^. Using plants as a "factory" for vaccine production also provides an opportunity for targeted folding and post-translational modifications of proteins to synthesize antigens with the desired properties (5). Importantly, oral, intranasal, or edible vaccines activate both mucosal and systemic immune responses, providing adequate and long-term protection against viral transmission, disease progression, and antiviral prophylaxis (10,11).

Edible vaccines, however, require no heat treatment, or the expressed antigenic molecule should be heat-stable and non-allergenic (8,12,14). It is essential to standardize sufficient and stable expression of targeted antigens in plant cells/tissues (12), as well as determine the oral dose amount (12,13), which is adequate to induce an immune response without or with limited immune tolerance reaction (8,11-14). To date, limited attempts have been made toward developing an edible COVID-19 vaccine^5^, although there are many plant-carrier-based examples of edible vaccines for various infectious diseases (8,9,12,14).

Center of Genomics and Bioinformatics of the Academy Sciences of Uzbekistan has developed an edible tomato vaccine candidate using *in fruit* transformation of tomato into which a genetic construct containing the subunit 1 gene of the coronavirus S protein. As a result, PCR-positive tomatoes (TOMAVAC) were obtained that stably express SARS-CoV-2 S1 protein in the T_1_ generation. The SARS-CoV-2 vaccine candidate TOMAVAC will be tested in this study.

This study is intended to investigate the safety, immunogenicity, and potential efficacy of this prophylactic TOMAVAC vaccine against COVID-19.

### 2.2.1. Clinical Overview

Edible COVID-19 vaccines have not been administered to animals and humans before; thus, there is no previous clinical data on these specific vaccines. However, given clinical data from other similarly edible vaccines (12,13) the TOMAVAC edible vaccine is expected to have a favorable safety profile with mild, localized, and transient effects.

## 2.3. Benefit/Risk Assessment

There is an ongoing global pandemic, COVID-19. While there are currently no data available from clinical trials on the use of edible vaccines in humans, available nonclinical data with these vaccines, and data from nonclinical studies and clinical trials with the same vaccines, support a favorable risk/benefit profile. Anticipated AEs after vaccination is expected to be manageable using the routine symptom-driven standard of care as determined by the investigators. As a result, the profile of these vaccine candidates supports the initiation of this study.

### 2.3.1. Risk Assessment

| **Potential Risk of Clinical Significance** | **Summary of Data/Rationale for Risk** | **Mitigation Strategy** |
| --- | --- | --- |
| **Study Intervention: TOMAVAC Edible COVID-19 Vaccine** | | |
| Potential for local reactions (erythema and allergic reactions) and systemic events (fever, fatigue, headache, chills, vomiting, diarrhea) following vaccination. | These are common adverse reactions seen with other vaccines, as noted in the FDA Center for Biologics Evaluation and Research (CBER) guidelines on toxicity grading scales for healthy adult volunteers enrolled in preventive vaccine clinical trials^15^. | The study design includes the use of sentinel cohorts and dose escalation to closely monitor and limit the enrolment rate to ensure participant safety. The study employs the use of an e-diary to monitor local reactions and systemic events in real time. Stopping rules are also in place for sentinel cohorts. The first five sentinel-cohort participants in each group will be observed for 4 hours after vaccination to assess any immediate AEs. |
| Unknown AEs and laboratory abnormalities with a novel vaccine. | This study is one of the first two parallel-running studies with the TOMAVAC edible vaccine candidate, and as such, there is no clinical data available for this vaccine. | The study design includes the use of sentinel cohorts and dose escalation to closely monitor and limit the enrolment rate to ensure participant safety. An IRC and DMC will also review safety data throughout the study. Stopping rules are also in place for sentinel cohorts. The first five sentinel cohort participants in each group will be observed for four hours after vaccination to assess any immediate AEs. |
| Potential for COVID-19 disease enhancement. | Disease enhancement has been seen following vaccination with the respiratory syncytial virus (RSV), feline coronavirus, and Dengue virus vaccines. | The study excludes participants with likely previous or current COVID-19. All participants are followed for SARS-CoV-2 antigen-specific antibody and SARS-CoV-2-specific WT serum neutralizing titers, and COVID-19 illness, including markers of severity. |
| **Study Procedures** | | |
| Participants will be required to attend healthcare facilities during the global SARS-CoV-2 pandemic. | Without appropriate social distancing and PPE, there is a potential for increased exposure to SARS-CoV-2. | Center of Genomics and Bioinformatics of the Academy of Sciences of Uzbekistan will work with sites to ensure an appropriate COVID-19 prevention strategy. |
| Venepuncture will be performed during the study. | There is a risk of bleeding, bruising, hematoma formation, and infection at the venepuncture site. | Only appropriately qualified personnel would obtain the blood draw. |

### 2.3.2. Benefit Assessment

Benefits to individual participants may include:

- Receipt of a potentially efficacious COVID-19 vaccine during a global pandemic
- Access to COVID-19 diagnostic and antibody testing
- Contributing to research to help others in a time of the global pandemic

### 2.3.3. Overall Benefit/Risk Conclusion

Taking into account the measures taken to minimize risk to participants participating in this study, the potential risks identified in association with TOMAVAC edible COVID-19 vaccine are justified by the anticipated benefits that may be afforded to healthy participants.

# 3. OBJECTIVES, ESTIMANDS, AND ENDPOINTS

| **Objectives** | **Estimands** | **Endpoints** |
| --- | --- | --- |
| **Phase I** | | |
| **Primary:** | **Primary:** | **Primary:** |
| To describe the safety and tolerability profiles of the prophylactic TOMAVAC vaccine in animals after two doses | Among animals that received both doses of TOMAVAC vaccine, the percentage of animals that experienced:   - Local reactions up to seven days after each dose - Systemic events up to seven days after each two doses - Adverse events (AEs) within one month of the last dose - Serious AEs (SAEs) within one month of the last dose | - Local reactions (erythema and allergic reactions) - Systemic events (fever, fatigue, headache, chills, vomiting, diarrhea) - AEs - SAEs |
| **Secondary:** | **Secondary:** | **Secondary:** |
| To describe the immune responses elicited by prophylactic TOMAVAC vaccines in mice after the first and second doses | In animals complying with the key protocol criteria at the following time points after receipt of study intervention:  Sentinel Cohorts:  14th day after Dose 1;  14th, 28th and 42th days Dose 2  Non-sentinel Cohorts:  14th day after Dose 1;  14th, 28th and 42th days Dose 2 |  |
|  | - Geometric mean titers (GMTs) for NAbs and S-IgA at each time point - Geometric mean fold rise (GMFR) from before vaccination to each subsequent time point after vaccination - Neutralizing activity in a surrogate virus neutralization test (sVNT) |  |
| **Phase II** | | |
| **Primary:** | **Primary:** | **Primary:** |
| To describe the safety and tolerability profiles of the prophylactic TOMAVAC vaccine in healthy adult volunteers after the first dose | Among animals that received both doses of TOMAVAC vaccine, the percentage of animals that experienced:   - Local reactions up to seven days after each dose - Systemic events up to seven days after each dose - Adverse events (AEs) within one month of the last dose - Serious AEs (SAEs) within one month of the last dose | - Local reactions redness and swelling) - Systemic events (fever, fatigue, headache, chills, vomiting, and diarrhea) - AEs - SAEs |
| **Secondary:** | **Secondary:** | **Secondary:** |
| To describe the immune responses elicited by prophylactic TOMAVAC vaccines in healthy adult volunteers after the first dose | In healthy adult volunteers complying with the key protocol criteria at the following time points after receipt of study intervention:  Sentinel Cohorts:  7th, 14th and 28th days Dose 1  Non-sentinel Cohorts:  7th, 14th and 28th days Dose 1 |  |
|  | - Geometric mean titers (GMTs) at each time point - Geometric mean fold rise (GMFR) from before vaccination to each subsequent time point after vaccination - Neutralizing activity in a surrogate virus neutralization test (sVNT) |  |

# 4. STUDY DESIGN

## 4.1 Overall Design

This is a randomized, placebo-controlled study in animals and healthy adult volunteers.

The study will evaluate the safety, tolerability, immunogenicity, and potential efficacy of edible vaccine candidates against COVID-19:

As a 2-dose (separated by 14 days) or single-dose schedule.

In 2 groups, including animals (mice) and healthy adult volunteers 30-52 years old).

The research consists of 2 stages. Step 1: evaluation of safety, tolerability, immunogenicity, and potential efficacy in animals; Step 2: Evaluation of safety, tolerability, immunogenicity, and potential efficacy in a limited group of volunteers. These stages and the transition between them are described in detail in the diagram.

### 4.1.1. Stage 1

Each group in Stage 1 will comprise 24 mice (with an equal number of males and females). All animals were kept on a standard diet that included 4g of cheese, 4g of carrot, 5g of bread, 1mL of milk, 0.1g of salt, 1g of beet, 2g of potato, 1g of oatmeal, 2mL of vitamin-mineral supplements, 19g of special feed, 2g of dog food. The daily feeding was between 7-8 am and 4-5 pm.

After initial verification of the non-infected status of the mice by sacrificing three mice for serum neutralizing antibodies and secretory immunoglobulin A analysis, 96 healthy mice 20-day-old outbred ICR (CD-1; n = 96) were divided into three equal groups. Groups were 1) TOMAVAC group, force-fed with 1 ml homogenate (equal to ≈0.77 µg S1 protein) from a single copy transgenic tomato event 4 before morning standard diet feeding; 2) untransformed tomato group, force-fed 1 ml homogenate of untransformed tomato from cv. Bella Rossa® before morning standard diet feeding; 3) control group, fed only with a standard diet; and 4) parenterally vaccinated with AstraZeneca vaccine as a positive control. A two-dose (days 1-7 and days 14-21), seven days-spaced force-feeding immunizations were carried out using a syringe.

Randomly chosen 24 mice to receive the TOMAVAC edible vaccine will constitute the control cohort, to which the following apply:

• Additional safety assessments (see Section 8.2)

• Controlled enrolment

• The animals must be observed by staff for at least 4 hours after vaccination for any acute reactions

• Application of stopping rules

• IRC review of safety data to determine escalation to the next stage

#### 4.1.1.1. Preparation of the working solution of AstraZeneca vaccine

10 µl of the original AstraZeneca vaccine were diluted to 1000 µl by sterile normal saline. Then the obtained solution was diluted to the final concentration of 16×10^6^ infectious units by sterile normal saline.

### 4.1.2. Stage 2

In Stage 2, a volunteer group of 14 healthy adults 30-52 years of age has given interest and written consent to participate in human consumption, a proof-of-concept experiment. After the ethical committee approved the study, volunteers were asked to be randomly grouped into two groups: 1) invited to consume TOMAVAC (n=7) before dining and 2) consuming non-transgenic tomatoes before dining (n=7). Both groups were instructed not to have any other vaccination during the experiments. All procedures were explained clearly to all participants, and their approval of using collected data for “research purposes” was obtained. Participants were informed about blood test results but de-identified for reporting purposes.

Briefly, the experimental group was provided and asked to take 50 g (equal to ≈38.5 µg S1 protein) of TOMAVAC on an empty stomach once a day for three consecutive days, 20-30 minutes before dining. Blood samples were taken from all participants before the human consumption experiment and on days 7, 14, and 21.

The 14 participants randomly divided into each group will comprise a sentinel cohort, to which the following apply:

- Additional safety assessments
- Controlled enrolment
- Application of stopping rules
- IRC review of safety data to determine escalation to the next dose level

## 4.2. Scientific Rationale for Study Design

Additional surveillance for COVID-19 will be conducted as part of the study, given the potential risk of disease enhancement. If the participant experiences respiratory symptoms or a COVID-19 illness, subsequent convalescent visits will occur. As part of these visits, samples (blood) will be taken for antigen and antibody assessment and recording of COVID-19-related clinical and laboratory information (including local diagnosis).

Human reproductive safety data are not available for plant-based edible COVID-19 vaccines, but there is no suspicion of human teratogenicity based on the intended mechanism of action of the compound.

## 4.3. Justification for Dose

Because of the requirement for a rapid response to the newly emerged COVID-19 pandemic, sufficient data are not currently available to experimentally validate the dose selection and initial starting dose. Therefore, for TOMAVAC, the following dose amount is planned to be used for mice feeding: ≈5.4 µg during seven days being ≈0.77 µg S1 protein daily feeding repeated for the second course of seven-day immunization. The total antigen amount immunized should be ≈10.8 µg. For human consumption, ≈38.5 µg S1 protein daily amount, totaling ≈116 µg antigen dose for a course of three days consumption, was planned.

For the AstraZeneca vaccine (as a positive control), 75 µl of working solution per is planned to be used for mice i/m injection (≈16×10^6^ infectious units per dose).

## 4.4. End of Study Definition

A participant is considered to have completed the study if he/she has completed all phases of the study, including the last visit.

The end of the study is defined as the date of the last visit of the last participant.

# 5. STUDY POPULATION

This study can fulfill its objectives only if appropriate animals and participants are enrolled. The following eligibility criteria are designed to select animals and participants for whom participation in the study is considered appropriate. All relevant medical and nonmedical conditions should be taken into consideration when deciding whether a particular participant is suitable for this protocol.

Prospective approval of protocol deviations to recruitment and enrolment criteria, also known as protocol waivers or exemptions, is not permitted.

## 5.1. Inclusion Criteria for Animals

Animals are eligible to be included in the study only if all of the following criteria apply:

1. 20-day-old outbred mice ICR (CD1)

2. equal number of males and females

3. absence of receptor binding domain-specific neutralizing antibodies in blood serum.

## 5.2. Exclusion Criteria for Animals

Animals are excluded from the study if any of the following criteria apply:

1. age over 20 days

2. presence of receptor binding domain-specific neutralizing antibodies in blood serum and secretory immunoglobulin A in intestinal lavage fluid.

## 5.3. Inclusion Criteria for Humans

Participants are eligible to be included in the study only if all of the following criteria apply:

Age and Sex:

1. Male or female participants between the ages of 25 and 50 years, inclusive, at randomization.

Type of Participant and Disease Characteristics:

2. Participants who are willing and able to comply with all scheduled visits, vaccination plans, laboratory tests, lifestyle considerations, and other study procedures.

3. Healthy participants who are determined by medical history, physical examination, and clinical judgment of the investigator are eligible for inclusion in the study.

Informed Consent:

4. Capable of giving personal signed informed consent, which includes compliance with the requirements and restrictions listed in the ICD and this protocol.

## 5.4. Exclusion Criteria for Humans

Participants are excluded from the study if any of the following criteria apply:

Medical Conditions:

1. Psychiatric condition including recent (within the past year) or active suicidal ideation/behavior or laboratory abnormality that may increase the risk of study participation or, in the investigator’s judgment, make the participant inappropriate for the study.

2. Known infection with human immunodeficiency virus (HIV), hepatitis C virus (HCV), or hepatitis B virus (HBV).

3. History of severe adverse reaction associated with a vaccine and/or severe allergic reaction (e.g., anaphylaxis) to any component of the study intervention(s).

4. Receipt of medications intended to prevent COVID-19.

5. Previous clinical or microbiological diagnosis of COVID-19.

6. Individuals at high risk for severe COVID-19, including those with any of the following risk factors:

• Hypertension

• Diabetes mellitus

• Chronic pulmonary disease

• Asthma

• Current vaping or smoking

• History of chronic smoking within the prior year

• BMI >30 kg/m^2^

• Anticipating the need for immunosuppressive treatment within the next six months

7. Individuals currently working in occupations with a high risk of exposure to SARS-CoV-2 (e.g., healthcare workers and emergency response personnel).

8. Immunocompromised individuals with known or suspected immunodeficiency, as determined by history and/or laboratory/physical examination.

9. Individuals with a history of autoimmune disease or an active autoimmune disease requiring therapeutic intervention, including but not limited to systemic or cutaneous lupus erythematosus, autoimmune arthritis/rheumatoid arthritis, Guillain-Barre syndrome, multiple sclerosis, Sjogren’s (SHOW-grins) syndrome, idiopathic thrombocytopenia purpura, glomerulonephritis, autoimmune thyroiditis, giant cell arteritis (temporal arteritis), psoriasis, and insulin-dependent diabetes mellitus (type 1).

10. Women who are pregnant or breastfeeding.

Prior/Concomitant Therapy:

11. Previous vaccination with any coronavirus vaccine.

12. Individuals who receive treatment with immunosuppressive therapy, including cytotoxic agents or systemic corticosteroids, e.g., for cancer or an autoimmune disease, or planned receipt throughout the study. Suppose systemic corticosteroids have been administered short-term (<14 days) to treat an acute illness. In that case, participants should not be enrolled in the study until corticosteroid therapy has been discontinued for at least 28 days before study intervention administration. Inhaled/nebulized, intra-articular, intra-bursal, or topical (skin or eyes) corticosteroids are permitted.

13. Receipt of blood/plasma products or immunoglobulin from 60 days before study intervention administration or planned receipt throughout the study.

Prior/Concurrent Clinical Study Experience:

14. Participation in other studies involving study intervention within 28 days before study entry and/or during study participation.

15. Previous participation in other studies involving study intervention containing lipid nanoparticles.

Diagnostic Assessments:

16. Positive serological test for SARS-CoV-2 IgM and/or IgG antibodies at the screening visit.

17. Any screening hematology and/or blood chemistry laboratory value that meets the definition of a > Grade 1 abnormality.

18. Positive test for HIV, hepatitis B surface antigen (HBsAg), hepatitis B core antibodies (HBc Abs), or hepatitis C virus antibodies (HCV Abs) at the screening visit.

19. SARS-CoV-2 PCR-positive nasal swab within 24 hours before receipt of the study intervention.

## 5.5. Screen Failures

Screen failures are defined as participants who consent to participate in the clinical study but are not subsequently randomly assigned to study intervention. A minimal set of screen failure information is required to ensure transparent reporting of screen failure participants to meet the CONSORT publishing requirements and to respond to queries from regulatory authorities. Minimal information includes demography, screen failure details, eligibility criteria, and any SAE.

Individuals not meeting the criteria for participation in this study (screen failure) may be rescreened under a different participant number.

## 5.6. Criteria for Temporarily Delaying Enrolment/Randomization/Study Intervention Administration

The following conditions are temporary or self-limiting, and a participant may be vaccinated once the condition(s) has/have resolved and no other exclusion criteria are met.

1. Current febrile illness (body temperature >38°C) or other acute illness within 48 hours before study intervention administration. This includes current symptoms that could represent a potential COVID-19 illness:

• New or increased cough;

• New or increased shortness of breath;

• New or increased sore throat;

• New or increased wheezing;

• New or increased sputum production;

• New or increased nasal congestion;

• New or increased nasal discharge;

• Loss of taste/smell.

2. Receipt of any seasonal or pandemic influenza vaccine within 14 days or any other nonstudy vaccine within 28 days before study intervention administration.

3. Anticipated receipt of any seasonal or pandemic influenza vaccine within 14 days or any other nonstudy vaccine within 28 days after study intervention administration.

4. Receipt of short-term (<14 days) systemic corticosteroids. Study intervention administration should be delayed until systemic corticosteroid use has been discontinued for at least 28 days. Inhaled/nebulized, intra-articular, intra-bursal, or topical (skin or eyes) corticosteroids are permitted.

# 6. STUDY INTERVENTION

Study intervention is defined as any investigational intervention(s), marketed product(s), placebo, medical device(s), or study procedure(s) intended to be administered to a study participant according to the study protocol.

The study may evaluate single-dose and 2-dose schedules of edible vaccine candidates for active immunization against COVID-19. Animals are expected to participate for up to a maximum of approximately two months. Participants are expected to participate for up to a maximum of approximately 1,5 months.

## 6.1. Study Intervention(s) Administered

| **Intervention Name** | **Animals (Mice)** | **Humans** |
| --- | --- | --- |
| **Type** | Vaccine | Vaccine |
| **Dose Formulation** | Edible | Edible |
| **Unit Dose Strength(s)** | ≈0.77 µg S1 protein daily/two times spaced seven days course (for TOMAVAC)  ≈16×10^6^ infectious units/two times spaced seven days course (for AstraZeneca) | ≈38.5 µg S1 protein daily/one time three days unspaced course |
| **Route of Administration** | Oral (for TOMAVAC)  Parenteral (for AstraZeneca) | Oral |
| **Use** | Experimental | Experimental |

### 6.1.1. Administration

A two-dose (days 1-7 and days 14-21), seven days-spaced force-feeding immunizations were carried out in mice using a syringe (for TOMAVAC).

A two-dose (day 0 and day 14), seven days-spaced parenteral immunizations were carried out in mice (for AstraZeneca). Each dose (75 µl of working solution of AstraZeneca vaccine) was planned to be administered by intramuscular injection in compliance with asepsis rules.

The experimental group of volunteers was provided and asked to take TOMAVAC on an empty stomach once a day for three consecutive days, 20-30 minutes before dining.

## 6.2. Measures to Minimize Bias: Randomization and Blinding

### 6.2.1. Allocation to Study Intervention

Allocation (randomization) of participants to vaccine groups will proceed randomly.

### 6.2.2. Blinding of Site Personnel

Individuals evaluating animal and participant safety and receptor binding domain-specific neutralizing antibodies in blood serum will be blinded.

### 6.2.3. Blinding of the Center

All laboratory testing personnel performing serology assays will remain blinded to the study intervention assigned/received throughout the study.

Those study team members who are involved in ensuring that protocol requirements for study intervention preparation, handling, allocation, and administration are fulfilled at the site will be unblinded for the duration of the study (e.g., unblinded study manager, unblinded clinical research associate). Unblinded clinician(s) who are not direct members of the study team will review unblinded protocol deviations.

## 6.3. Study Intervention Compliance

When participants are dosed at the site, they will receive study intervention directly from the investigator or designee under medical supervision. The date and time of each dose administered in the clinic will be recorded in the source documents and recorded in the CRF. The dose of study intervention and study participant identification will be confirmed at the time of dosing by a member of the study site staff other than the person administering the study intervention.

## 6.4. Concomitant Therapy

The following concomitant medications and vaccinations will be recorded in the CRF:

6.4.1. All vaccinations received from 28 days before study enrolment until the follow-up visit.

6.4.2. Prohibited medications will be recorded, including start and stop dates, name of the medication, dose, unit, route, and frequency.

6.4.3. Additionally, for participants, all current medication at baseline will be recorded, including start date, name of the medication, dose, unit, route, and frequency.

### 6.4.1. Prohibited During the Study

Receipt of the following vaccines and medications during the periods listed below may exclude a participant from the per-protocol analysis and may require vaccinations to be discontinued in that participant. Medications should not be withheld if required for a participant’s medical care.

Unless considered medically necessary, no vaccines other than study intervention should be administered within 28 days before and 28 days after the study vaccination. One exception to this is that seasonal and pandemic influenza vaccines can be given at least 14 days after, or at least 14 days before, the administration of the study intervention.

Receipt of chronic systemic treatment with known immunosuppressant medications or radiotherapy within 60 days before enrolment through the conclusion of the study.

Receipt of systemic corticosteroids (>20 mg/day of prednisone or equivalent) for >14 days is prohibited.

Receipt of blood/plasma products or immunoglobulins within 6 months before enrolment through the conclusion of the study.

Receipt of any other (non-study) coronavirus vaccine at any time before or during study participation is prohibited.

Prophylactic antipyretics and other pain medication to prevent symptoms associated with study intervention administration are not permitted. However, if a participant is taking medication for another condition, even if it may have antipyretic or pain-relieving properties, it should not be withheld before study vaccination.

### 6.4.2. Permitted During the Study

The use of antipyretics and other pain medication to treat symptoms associated with study intervention administration or ongoing conditions is permitted.

Medication other than that described as prohibited required for treatment of pre-existing stable conditions is permitted.

Inhaled, topical, or localized injections of corticosteroids (e.g., intra-articular or intra-bursal administration) are permitted.

## 6.5. Dose Modification

Individual participant dose modifications will not be made in this study.

## 6.6. Intervention After the End of the Study

No intervention will be provided to study participants at the end of the study.

# 7. DISCONTINUATION OF STUDY INTERVENTION AND PARTICIPANT DISCONTINUATION/WITHDRAWAL

## 7.1. Discontinuation of Study Intervention

In rare instances, it may be necessary for a participant to permanently discontinue study intervention (definitive discontinuation). Reasons for definitive discontinuation of study intervention may include the following: AEs; participant request, investigator request; pregnancy; protocol deviation (including no longer meeting all the inclusion criteria or meeting one or more exclusion criteria).

Note that discontinuing study intervention does not represent a withdrawal from the study. Per the study estimands, if study intervention is definitively discontinued, the participant will remain in the study to be evaluated for safety, immunogenicity, and potential efficacy.

In the event of discontinuation of study intervention, it must be documented on the appropriate CRF/in the medical records whether the participant is discontinuing further receipt of study intervention or also from study procedures, posttreatment study follow-up, and/or future collection of additional information.

## 7.2. Participant Discontinuation/Withdrawal from the Study

A participant may withdraw from the study at any time at his/her request. Reasons for discontinuation from the study may include the following:

• Refused further follow-up;

• Lost to follow-up;

• Death;

• Study terminated by the Center;

• AEs;

• Participant request;

• Investigator request;

• Protocol deviation.

If a participant does not return for a scheduled visit, every effort should be made to contact the participant. All attempts to contact the participant and information received during contact attempts must be documented in the participant’s source document. In any circumstance, every effort should be made to document participant outcomes, if possible.

The investigator or his or her designee should capture the reason for withdrawal in the CRF for all participants.

If a participant withdraws from the study, he/she may request the destruction of any remaining samples taken and not tested, and the investigator must document any such requests in the site study records and notify the Center accordingly.

If the participant withdraws from the study and also withdraws consent for disclosure of future information, no further evaluations should be performed, and no additional data should be collected. The Center may retain and continue to use any data collected before such withdrawal of consent.

Lack of completion of all or any withdrawal/early termination procedures will not be viewed as protocol deviations as long as the participant’s safety is preserved.

### 7.2.1. Withdrawal of Consent

Participants who request to discontinue receipt of study intervention will remain in the study and must continue to be followed for protocol-specified follow-up procedures. The only exception is when a participant expressly withdraws consent for any subsequent communication with him or her or those previously authorized by the participant to provide this information. Participants should notify the investigator in writing of the decision to withdraw consent from future follow-up whenever possible. The withdrawal of consent should be explained in detail in the medical records by the investigator as to whether the withdrawal is only from further receipt of study intervention or also from study procedures and/or post-treatment study follow-up and entered on the appropriate CRF page. In the event that vital status (whether the participant is alive or dead) is being measured, publicly available information should be used to determine vital status only as appropriately directed by local law.

## 7.3. Lost to Follow-up

A participant will be considered lost to follow-up if he or she repeatedly fails to return for scheduled visits and is unable to be contacted by the study site.

The following actions must be taken if a participant fails to attend a required study visit:

• The site must attempt to contact the participant and reschedule the missed visit as soon as possible and counsel the participant on the importance of maintaining the assigned visit schedule and ascertain whether or not the participant wishes to and/or should continue in the study;

• Before a participant is deemed lost to follow-up, the investigator or designee must make every effort to regain contact with the participant (where possible, three telephone calls and, if necessary, a certified letter to the participant’s last known mailing address or local equivalent methods). These contact attempts should be documented in the participant’s medical record;

• Should the participant continue to be unreachable, he/she will be considered to have withdrawn from the study.

# 8. STUDY ASSESSMENTS AND PROCEDURES

The investigator (or an appropriate delegate at the investigator site) must obtain a signed and dated ICD before performing any study-specific procedures.

The full date of birth will be collected to critically evaluate the immune response and safety profile by age.

Protocol waivers or exemptions are not allowed.

Safety issues should be discussed with the participant immediately upon occurrence or awareness to determine whether the participant should continue or discontinue the study intervention.

Adherence to the study design requirements, including those specified in the SoA, is essential and required for study conduct.

All screening evaluations must be completed and reviewed to confirm that potential participants meet all eligibility criteria. The investigator will maintain a screening log to record details of all participants screened and to confirm eligibility or record reasons for screening failure, as applicable.

Every effort should be made to ensure that protocol-required tests and procedures are completed as described. However, it is anticipated that from time to time, there may be circumstances outside the investigator’s control that may make it unfeasible to perform the test. In these cases, the investigator must take all steps necessary to ensure the safety and well-being of the participant. When a protocol-required test cannot be performed, the investigator will document the reason for the missed examination and any corrective and preventive actions that he or she has taken to ensure that required processes are adhered to as soon as possible. The study team must be informed of these incidents promptly.

For samples being collected and shipped, detailed collection, processing, storage, and shipment instructions and contact information will be provided to the investigator site before the initiation of the study.

The total blood sampling volume for individual participants in this study is approximately 1 ml for each animal and 5 mL for each participant.

The total intestinal lavage fluid sampling volume for individual animals in this study is approximately 5 ml for each animal.

Blood sampling and intestinal lavage fluid from animals will be carried out during decapitation.

Additionally, 15 mL of blood may be taken in humans at an unplanned convalescent visit at any time a participant develops respiratory symptoms indicating a potential COVID-19 infection. Additional blood samples may be taken for safety assessments, provided the total volume taken during the study does not exceed 50 mL for 30 consecutive days.

## 8.1. Efficacy and/or Immunogenicity Assessments in Animals

Efficacy will be assessed throughout an animal’s involvement in the study through surveillance for potential cases of COVID-19.

Efficacy and/or immunogenicity assessments in animals will be performed on days 14, 28, 42, and 56 after the first dose of the vaccine.

Serum samples will be obtained for immunogenicity testing at the visits specified in the SoA. The following assays will be performed:

• SARS-CoV-2 Spike RBD Antibody Titer Assay Kit (Mouse)

• SARS-CoV-2-specific surrogate virus neutralization test (sVNT)

• Mouse Anti-2019 nCoV(S)IgA ELISA Kit

## 8.2. Efficacy and/or Immunogenicity Assessments in Humans

Efficacy will be assessed throughout a participant’s involvement in the study through surveillance for potential cases of COVID-19. If, at any time, a participant develops any acute respiratory illness, he or she will be considered to have COVID-19 disease potentially. In this circumstance, the participant should contact the Center of Genomics and Bioinformatics of the Academy of Sciences of Uzbekistan, and assessments should be conducted as specified in the SoA. The assessments will include a nasal (mid-turbinate) swab, which will be tested using a reverse transcription-polymerase chain reaction (RT- PCR) test, or other equivalent nucleic acids amplification-based test to detect SARS-CoV-2. In addition, clinical information and results from local standard-of-care tests will be assessed. Four definitions of potential SARS-CoV-2-related cases will be considered:

• Centrally confirmed COVID-19: presence of at least 1 symptom and SARS-CoV-2 RT- PCR positive test

• Locally confirmed COVID-19: presence of at least 1 symptom and investigator-confirmed SARS-CoV-2 RT- PCR positive test

• Centrally confirmed symptomatic seroconversion to SARS-CoV-2 (exploratory): the presence of at least one symptom and a positive nonvaccine antigen SARS-CoV-2 antibody result in a participant whose most recent prior nonvaccine antigen SARS-CoV-2 antibody result was negative

• Centrally confirmed asymptomatic seroconversion to SARS-CoV-2 (exploratory): positive nonvaccine antigen SARS-CoV-2 antibody results in a participant with a prior nonvaccine antigen SARS-CoV-2 antibody result was negative.

Serum and intestinal lavage fluid samples will be obtained for immunogenicity testing at the visits specified in the SoA. The following assays will be performed:

• SARS-CoV-2-specific surrogate virus neutralization test (sVNT)

• SARS-CoV-2 RBD-specific IgG chemiluminescence immunoassay

• Mouse Anti-2019 nCoV(S)IgA ELISA Kit

Human efficacy and/or immunogenicity will be assessed at 7, 14, and 21 days post-vaccination.

### 8.2.1. Biological Samples

Blood, intestinal lavage fluid and nasal swab samples will be used only for scientific research. Each sample will be labeled with a code so that the laboratory personnel testing the samples will not know the participant’s identity. Samples that remain after performing assays outlined in the protocol may be stored. If allowed by the ICD, stored samples may be used for additional testing to understand better the immune responses to the vaccine(s) under study in this protocol, to inform the development of other products, and/or for vaccine-related assay work supporting vaccine programs. No testing of the participant’s DNA will be performed.

The participant may request that his or her samples, if still identifiable, be destroyed at any time; however, any data already collected from those samples will still be used for this research. The biological samples may be shared with other researchers as long as confidentiality is maintained and no testing of the participant’s DNA is performed.

## 8.3. Safety Assessments

Planned time points for all safety assessments are provided in the SoA. Unscheduled clinical laboratory measurements may be obtained at any time during the study to assess any perceived safety issues.

A clinical assessment, including medical history, will be performed on all participants on his/her first visit to establish a baseline. Significant medical history and observations from any physical examination, if completed, will be documented in the CRF.

AEs and SAEs are collected, recorded, and reported as defined in Section 8.4.

Acute reactions within the first 4 hours after administration of the study intervention will be assessed and documented in the AE CRF.

The safety parameters also include e-diary reports of local reactions and systemic events (including fever) and the use of antipyretic medication that occur seven days after the study intervention's administration. These prospectively self-collected occurrences of local reactions and systemic events are graded as described in Section 8.3.1.

### 8.3.1. Diary

Participants will be required to complete a diary for registration of any adverse effects. The participant will be asked to monitor and record local reactions, systemic events, and antipyretic medication usage for seven days following the administration of the study intervention. Data on local reactions and systemic events must be transferred to the Center of Genomics and Bioinformatics of the Academy of Sciences of Uzbekistan.

Investigators (or designees) will be required to review the diary data frequently as part of the ongoing safety review.

The investigator or designee must obtain stop dates from the participant for any ongoing local reactions, systemic events, or use of antipyretic medication on the last day that the e-diary was completed. The stop dates should be documented in the source documents and the information entered in the CRF.

#### 8.3.1.1. Grading Scales

The grading scales used in this study to assess local reactions and systemic events, as described below, are derived from the FDA Center for Biologics Evaluation and Research (CBER) guidelines on toxicity grading scales for healthy adult volunteers enrolled in preventive vaccine clinical trials.

#### 8.3.1.2. Local Reactions

During the diary reporting period, participants will be asked to assess erythema and allergic reactions and record the symptoms. If local reactions persist beyond the end of the diary period following vaccination, the participant will be requested to report that information. The investigator will enter this additional information in the CRF.

Erythema will be categorized during analysis as absent, mild, moderate, or severe based on the grading scale in Table 1. The participant will assess allergic reactions as absent, mild, moderate, or severe.

If a Grade 3 local reaction is reported in the diary, a telephone contact should occur to ascertain further details and determine whether a site visit is clinically indicated. Only an investigator or medically qualified person can classify a participant’s local reaction as Grade 4. Suppose a participant experiences a confirmed Grade 4 local reaction. In that case, the investigator must immediately notify the Center of Genomics and Bioinformatics. If it is determined to be related to the administration of the study intervention, further vaccinations will be discontinued in that participant.

#### 8.3.1.3. Systemic Events

During the diary reporting period, participants will be asked to assess vomiting, diarrhea, headache, fatigue, chills, new or worsened muscle pain, and new or worsened joint pain and to record the symptoms in the diary. The symptoms will be assessed by the participant as absent, mild, moderate, or severe according to the grading scale in Table 1.

If a Grade 3 systemic event is reported in the diary, a telephone contact should occur to ascertain further details and determine whether a site visit is clinically indicated. Only an investigator or medically qualified person can classify a participant’s systemic event as Grade 4. Suppose a participant experiences a confirmed Grade 4 systemic event. In that case, the investigator must immediately notify the Center. If it is determined to be related to the administration of the study intervention, further vaccinations will be discontinued for that participant.

**Table 1.** Systemic Event Grading Scale

|  | **Mild (Grade 1)** | **Moderate (Grade 2)** | **Severe (Grade 3)** | **Potentially Life-Threatening (Grade 4)** |
| --- | --- | --- | --- | --- |
| **Vomiting** | 1-2 times in  24 hours | >2 times in 24 hours | Requires IV hydration | Emergency room visit or hospitalization for hypotensive shock |
| **Diarrhoea** | 2 to 3 loose stools in 24 hours | 4 to 5 loose stools in 24 hours | Six or more loose stools in 24 hours | Emergency room visit or hospitalization for severe diarrhea |
| **Headache** | Does not interfere with the activity | Some interference with activity | Prevents daily routine activity | Emergency room visit or hospitalization for severe headache |
| **Fatigue/ tiredness** | Does not interfere with the activity | Some interference with activity | Prevents daily routine activity | Emergency room visit or hospitalization for severe fatigue |
| **Chills** | Does not interfere with the activity | Some interference with activity | Prevents daily routine activity | Emergency room visit or hospitalization for severe chills |
| **New or worsened muscle pain** | Does not interfere with the activity | Some interference with activity | Prevents daily routine activity | Emergency room visit or hospitalization for severe new or worsened muscle pain |
| **New or worsened joint pain** | Does not interfere with the activity | Some interference with activity | Prevents daily routine activity | Emergency room visit or hospitalization for severe new or worsened joint pain. |

#### 8.3.1.4. Fever

To record information on fever, a thermometer will be given instructions on how to measure temperature at home. The temperature will be recorded in the diary in the evening daily during the diary reporting period. It will also be collected at any time during the diary data collection periods when fever is suspected. Fever is defined as an oral temperature of >38.0°C. The highest temperature for each day will be recorded in the diary. The temperature will be measured and recorded to 1 decimal place and then categorized during analysis according to the scale shown in Table 1.

If a fever of >39.0°C is reported in the diary, telephone contact should occur to ascertain further details and determine whether a site visit is clinically indicated. Only an investigator or medically qualified person can confirm a participant’s fever as >40.0°C. Suppose a participant experiences a confirmed fever >40.0°C. In that case, the investigator must immediately notify the Center of Genomics and Bioinformatics. If it is determined to be related to the administration of the study intervention, further vaccinations will be discontinued in that participant.

**Table 4.** Scale for Fever

| 38.0-38.4°C |
| --- |
| 38.5-38.9°C |
| 39.0-40.0°C |
| >40.0°C |

#### 8.3.1.5. Antipyretic Medication

The use of antipyretic medication to treat symptoms associated with study intervention administration has to be recorded in the diary daily during the reporting period (Day 1 to Day 7).

### 8.3.2. Stopping Rules

Based on a review of AE data and diary reactogenicity data, the following stopping rules are in place for all sentinel-cohort participants. These data will be monitored on an ongoing basis by the investigator (or medically qualified designee) and the Center of Genomics and Bioinformatics to promptly identify and flag any event that potentially contributes to a stopping rule.

The Center study team will be unblinded, so they will be able to assess whether or not a stopping rule has been met based on a participant’s study intervention allocation.

If Center personnel confirm that a stopping rule is met, the following actions will commence:

• The IRC will review all appropriate data.

• The stopping rule will PAUSE randomization and study intervention administration for the impacted vaccine candidate at all dose levels and age groups.

• The DMC will review all appropriate data.

• For all participants vaccinated, all other routine study conduct activities, including ongoing data entry, reporting of AEs, participant diary completion, blood sample collection, and participant follow-up, will continue during the pause.

A stopping rule is met if any of the following rules occur after administering the investigational vaccine; data from placebo recipients will not contribute to the stopping rules. Diary data confirmed by the investigator as being entered by the participant in error will not contribute toward a stopping rule.

TOMAVAC vaccine candidates will be evaluated for contribution to stopping rules individually.

However, the recommendations may include halting or continuing randomization with the TOMAVAC vaccine candidate.

**Stopping Rule Criteria for TOMAVAC Vaccine Candidate:**

1. If any participant vaccinated with the vaccine candidate develops an SAE that the investigator assesses as possibly related or for which there is no alternative, plausible, attributable cause.

2. If any participant vaccinated with the vaccine candidate develops a Grade 4 local reaction or systemic event within seven days after vaccination that is assessed as possibly related by the investigator or for which there is no alternative, plausible, attributable cause.

3. If any participant vaccinated with the vaccine candidate develops a fever >40.0°C for at least one daily measurement within seven days after vaccination that is assessed as possibly related by the investigator or for which there is no alternative, plausible, attributable cause.

4. If any 2 participants vaccinated with the vaccine candidate report the same or similar severe (Grade 3) AE within 21 days after vaccination, assessed as possibly related by the investigator, or for which there is no alternative, plausible, attributable cause.

5. If any participant dies or requires ICU admission due to SARS-CoV-2 infection if this stopping rule is met, all available clinical and preclinical safety and immunogenicity data should be reviewed to evaluate for enhanced COVID-19 disease.

### 8.3.3. Surveillance of Events That Could Represent Enhanced COVID-19 Disease

As this is an open-label study, the Center of Genomics and Bioinformatics will conduct unblinded reviews of the data during the study, including for safety assessment.

Participants in all stages of the study will be surveyed for potential COVID-19 illness from Visit 1 onwards (see Section 8.14). All RT-PCR-confirmed cases will be reviewed contemporaneously by the IRC and the DMC. In addition, instances of symptomatic and asymptomatic seroconversion to SARS-CoV-2 (see Section 8.2) will be reviewed.

The purpose of these reviews will be to identify whether any features of each case appear unusual, in particular greater severity, compared to available information at the time of examination. Indicators of severity may include accelerated deterioration, need for hospitalization, need for ventilation, or death. Observed rates of these indicators will be compared with what could be expected in a similar population to the study participants based on available information at the time of review. Since the DMC can review unblinded information, it will also be able to compare cases in active vaccine and placebo recipients.

## 8.4. Adverse Events and Serious Adverse Events

The definitions of an AE and an SAE can be found in Appendix B.

The participant will report AEs.

The investigator and any qualified designees are responsible for detecting, documenting, and recording events that meet the definition of an AE or SAE and remain responsible for pursuing and obtaining adequate information both for determining the outcome and assessing whether the event meets the criteria for classification as an SAE or caused the participant to discontinue the study intervention (see Section 7.1).

Each participant will be questioned about the occurrence of AEs.

In addition, the investigator may be requested specific follow-up information in an expedited fashion.

### 8.4.1. Time Period and Frequency for Collecting AE and SAE Information

The time period for actively eliciting and collecting AEs and SAEs (“active collection period”) for each participant begins from the time the participant provides informed consent, which is obtained before the participant’s involvement in the study (i.e., before undergoing any study-related procedure and/or receiving study intervention). In addition, any AEs occurring up to 48 hours after each subsequent blood draw must be recorded on the CRF.

SAEs will be collected from the time the participant provides informed consent to approximately six months after the dose of the study intervention.

Follow-up by the investigator continues throughout and after the active collection period and until the AE or SAE or its sequelae resolve or stabilize at a level acceptable to the investigator.

For participants who are screen failures, the active collection period ends when the screen failure status is determined.

If the participant withdraws from the study and also withdraws consent for future information collection, the active collection period ends when consent is withdrawn.

Suppose a participant definitively discontinues or temporarily discontinues study intervention because of an AE or SAE. In that case, the AE or SAE must be recorded on the CRF, and the SAE must be reported using the Vaccines SAE Report Form.

Investigators are not obligated to actively seek AEs or SAEs after the participant has concluded study participation. However, suppose the investigator learns of any SAE, including death, at any time after a participant has completed the study, and he/she considers the event to be reasonably related to the study intervention. In that case, the investigator must promptly report the SAE.

#### 8.4.1.1. Reporting SAEs

All SAEs occurring in a participant during the active collection period, as described in Section 8.4.1, are reported on the Vaccines SAE Report Form immediately upon awareness. Under no circumstance should this exceed 24 hours, as indicated in Appendix B, the investigator will submit any updated SAE data within 24 hours of it being available.

#### 8.4.1.2. Recording Nonserious AEs and SAEs on the CRF

All nonserious AEs and SAEs occurring in a participant during the active collection period, as described in Section 8.4.1, are recorded on the CRF. AEs and SAEs that begin after obtaining informed consent but before the start of the study intervention will be recorded on the Medical History/Current Medical Conditions section of the CRF, not the AE section. AEs and SAEs that begin after the start of the study intervention are recorded on the AE section of the CRF.

The investigator is to record on the CRF all directly observed, and all spontaneously reported AEs and SAEs reported by the participant.

### 8.4.2. Method of Detecting AEs and SAEs

The method of recording, evaluating, and assessing the causality of AEs and SAEs and the procedures for completing and transmitting SAE reports are provided in Appendix B.

Care will be taken not to introduce bias when detecting AEs and/or SAEs. Open-ended and non-leading verbal questioning of the participant is the preferred method to inquire about AE occurrences.

### 8.4.3. Follow-up of AEs and SAEs

After the initial AE/SAE report, the investigator must proactively follow each participant at subsequent visits/contacts. For each event, the investigator must pursue and obtain adequate information until resolution, stabilization, the event is otherwise explained, or the participant is lost to follow-up (as defined in Section 7.3).

In general, follow-up information will include a description of the event in sufficient detail to allow for a complete medical assessment of the case and independent determination of possible causality. Any information relevant to the event, such as concomitant medications and illnesses, must be provided. In case of a participant’s death, a summary of available autopsy findings must be submitted to the Center of Genomics and Bioinformatics as soon as possible.

Further information on follow-up procedures is given in Appendix B.

### 8.4.4. Regulatory Reporting Requirements for SAEs

Prompt notification of an SAE is essential so that legal obligations and ethical responsibilities towards the safety of participants and the safety of a study intervention under clinical investigation are met.

### 8.4.5. Medication Errors

Medication errors may result from the administration or consumption of the study intervention by the wrong participant at the wrong time or dosage strength.

Exposures to the study intervention under study may occur in clinical trial settings, such as medication errors.

| **Safety Event** | **Recorded on the CRF** | **Reported Within 24 Hours of Awareness** |
| --- | --- | --- |
| Medication errors | All (regardless of whether associated with an AE) | Only if associated with an SAE |

Medication errors include:

• Medication errors involving participant exposure to the study intervention;

• Potential medication errors or uses outside of what is foreseen in the protocol that does or does not involve the study participant;

• The administration of expired study intervention;

• The administration of an incorrect study intervention;

• The administration of incorrect dosage.

Such medication errors occurring to a study participant are to be captured on the medication error page of the CRF, which is a specific version of the AE page.

If a medication dosing error occurs, the Center should be notified immediately.

Whether or not the medication error is accompanied by an AE, as determined by the investigator, the medication error is recorded on the medication error page of the CRF and, if applicable, any associated AE(s), severe and nonserious, are recorded on the AE page of the CRF.

Medication errors should be reported to the Center of Genomics and Bioinformatics within 24 hours.

### 8.5. Treatment of Overdose

For this study, any dose of study intervention greater than one dose of study intervention within 24 hours will be considered an overdose.

In the event of an overdose, the investigator should:

1. Contact the medical monitor immediately.

2. Closely monitor the participant for any AEs/SAEs.

3. Document the quantity of the excess dose as well as the duration of the overdose in the CRF.

4. Overdose is reportable to the Center of Genomics and Bioinformatics only when associated with an SAE.

Decisions regarding dose interruptions or modifications will be made by the investigator in consultation with the medical monitor based on the clinical evaluation of the participant.

### 8.6. Pharmacokinetics

Pharmacokinetic parameters are not evaluated in this study.

### 8.7. Pharmacodynamics

Pharmacodynamical parameters are not evaluated in this study.

### 8.8. Genetics

Genetics (specified analyses) are not evaluated in this study.

### 8.9. Biomarkers

Biomarkers are not evaluated in this study.

### 8.10. Immunogenicity Assessments

Immunogenicity assessments are described in Sections 8.1 and 8.2.

### 8.11. Health Economics

This study does not evaluate health economics/medical resource utilization and health economics parameters.

### 8.12. Study Procedures

### 8.12.1. Sentinel Cohorts for Humans

#### 8.12.1.1. Screening: (0 to 14 Days Before Visit 1)

Before enrolment and any study-related procedures are performed, voluntary, written study-specific informed consent will be obtained from the participant. Each signature on the ICD must be personally dated by the signatory. The investigator will also sign the ICD. A copy of the signed and dated ICD must be given to the participant. The source data must reflect that informed consent was obtained before participation in the study.

It is anticipated that the procedures below will be conducted stepwise; however, the visit can occur over more than one day.

• Assign a single participant number using the IRT system.

• Obtain the participant’s demography (including date of birth, sex, race, and ethnicity). The full date of birth will be collected to critically evaluate the immune response and safety profile by age.

• Obtain any medical history of clinical significance.

• Obtain details of any medications currently taken.

• Perform physical examination including vital signs (weight, height, body temperature, pulse rate, and seated blood pressure), evaluating any clinically significant abnormalities within the following body systems: general appearance; skin; head, eyes, ears, nose, and throat; heart; lungs; abdomen; musculoskeletal; extremities; neurological; and lymph nodes.

• Collect a blood sample (approximately 5 mL) for serological assessment of prior COVID-19 infection.

• Discuss contraceptive use.

• Record nonstudy vaccinations.

• Ensure and document that all of the inclusion criteria and none of the exclusion criteria are met.

• Record AEs. AEs that occur prior to dosing should be noted on the Medical History CRF.

• Ask the participant to contact the site staff or investigator immediately if any significant illness or hospitalization occurs.

• Ask the participant to contact the site staff or investigator immediately if he or she experiences any respiratory symptoms.

• Schedule an appointment for the participant to return for the next study visit.

• Complete the source documents.

• Complete the CRF.

#### 8.12.1.2. Visit 1 - Vaccination: (Day 1)

It is anticipated that the procedures below will be conducted stepwise; ensure that procedures listed prior to administering the vaccine are conducted prior to vaccination.

• Record AEs as described in Section 8.4.

• Perform physical examination including vital signs (body temperature, pulse rate, and seated blood pressure), evaluating any clinically significant abnormalities within the following body systems: general appearance; skin; head, eyes, ears, nose, and throat; heart; lungs; abdomen; musculoskeletal; extremities; neurological; and lymph nodes.

• Discuss contraceptive use.

• Record nonstudy vaccinations.

• Obtain nasal (midturbinate) swabs. One will be tested within 24 hours, and vaccination will proceed only if it is PCR-negative for SARS-CoV-2 genomes.

• Ensure and document that all of the inclusion criteria and none of the exclusion criteria are met.

• Ensure that the participant meets none of the temporary delay criteria.

• Obtain the participant’s randomization number and study intervention allocation using the IRT system. Either blinded site staff or unblinded site staff members may obtain this information.

• Collect a blood sample (approximately 5 mL) for immunogenicity testing.

• Unblinded site staff member(s) will dispense one dose of the study vaccine for oral administration.

• Provide instructions on them for recording daily temperatures and measuring local reactions.

• Provide instructions on diary completion and ask the participant to complete the diary from Day 1 to Day 7, with Day 1 being the day of vaccination.

• Ask the participant to contact the site staff or investigator immediately if he or she experiences any of the following from Day 1 to Day 7 after vaccination (where Day 1 is the day of vaccination) to determine if an unscheduled reactogenicity visit is required:

• Fever >39.0°C.

• Any severe systemic event.

• Ask the participant to contact the site staff or investigator if a medically attended event (e.g., doctor’s visit, emergency room visit) or hospitalization occurs.

• Ask the participant to contact the site staff or investigator immediately if he or she experiences any respiratory symptoms.

• Schedule an appointment for the participant to return for the next study visit.

• Remind the participant to bring the diary to the next visit.

• Complete the source documents.

• The investigator or an authorized designee completes the CRFs, and an unblinded dispenser/administrator updates the study intervention accountability records.

• The investigator or appropriately qualified designee reviews the diary data online following vaccination to evaluate participant compliance and as part of the ongoing safety review. Daily review is optimal during the active diary period.

#### 8.12.1.3. Visit 2 - Next-Day Follow-up Visit (Vaccination): (1 to 3 Days After Visit 1)

• Record AEs.

• Perform physical examination, including vital signs (body temperature, pulse rate, and

seated blood pressure), evaluating any clinically significant abnormalities within the following body systems: general appearance; skin; head, eyes, ears, nose, and throat; heart; lungs; abdomen; musculoskeletal; extremities; neurological; and lymph nodes.

• Record non-study vaccinations.

• Record details of any prohibited medications received by the participant if required for his or her clinical care.

• Discuss contraceptive use.

• Ask the participant to contact the site staff or investigator immediately if he or she experiences any of the following from Day 1 to Day 7 after vaccination (where Day 1 is the day of vaccination) to determine if an unscheduled reactogenicity visit is required:

• Fever >39.0°C.

• Any severe systemic event.

• Ask the participant to contact the site staff or investigator if a medically attended event (e.g., doctor’s visit, emergency room visit) or hospitalization occurs.

• Ask the participant to contact the site staff or investigator immediately if he or she experiences any respiratory symptoms.

• Schedule an appointment for the participant to return for the next study visit.

• Remind the participant to bring the diary to the next visit.

• Complete the source documents.

• The investigator or an authorized designee completes the CRFs.

• The investigator or appropriately qualified designee reviews the e-diary data online

following vaccination to evaluate participant compliance and as part of the ongoing safety review. Daily review is optimal during the active diary period.

#### 8.12.1.4. Visit 3 - 1-Week Follow-up Visit (Vaccination 1): (6 to 8 Days After Visit 1)

• Record AEs.

• Perform physical examination including vital signs (body temperature, pulse rate, and seated blood pressure), evaluating any clinically significant abnormalities within the following body systems: general appearance; skin; head, eyes, ears, nose, and throat; heart; lungs; abdomen; musculoskeletal; extremities; neurological; and lymph nodes.

• Record non-study vaccinations.

• Record details of any prohibited medications received by the participant if required for his or her clinical care.

• Discuss contraceptive use.

• Collect a blood sample (approximately 5 mL) for immunogenicity testing.

• Ask the participant to contact the site staff or investigator immediately if he or she experiences any of the following from Day 1 to Day 7 after vaccination (where Day 1 is the day of vaccination) to determine if an unscheduled reactogenicity visit is required:

• Fever >39.0°C.

• Any allergic reactions.

• Any severe systemic event.

• Ask the participant to contact the site staff or investigator if a medically attended event (e.g., doctor’s visit, emergency room visit) or hospitalization occurs.

• Ask the participant to contact the site staff or investigator immediately if he or she experiences any respiratory symptoms.

• Schedule an appointment for the participant to return for the next study visit.

• Remind the participant to bring the diary to the next visit.

• Complete the source documents.

• The investigator or an authorized designee completes the CRFs.

#### 8.12.1.5. Visit 4 - 2-Week Follow-up Visit (Vaccination 1): (13 to 15 Days After Visit 1)

• Record AEs.

• Perform physical examination including vital signs (body temperature, pulse rate, and seated blood pressure), evaluating any clinically significant abnormalities within the following body systems: general appearance; skin; head, eyes, ears, nose, and throat; heart; lungs; abdomen; musculoskeletal; extremities; neurological; and lymph nodes.

• Record nonstudy vaccinations.

• Record details of any prohibited medications received by the participant if required for his or her clinical care.

• Discuss contraceptive use.

• Collect a blood sample (approximately 5 mL) for immunogenicity testing.

• Ask the participant to contact the site staff or investigator immediately if he or she experiences any of the following from Day 1 to Day 7 after vaccination (where Day 1 is the day of vaccination) to determine if an unscheduled reactogenicity visit is required:

• Fever >39.0°C.

• Any allergic reactions.

• Any severe systemic event.

• Ask the participant to contact the site staff or investigator if a medically attended event (e.g., doctor’s visit, emergency room visit) or hospitalization occurs.

• Ask the participant to contact the site staff or investigator immediately if he or she experiences any respiratory symptoms.

• Schedule an appointment for the participant to return for the next study visit.

• Remind the participant to bring the diary to the next visit.

• Complete the source documents.

• The investigator or an authorized designee completes the CRFs.

#### 8.12.1.6. Visit 5 - 3-Week Follow-up Visit (Vaccination 1): (20 to 22 Days After Visit 1)

• Record AEs.

• Perform physical examination including vital signs (body temperature, pulse rate, and seated blood pressure), evaluating any clinically significant abnormalities within the following body systems: general appearance; skin; head, eyes, ears, nose, and throat; heart; lungs; abdomen; musculoskeletal; extremities; neurological; and lymph nodes.

• Record nonstudy vaccinations.

• Record details of any prohibited medications received by the participant if required for his or her clinical care.

• Discuss contraceptive use.

• Collect a blood sample (approximately 5 mL) for immunogenicity testing.

• Ask the participant to contact the site staff or investigator immediately if he or she experiences any of the following from Day 1 to Day 7 after vaccination (where Day 1 is the day of vaccination) to determine if an unscheduled reactogenicity visit is required:

• Fever >39.0°C.

• Any allergic reactions.

• Any severe systemic event.

• Ask the participant to contact the site staff or investigator if a medically attended event (e.g., doctor’s visit, emergency room visit) or hospitalization occurs.

• Ask the participant to contact the site staff or investigator immediately if he or she experiences any respiratory symptoms.

• Schedule an appointment for the participant to return for the next study visit.

• Remind the participant to bring the diary to the next visit.

• Complete the source documents.

• The investigator or an authorized designee completes the CRFs.

#### 8.11.1.7. Visit 6 - 1-Month Follow-up Visit: (28 to 35 Days After Visit 4)

• Record AEs.

• Record nonstudy vaccinations.

• Record details of any prohibited medications received by the participant if required for his or her clinical care.

• Discuss contraceptive use.

• Ask the participant to contact the site staff or investigator if a medically attended event (e.g., doctor’s visit, emergency room visit) or hospitalization occurs.

• Ask the participant to contact the site staff or investigator immediately if he or she experiences any respiratory symptoms.

• Complete the source documents.

• The investigator or an authorized designee completes the CRFs.

### 8.12.2. Nonsentinel Cohorts for Humans

#### 8.12.2.1. Screening: (0 to 14 Days Before Visit 1)

Before enrolment and any study-related procedures are performed, voluntary, written study-specific informed consent will be obtained from the participant. Each signature on the ICD must be personally dated by the signatory. The investigator will also sign the ICD. A copy of the signed and dated ICD must be given to the participant. The source data must reflect that informed consent was obtained before participation in the study.

It is anticipated that the procedures below will be conducted stepwise; however, the visit can occur over more than one day.

• Assign a single participant number using the IRT system.

• Obtain the participant’s demography (including date of birth, sex, race, and ethnicity). The full date of birth will be collected to critically evaluate the immune response and safety profile by age.

• Obtain any medical history of clinical significance.

• Obtain details of any medications currently taken.

• Perform physical examination, including vital signs (weight, height, body temperature,

pulse rate and seated blood pressure), evaluating any clinically significant abnormalities within the following body systems: general appearance; skin; head, eyes, ears, nose, and throat; heart; lungs; abdomen; musculoskeletal; extremities; neurological; and lymph nodes.

• Collect a blood sample (approximately 5 mL) for serological assessment of prior COVID-19 infection.

• Discuss contraceptive use.

• Record nonstudy vaccinations as described in Section 6.4.

• Ensure and document that all of the inclusion criteria and none of the exclusion criteria are met.

• Record AEs as described in Section 8.4. AEs that occur prior to dosing should be noted on the Medical History CRF.

• Ask the participant to contact the site staff or investigator immediately if any significant illness or hospitalization occurs.

• Ask the participant to immediately contact the site staff or investigator if he or she experiences any respiratory symptoms, as detailed in Section 8.14.

• Schedule an appointment for the participant to return for the next study visit.

• Complete the source documents.

• Complete the CRF.

#### 8.12.2.2. Visit 1 - Vaccination: (Day 1)

It is anticipated that the procedures below will be conducted stepwise; ensure that procedures listed prior to administering the vaccine are conducted prior to vaccination.

• Record AEs as described in Section 8.4.

• Perform physical examination including vital signs (body temperature, pulse rate, and seated blood pressure), evaluating any clinically significant abnormalities within the following body systems: general appearance; skin; head, eyes, ears, nose, and throat; heart; lungs; abdomen; musculoskeletal; extremities; neurological; and lymph nodes.

• Discuss contraceptive use.

• Record non-study vaccinations.

• Obtain nasal (mid-turbinate) swabs. One will be tested within 24 hours, and vaccination will proceed only if it is PCR-negative for SARS-CoV-2 genomes.

• Ensure and document that all of the inclusion criteria and none of the exclusion criteria are met.

• Ensure that the participant meets none of the temporary delay criteria.

• Obtain the participant’s randomization number and study intervention allocation using the IRT system. Either blinded site staff or unblinded site staff members may obtain this information.

• Collect a blood sample (approximately 5 mL) for immunogenicity testing.

• Unblinded site staff member(s) will dispense one dose of the study vaccine for oral administration.

• Provide instructions on them for recording daily temperatures and measuring local reactions.

• Provide instructions on diary completion and ask the participant to complete the diary from Day 1 to Day 7, with Day 1 being the day of vaccination.

• Ask the participant to contact the site staff or investigator immediately if he or she experiences any of the following from Day 1 to Day 7 after vaccination (where Day 1 is the day of vaccination) to determine if an unscheduled reactogenicity visit is required:

• Fever >39.0°C.

• Any severe systemic event.

• Ask the participant to contact the site staff or investigator if a medically attended event (e.g., doctor’s visit, emergency room visit) or hospitalization occurs.

• Ask the participant to contact the site staff or investigator immediately if he or she experiences any respiratory symptoms.

• Schedule an appointment for the participant to return for the next study visit.

• Remind the participant to bring the diary to the next visit.

• Complete the source documents.

• The investigator or an authorized designee completes the CRFs, and an unblinded dispenser/administrator updates the study intervention accountability records.

• The investigator or appropriately qualified designee reviews the diary data online following vaccination to evaluate participant compliance and as part of the ongoing safety review. Daily review is optimal during the active diary period.

#### 8.12.2.3. Visit 2 - Next-Day Follow-up Visit (Vaccination): (1 to 3 Days After Visit 1)

• Record AEs.

• Perform physical examination, including vital signs (body temperature, pulse rate, and

seated blood pressure), evaluating any clinically significant abnormalities within the following body systems: general appearance; skin; head, eyes, ears, nose, and throat; heart; lungs; abdomen; musculoskeletal; extremities; neurological; and lymph nodes.

• Record non-study vaccinations.

• Record details of any prohibited medications received by the participant if required for his or her clinical care.

• Discuss contraceptive use.

• Ask the participant to contact the site staff or investigator immediately if he or she experiences any of the following from Day 1 to Day 7 after vaccination (where Day 1 is the day of vaccination) to determine if an unscheduled reactogenicity visit is required:

• Fever >39.0°C.

• Any severe systemic event.

• Ask the participant to contact the site staff or investigator if a medically attended event (e.g., doctor’s visit, emergency room visit) or hospitalization occurs.

• Ask the participant to contact the site staff or investigator immediately if he or she experiences any respiratory symptoms.

• Schedule an appointment for the participant to return for the next study visit.

• Remind the participant to bring the diary to the next visit.

• Complete the source documents.

• The investigator or an authorized designee completes the CRFs.

• The investigator or appropriately qualified designee reviews the e-diary data online

following vaccination to evaluate participant compliance and as part of the ongoing safety review. Daily review is optimal during the active diary period.

#### 8.12.2.4. Visit 3 - 1-Week Follow-up Visit 1: (6 to 8 Days After Visit 1)

• Record AEs.

• Perform physical examination including vital signs (body temperature, pulse rate, and seated blood pressure), evaluating any clinically significant abnormalities within the following body systems: general appearance; skin; head, eyes, ears, nose, and throat; heart; lungs; abdomen; musculoskeletal; extremities; neurological; and lymph nodes.

• Record non-study vaccinations.

• Record details of any prohibited medications received by the participant if required for his or her clinical care.

• Discuss contraceptive use.

• Collect a blood sample (approximately 5 mL) for immunogenicity testing.

• Ask the participant to contact the site staff or investigator immediately if he or she experiences any of the following from Day 1 to Day 7 after vaccination (where Day 1 is the day of vaccination) to determine if an unscheduled reactogenicity visit is required:

• Fever >39.0°C.

• Any allergic reactions.

• Any severe systemic event.

• Ask the participant to contact the site staff or investigator if a medically attended event (e.g., doctor’s visit, emergency room visit) or hospitalization occurs.

• Ask the participant to contact the site staff or investigator immediately if he or she experiences any respiratory symptoms.

• Schedule an appointment for the participant to return for the next study visit.

• Remind the participant to bring the diary to the next visit.

• Complete the source documents.

• The investigator or an authorized designee completes the CRFs.

#### 8.12.2.5. Visit 4 - 2-Week Follow-up Visit 1: (13 to 15 Days After Visit 1)

• Record AEs.

• Perform physical examination including vital signs (body temperature, pulse rate, and seated blood pressure), evaluating any clinically significant abnormalities within the following body systems: general appearance; skin; head, eyes, ears, nose, and throat; heart; lungs; abdomen; musculoskeletal; extremities; neurological; and lymph nodes.

• Record non-study vaccinations.

• Record details of any prohibited medications received by the participant if required for his or her clinical care.

• Discuss contraceptive use.

• Collect a blood sample (approximately 5 mL) for immunogenicity testing.

• Ask the participant to contact the site staff or investigator immediately if he or she experiences any of the following from Day 1 to Day 7 after vaccination (where Day 1 is the day of vaccination) to determine if an unscheduled reactogenicity visit is required:

• Fever >39.0°C.

• Any allergic reactions.

• Any severe systemic event.

• Ask the participant to contact the site staff or investigator if a medically attended event (e.g., doctor’s visit, emergency room visit) or hospitalization occurs.

• Ask the participant to contact the site staff or investigator immediately if he or she experiences any respiratory symptoms.

• Schedule an appointment for the participant to return for the next study visit.

• Remind the participant to bring the diary to the next visit.

• Complete the source documents.

• The investigator or an authorized designee completes the CRFs.

#### 8.12.2.6. Visit 5 - 3-Week Follow-up Visit 1: (20 to 22 Days After Visit 1)

• Record AEs.

• Perform physical examination including vital signs (body temperature, pulse rate, and seated blood pressure), evaluating any clinically significant abnormalities within the following body systems: general appearance; skin; head, eyes, ears, nose, and throat; heart; lungs; abdomen; musculoskeletal; extremities; neurological; and lymph nodes.

• Record nonstudy vaccinations.

• Record details of any prohibited medications received by the participant if required for his or her clinical care.

• Discuss contraceptive use.

• Collect a blood sample (approximately 5 mL) for immunogenicity testing.

• Ask the participant to contact the site staff or investigator immediately if he or she experiences any of the following from Day 1 to Day 7 after vaccination (where Day 1 is the day of vaccination) to determine if an unscheduled reactogenicity visit is required:

• Fever >39.0°C.

• Any allergic reactions.

• Any severe systemic event.

• Ask the participant to contact the site staff or investigator if a medically attended event (e.g., doctor’s visit, emergency room visit) or hospitalization occurs.

• Ask the participant to contact the site staff or investigator immediately if he or she experiences any respiratory symptoms.

• Schedule an appointment for the participant to return for the next study visit.

• Remind the participant to bring the diary to the next visit.

• Complete the source documents.

• The investigator or an authorized designee completes the CRFs.

#### 8.11.2.7. Visit 6 - 1-Month Follow-up Visit: (28 to 35 Days After Visit 4)

• Record AEs.

• Record non-study vaccinations.

• Record details of any prohibited medications received by the participant if required for his or her clinical care.

• Discuss contraceptive use.

• Ask the participant to contact the site staff or investigator if a medically attended event (e.g., doctor’s visit, emergency room visit) or hospitalization occurs.

• Ask the participant to contact the site staff or investigator immediately if he or she experiences any respiratory symptoms.

• Complete the source documents.

• The investigator or an authorized designee completes the CRFs.

### 8.12.3. Study procedure for Animal Sentinel Cohorts for TOMAVAC

#### 8.12.3.1. Screening: (0 to 14 Days Before Observation 1)

The procedures below have to be conducted stepwise; ensure that procedures listed prior to administering the vaccine are conducted prior to vaccination.

• Assign a single animal number using the IRT system.

• Obtain the animal’s demography (age and sex). The full data will be collected to critically evaluate the immune response and safety profile.

• Perform physical examination including vital signs (weight, height, and vital activity), evaluating any clinically significant abnormalities within the following body systems: general appearance; skin; head, eyes, ears, nose.

• Collect a blood sample (approximately 0.2 mL) for serological assessment of prior COVID-19 infection.

• Ensure and document that all of the inclusion criteria and none of the exclusion criteria are met.

• Complete the source documents.

• Complete the CRF.

#### 8.12.3.2. Observation 1 - Vaccination: (Day 1-3)

The procedures below have to be conducted stepwise; ensure that procedures listed prior to administering the vaccine are conducted prior to vaccination.

• Perform physical examination including vital signs (weight, height, and vital activity), evaluating any clinically significant abnormalities within the following body systems: general appearance; skin; head, eyes, ears, nose.

• Ensure and document that all of the inclusion criteria and none of the exclusion criteria are met.

• Ensure that the animal meets none of the temporary delay criteria.

• Obtain the animal’s randomization number and study intervention allocation using the IRT system. Either blinded site staff or unblinded site staff members may obtain this information.

• Unblinded site staff member(s) will administer one dose of study vaccine (1 ml homogenate equal to ≈0.77 µg S1 protein) by force-feeding with a syringe.

• Record any acute AEs.

• Complete the source documents.

#### 8.12.3.3. Observation 2 - 1-Week Follow-up Observation (Vaccination 1): (6 to 8 Days After Observation 1)

• Record AEs, including animal death.

• Perform physical examination including vital signs (weight, height, and vital activity), evaluating any clinically significant abnormalities within the following body systems: general appearance; skin; head, eyes, ears, nose.

• Complete the source documents.

• The investigator or an authorized designee completes the CRFs.

#### 8.12.3.4. Observation 3 - 2-Week Follow-up Observation 1 (Vaccination 1): (13 to 15 Days After Observation 1)

• Record AEs, including animal death.

• Perform physical examination including vital signs (weight, height, and vital activity), evaluating any clinically significant abnormalities within the following body systems: general appearance; skin; head, eyes, ears, nose.

• Carry out decapitation of 6 animals and collect a blood sample (approximately 1 mL) and intestinal lavage fluid (approximately 5.0 mL) for immunogenicity testing.

• Remaining animals are force-fed to inject a second dose of the vaccine (1 ml homogenate equal to ≈0.77 µg S1 protein) using a syringe.

• Complete the source documents.

• The investigator or an authorized designee completes the CRFs.

#### 8.12.3.5. Observation 4 - 3-Week Follow-up Observation (Vaccination 1): (20 to 22 Days After Observation 1)

• Record AEs, including animal death.

• Perform physical examination including vital signs (weight, height, and vital activity), evaluating any clinically significant abnormalities within the following body systems: general appearance; skin; head, eyes, ears, nose.

• Complete the source documents.

• The investigator or an authorized designee completes the CRFs.

#### 8.12.3.6. Observation 5 - 4-Week Follow-up Observation 1 (Vaccination 1): (27 to 29 Days After Observation 1)

• Record AEs, including animal death.

• Perform physical examination including vital signs (weight, height, and vital activity), evaluating any clinically significant abnormalities within the following body systems: general appearance; skin; head, eyes, ears, nose.

• Carry out decapitation of 6 animals and collect a blood sample (approximately 1 mL) and intestinal lavage fluid (approximately 5.0 mL) for immunogenicity testing.

• Complete the source documents.

• The investigator or an authorized designee completes the CRFs.

#### 8.12.3.7. Observation 6 - 5-Week Follow-up Observation (Vaccination 1): (34 to 36 Days After Observation 1)

• Record AEs, including animal death.

• Perform physical examination including vital signs (weight, height, and vital activity), evaluating any clinically significant abnormalities within the following body systems: general appearance; skin; head, eyes, ears, nose.

• Complete the source documents.

• The investigator or an authorized designee completes the CRFs.

#### 8.12.3.8. Observation 7 - 6-Week Follow-up Observation 1 (Vaccination 1): (41 to 43 Days After Observation 1)

• Record AEs, including animal death.

• Perform physical examination including vital signs (weight, height, and vital activity), evaluating any clinically significant abnormalities within the following body systems: general appearance; skin; head, eyes, ears, nose.

• Carry out decapitation of 6 animals and collect a blood sample (approximately 1 mL) and intestinal lavage fluid (approximately 5.0 mL) for immunogenicity testing.

• Complete the source documents.

• The investigator or an authorized designee completes the CRFs.

#### 8.12.3.9. Observation 8 - 7-Week Follow-up Observation (Vaccination 1): (48 to 50 Days After Observation 1)

• Record AEs, including animal death.

• Perform physical examination including vital signs (weight, height, and vital activity), evaluating any clinically significant abnormalities within the following body systems: general appearance; skin; head, eyes, ears, nose.

• Complete the source documents.

• The investigator or an authorized designee completes the CRFs.

#### 8.12.3.10. Observation 9 - 8-Week Follow-up Observation 1 (Vaccination 1): (55 to 57 Days After Observation 1)

• Record AEs, including animal death.

• Perform physical examination including vital signs (weight, height, and vital activity), evaluating any clinically significant abnormalities within the following body systems: general appearance; skin; head, eyes, ears, nose.

• Carry out decapitation of 6 animals and collect a blood sample (approximately 1 mL) and intestinal lavage fluid (approximately 5.0 mL) for immunogenicity testing.

• Complete the source documents.

• The investigator or an authorized designee completes the CRFs.

### 8.12.4. Study procedure for Animal Sentinel Cohorts for AstraZeneca

#### 8.12.4.1. Screening: (0 to 14 Days Before Observation 1)

The procedures below have to be conducted stepwise; ensure that procedures listed prior to administering the vaccine are conducted prior to vaccination.

• Assign a single animal number using the IRT system.

• Obtain the animal’s demography (age and sex). The full data will be collected to critically evaluate the immune response and safety profile.

• Perform physical examination including vital signs (weight, height, and vital activity), evaluating any clinically significant abnormalities within the following body systems: general appearance; skin; head, eyes, ears, nose.

• Collect a blood sample (approximately 0.2 mL) for serological assessment of prior COVID-19 infection.

• Ensure and document that all of the inclusion criteria and none of the exclusion criteria are met.

• Complete the source documents.

• Complete the CRF.

#### 8.12.4.2. Observation 1 - Vaccination: (Day 1)

The procedures below have to be conducted stepwise; ensure that procedures listed prior to administering the vaccine are conducted prior to vaccination.

• Perform physical examination including vital signs (weight, height, and vital activity), evaluating any clinically significant abnormalities within the following body systems: general appearance; skin; head, eyes, ears, nose.

• Ensure and document that all of the inclusion criteria and none of the exclusion criteria are met.

• Ensure that the animal meets none of the temporary delay criteria.

• Obtain the animal’s randomization number and study intervention allocation using the IRT system. Either blinded site staff or unblinded site staff members may obtain this information.

• Unblinded site staff member(s) will administer one dose of a positive control vaccine (75 µl of working solution from AstraZeneca vaccine equal to ≈16×10^6^ infectious units) by intramuscular injection.

• Record any acute AEs.

• Complete the source documents.

#### 8.12.4.3. Observation 2 - 1-Week Follow-up Observation (Vaccination 1): (6 to 8 Days After Observation 1)

• Record AEs, including animal death.

• Perform physical examination including vital signs (weight, height, and vital activity), evaluating any clinically significant abnormalities within the following body systems: general appearance; skin; head, eyes, ears, nose.

• Complete the source documents.

• The investigator or an authorized designee completes the CRFs.

#### 8.12.4.4. Observation 3 - 2-Week Follow-up Observation 1 (Vaccination 1): (14 Days After Observation 1)

• Record AEs, including animal death.

• Perform physical examination including vital signs (weight, height, and vital activity), evaluating any clinically significant abnormalities within the following body systems: general appearance; skin; head, eyes, ears, nose.

• Carry out decapitation of 6 animals and collect a blood sample (approximately 1 mL) and intestinal lavage fluid (approximately 5.0 mL) for immunogenicity testing.

• Remaining animals are force-fed to inject a second dose of a positive control vaccine (75 µl of working solution from AstraZeneca vaccine equal to ≈16×10^6^ infectious units) by intramuscular injection.

• Complete the source documents.

• The investigator or an authorized designee completes the CRFs.

#### 8.12.4.5. Observation 4 - 3-Week Follow-up Observation (Vaccination 1): (20 to 22 Days After Observation 1)

• Record AEs, including animal death.

• Perform physical examination including vital signs (weight, height, and vital activity), evaluating any clinically significant abnormalities within the following body systems: general appearance; skin; head, eyes, ears, nose.

• Complete the source documents.

• The investigator or an authorized designee completes the CRFs.

#### 8.12.4.6. Observation 5 - 4-Week Follow-up Observation 1 (Vaccination 1): (27 to 29 Days After Observation 1)

• Record AEs, including animal death.

• Perform physical examination including vital signs (weight, height, and vital activity), evaluating any clinically significant abnormalities within the following body systems: general appearance; skin; head, eyes, ears, nose.

• Carry out decapitation of 6 animals and collect a blood sample (approximately 1 mL) and intestinal lavage fluid (approximately 5.0 mL) for immunogenicity testing.

• Complete the source documents.

• The investigator or an authorized designee completes the CRFs.

#### 8.12.4.7. Observation 6 - 5-Week Follow-up Observation (Vaccination 1): (34 to 36 Days After Observation 1)

• Record AEs, including animal death.

• Perform physical examination including vital signs (weight, height, and vital activity), evaluating any clinically significant abnormalities within the following body systems: general appearance; skin; head, eyes, ears, nose.

• Complete the source documents.

• The investigator or an authorized designee completes the CRFs.

#### 8.12.4.8. Observation 7 - 6-Week Follow-up Observation 1 (Vaccination 1): (41 to 43 Days After Observation 1)

• Record AEs, including animal death.

• Perform physical examination including vital signs (weight, height, and vital activity), evaluating any clinically significant abnormalities within the following body systems: general appearance; skin; head, eyes, ears, nose.

• Carry out decapitation of 6 animals and collect a blood sample (approximately 1 mL) and intestinal lavage fluid (approximately 5.0 mL) for immunogenicity testing.

• Complete the source documents.

• The investigator or an authorized designee completes the CRFs.

#### 8.12.4.9. Observation 8 - 7-Week Follow-up Observation (Vaccination 1): (48 to 50 Days After Observation 1)

• Record AEs, including animal death.

• Perform physical examination including vital signs (weight, height, and vital activity), evaluating any clinically significant abnormalities within the following body systems: general appearance; skin; head, eyes, ears, nose.

• Complete the source documents.

• The investigator or an authorized designee completes the CRFs.

#### 8.12.4.10. Observation 9 - 8-Week Follow-up Observation 1 (Vaccination 1): (55 to 57 Days After Observation 1)

• Record AEs, including animal death.

• Perform physical examination including vital signs (weight, height, and vital activity), evaluating any clinically significant abnormalities within the following body systems: general appearance; skin; head, eyes, ears, nose.

• Carry out decapitation of 6 animals and collect a blood sample (approximately 1 mL) and intestinal lavage fluid (approximately 5.0 mL) for immunogenicity testing.

• Complete the source documents.

• The investigator or an authorized designee completes the CRFs.

### 8.12.5. Study Procedure for Animal Nonsentinel Cohorts

#### 8.12.5.1. Screening: (0 to 14 Days Before Observation 1)

The procedures below have to be conducted stepwise; ensure that procedures listed prior to administering the vaccine are conducted prior to vaccination.

• Assign a single animal number using the IRT system.

• Obtain the animal’s demography (age and sex). The full data will be collected to critically evaluate the immune response and safety profile.

• Perform physical examination including vital signs (weight, height, and vital activity), evaluating any clinically significant abnormalities within the following body systems: general appearance; skin; head, eyes, ears, nose.

• Collect a blood sample (approximately 0.2 mL) for serological assessment of prior COVID-19 infection.

• Ensure and document that all of the inclusion criteria and none of the exclusion criteria are met.

• Complete the source documents.

• Complete the CRF.

#### 8.12.5.2. Observation 1 - Vaccination: (Day 1-3)

The procedures below have to be conducted stepwise; ensure that procedures listed prior to administering the vaccine are conducted prior to vaccination.

• Perform physical examination including vital signs (weight, height, and vital activity), evaluating any clinically significant abnormalities within the following body systems: general appearance; skin; head, eyes, ears, nose.

• Ensure and document that all of the inclusion criteria and none of the exclusion criteria are met.

• Ensure that the animal meets none of the temporary delay criteria.

• Obtain the animal’s randomization number and study intervention allocation using the IRT system. Either blinded site staff or unblinded site staff members may obtain this information.

• Unblinded site staff member(s) will administer the first dose of control tomato (1 ml homogenate) by force-feeding with a syringe.

• Record any acute AEs.

• Complete the source documents.

#### 8.12.5.3. Observation 2 - 1-Week Follow-up Observation (Vaccination 1): (6 to 8 Days After Observation 1)

• Record AEs, including animal death.

• Perform physical examination including vital signs (weight, height, and vital activity), evaluating any clinically significant abnormalities within the following body systems: general appearance; skin; head, eyes, ears, nose.

• Complete the source documents.

• The investigator or an authorized designee completes the CRFs.

#### 8.12.5.4. Observation 3 - 2-Week Follow-up Observation 1 (Vaccination 1): (13 to 15 Days After Observation 1)

• Record AEs, including animal death.

• Perform physical examination including vital signs (weight, height, and vital activity), evaluating any clinically significant abnormalities within the following body systems: general appearance; skin; head, eyes, ears, nose.

• Carry out decapitation of 6 animals and collect a blood sample (approximately 1 mL) and intestinal lavage fluid (approximately 5.0 mL) for immunogenicity testing.

• Remaining animals are force-fed to inject a second dose of control tomato (1 ml homogenate) using a syringe.

• Complete the source documents.

• The investigator or an authorized designee completes the CRFs.

#### 8.12.5.5. Observation 4 - 3-Week Follow-up Observation (Vaccination 1): (20 to 22 Days After Observation 1)

• Record AEs, including animal death.

• Perform physical examination including vital signs (weight, height, and vital activity), evaluating any clinically significant abnormalities within the following body systems: general appearance; skin; head, eyes, ears, nose.

• Complete the source documents.

• The investigator or an authorized designee completes the CRFs.

#### 8.12.5.6. Observation 5 - 4-Week Follow-up Observation 1 (Vaccination 1): (27 to 29 Days After Observation 1)

• Record AEs, including animal death.

• Perform physical examination including vital signs (weight, height, and vital activity), evaluating any clinically significant abnormalities within the following body systems: general appearance; skin; head, eyes, ears, nose.

• Carry out decapitation of 6 animals and collect a blood sample (approximately 1 mL) and intestinal lavage fluid (approximately 5.0 mL) for immunogenicity testing.

• Complete the source documents.

• The investigator or an authorized designee completes the CRFs.

#### 8.12.5.7. Observation 6 - 5-Week Follow-up Observation (Vaccination 1): (34 to 36 Days After Observation 1)

• Record AEs, including animal death.

• Perform physical examination including vital signs (weight, height, and vital activity), evaluating any clinically significant abnormalities within the following body systems: general appearance; skin; head, eyes, ears, nose.

• Complete the source documents.

• The investigator or an authorized designee completes the CRFs.

#### 8.12.5.8. Observation 7 - 6-Week Follow-up Observation 1 (Vaccination 1): (41 to 43 Days After Observation 1)

• Record AEs, including animal death.

• Perform physical examination including vital signs (weight, height, and vital activity), evaluating any clinically significant abnormalities within the following body systems: general appearance; skin; head, eyes, ears, nose.

• Carry out decapitation of 6 animals and collect a blood sample (approximately 1 mL) and intestinal lavage fluid (approximately 5.0 mL) for immunogenicity testing.

• Complete the source documents.

• The investigator or an authorized designee completes the CRFs.

#### 8.12.5.9. Observation 8 - 7-Week Follow-up Observation (Vaccination 1): (48 to 50 Days After Observation 1)

• Record AEs, including animal death.

• Perform physical examination including vital signs (weight, height, and vital activity), evaluating any clinically significant abnormalities within the following body systems: general appearance; skin; head, eyes, ears, nose.

• Complete the source documents.

• The investigator or an authorized designee completes the CRFs.

#### 8.12.5.10. Observation 9 - 8-Week Follow-up Observation 1 (Vaccination 1): (55 to 57 Days After Observation 1)

• Record AEs, including animal death.

• Perform physical examination including vital signs (weight, height, and vital activity), evaluating any clinically significant abnormalities within the following body systems: general appearance; skin; head, eyes, ears, nose.

• Carry out decapitation of 6 animals and collect a blood sample (approximately 1 mL) and intestinal lavage fluid (approximately 5.0 mL) for immunogenicity testing.

• Complete the source documents.

• The investigator or an authorized designee completes the CRFs.

## 8.13. Unscheduled Visit for Suspected Grade 4 Reaction

Suppose a Grade 3 local reaction, systemic event, or fever is reported in the diary. In that case, telephone contact should occur to ascertain further details and determine whether a site visit is clinically indicated. If a suspected Grade 4 local reaction, systemic event, or fever is reported in the diary, a telephone contact or site visit should occur to confirm whether the event meets the criteria for Grade 4.

A site visit must be scheduled as soon as possible to assess the participant unless any of the following is true:

• The participant is unable to attend the unscheduled visit.

• The local reaction/systemic event is no longer present at the telephone contact time.

• The participant recorded an incorrect value in the e-diary (confirmation of diary data entry error).

• The PI or authorized designee determined it was not needed.

This telephone contact will be recorded in the participant’s source documentation and the CRF.

Suppose the participant is unable to attend the unscheduled visit, or the PI or authorized designee determines it is not needed. In that case, any ongoing local reactions/systemic events must be assessed at the next study visit.

During the unscheduled visit, the reactions should be assessed by the investigator or a medically qualified member of the study staff, as applicable to the investigator’s local practice, who will:

• Measure body temperature (°C).

• Measure minimum and maximum diameters of redness (if present).

• Measure minimum and maximum diameters of swelling (if present).

• Assess systemic events (if present).

• Assess for other findings associated with the reaction and record them on the AE page of the CRF, if appropriate.

The investigator or an authorized designee will complete the unscheduled visit assessment page of the CRF.

## 8.14. COVID-19 Disease Surveillance (All Participants)

Suppose a participant experiences any of the following. In that case, he or she is instructed to contact the site immediately and, if confirmed, participate in a visit as soon as possible, optimally within three days of symptom onset. Note that this does not substitute for a participant’s routine medical care. Therefore, participants should be encouraged to seek care from their usual provider if appropriate.

• A diagnosis of COVID-19;

• Fever;

• New or increased cough;

• New or increased shortness of breath;

• New or increased sore throat;

• New or increased wheezing;

• New or increased sputum production;

• New or increased nasal congestion;

• New or increased nasal discharge;

• Loss of taste/smell.

### 8.14.1. Potential COVID-19 Illness Visit: (Optimally Within 3 Days After Potential COVID-19 Illness Onset)

This visit is expected to involve sharing healthcare information and services via telecommunication technologies remotely, thus allowing the participant and investigator to communicate on aspects of clinical care.

As a participant’s COVID-19 illness may evolve, several contacts may be required to obtain the following information:

• Record AEs.

• Record details of any prohibited medications received by the participant if required for his or her clinical care.

• Instruct the participant to self-collect a nasal (mid-turbinate) swab and ship it for assessment at the laboratory. The result from this swab will be provided to the site once it is available, but it will not be in real-time, and it cannot be relied upon to direct clinical care. Therefore, the participant should be encouraged to seek care, if appropriate, from his or her usual provider.

• Collect COVID-19-related standard-of-care clinical and laboratory information. This includes, but is not limited to:

• Symptoms

• Clinical diagnosis

• Local laboratory COVID-19 test result

• Full blood count

• C-reactive protein

• Number and type of any healthcare contacts; duration of hospitalization and intensive care unit stay

• Need for oxygen therapy

• Need for lung ventilation

• Schedule an appointment for the participant to return for the potential COVID-19 convalescent visit once he or she has recovered.

• Complete the source documents.

• The investigator or an authorized designee completes the CRFs.

### 8.13.2. Potential COVID-19 Convalescent Visit: (28 to 35 Days After Potential COVID-19 Illness Visit)

• Record AEs, as appropriate.

• Record details of any prohibited medications received by the participant if required for his or her clinical care.

• Collect a blood sample (approximately 5 mL) for immunogenicity testing.

• Collect/update COVID-19-related clinical and laboratory information.

• Complete the source documents.

• The investigator or an authorized designee completes the CRFs.

# 9. SUPPORTING DOCUMENTATION AND OPERATIONAL CONSIDERATIONS

## 9.1. Appendix A: Regulatory, Ethical, and Study Oversight Considerations

### 9.1.1. Regulatory and Ethical Considerations

This study will be conducted in accordance with the protocol and with the following:

• Consensus ethical principles derived from international guidelines, including the Declaration of Helsinki and CIOMS International Ethical Guidelines;

• Applicable ICH GCP guidelines;

• Applicable laws and regulations, including applicable privacy laws.

The protocol, protocol amendments, ICD, SRSD(s), and other relevant documents (e.g., advertisements) must be reviewed and approved by the Center and submitted to an IRB/EC by the investigator and reviewed and approved by the IRB/EC before the study is initiated.

Any amendments to the protocol will require IRB/EC approval before implementation of changes made to the study design, except for changes necessary to eliminate an immediate hazard to study participants.

The investigator will be responsible for the following:

• Providing written summaries of the status of the study to the IRB/EC annually or more frequently in accordance with the requirements, policies, and procedures established by the IRB/EC;

• Notifying the IRB/EC of SAEs or other significant safety findings as required by IRB/EC

procedures;

• Providing oversight of the conduct of the study at the site and adherence to requirements of 21 CFR, ICH guidelines, the IRB/EC, European regulation 536/2014 for clinical studies (if applicable), and all other applicable local regulations.

#### 9.1.1.1. Reporting of Safety Issues and Serious Breaches of the Protocol or ICH GCP

In the event of any prohibition or restriction imposed (i.e., clinical hold) by an applicable regulatory authority in any area of the world or if the investigator is aware of any new information that might influence the evaluation of the benefits and risks of the study intervention, Center of Genomics and Bioinformatics of Academy Sciences of Uzbekistan should be informed immediately.

In addition, the investigator will inform the Center of Genomics and Bioinformatics of the Academy of Sciences of Uzbekistan immediately of any urgent safety measures taken by the investigator to protect the study participants against any immediate hazard and of any serious breaches of this protocol or ICH GCP that the investigator becomes aware of.

### 9.1.2. Informed Consent Process

The investigator or his/her representative will explain the nature of the study to the participant and answer all questions regarding the study. The participant should be given sufficient time and opportunity to ask questions and decide whether to participate in the trial.

Participants must be informed that their participation is voluntary. Participants must sign a statement of informed consent that meets the requirements of 21 CFR 50, local regulations, ICH guidelines, HIPAA requirements, where applicable, and the IRB/EC or study center.

The investigator must ensure that each study participant is fully informed about the nature and objectives of the study, the sharing of data related to the study, and possible risks associated with participation, including the risks associated with the processing of the participant’s personal data.

The participant must be informed that his/her personal study-related data will be used by the Center in accordance with local data protection law. The level of disclosure must also be explained to the participant.

The participant must be informed that his/her medical records may be examined by Clinical Quality Assurance auditors or other authorized personnel appointed by the Center, appropriate IRB/EC members, and inspectors from regulatory authorities.

The investigator further must ensure that each study participant is fully informed about his or her right to access and correct his or her data and to withdraw consent for processing his or her personal data.

The medical record must include a statement that written informed consent was obtained before the participant was enrolled in the study and the date the written consent was obtained. The authorized person obtaining the informed consent must also sign the ICD.

Participants must be reconsented to the most current version of the ICD(s) during their participation in the study.

A copy of the ICD(s) must be provided to the participant. Participants who are rescreened are required to sign a new ICD.

Unless prohibited by local requirements or IRB/EC decision, the ICD will contain a separate section that addresses the use of samples for optional additional research. The optional additional research does not require the collection of any further samples. The investigator or authorized designee will explain to each participant the objectives of the additional study. Participants will be told that they can refuse to participate and may withdraw their consent at any time and for any reason during the storage period.

### 9.1.3. Data Protection

All parties will comply with all applicable laws, including laws regarding the implementation of organizational and technical measures to ensure the protection of participant data.

Participants’ personal data will be stored at the study site in encrypted electronic and/or paper form and will be password protected or secured in a locked room to ensure that only authorized study staff have access. The study site will implement appropriate technical and organizational measures to ensure that personal data can be recovered in the event of a disaster. In the event of a potential personal data breach, the study site will be responsible for determining whether a personal data breach has, in fact, occurred and, if so, providing breach notifications as required by law.

To protect the rights and freedoms of participants with regard to the processing of personal data, participants will be assigned a single, participant-specific numerical code. Any participant records or data sets that are transferred to the Center will contain the numerical code; participant names will not be transferred. This single, participant-specific code will identify all other identifiable data transferred to the Center. The study site will maintain a confidential list of participants who participated in the study, linking each participant’s numerical code to his or her actual identity and medical record identification. In case of data transfer, the Center will protect the confidentiality of participants’ data consistent with the clinical study agreement and applicable privacy laws.

### 9.1.4. Data Quality Assurance

All participant data relating to the study will be recorded on printed or electronic CRF. The investigator is responsible for verifying that data entries are accurate and correct by physically or electronically signing the CRF.

The investigator must maintain accurate documentation (source data) that supports the information entered in the CRF.

The investigator must ensure that the CRFs are securely stored at the study site in encrypted electronic and/or paper form and are password protected or secured in a locked room to prevent access by unauthorized third parties.

The investigator must permit study-related monitoring, audits, IRB/EC review, and regulatory agency inspections and provide direct access to source data documents. This verification may also occur after study completion. It is essential that the investigator(s) and their relevant personnel are available during the monitoring visits and possible audits or inspections and that sufficient time is devoted to the process.

Monitoring details describing strategy (e.g., risk-based initiatives in operations and quality such as risk management and mitigation strategies and analytical risk-based monitoring), methods, responsibilities, and requirements, including handling of noncompliance issues and monitoring techniques (central, remote, or on-site monitoring), are provided in the monitoring plan.

The Center or designee is responsible for the data management of this study, including quality checking of the data.

Study monitors will perform ongoing source data verification to confirm that data entered into the CRF by authorized site personnel are accurate, complete, and verifiable from source documents; that the safety and rights of participants are being protected; and that the study is being conducted in accordance with the currently approved protocol and any other study agreements, ICH GCP, and all applicable regulatory requirements.

Records and documents, including signed ICDs, pertaining to the conduct of this study must be retained by the investigator for 15 years after study completion unless local regulations or institutional policies require a longer retention period. No records may be destroyed during the retention period without the written approval of the Center. No records may be transferred to another location or party without written notification to the Center. The investigator must ensure that the records continue to be stored securely for as long as they are maintained.

When participant data are to be deleted, the investigator will ensure that all copies of such data are promptly and irrevocably deleted from all systems.

The investigator(s) will notify the Center or its agents immediately of any regulatory inspection notification in relation to the study. Furthermore, the investigator will cooperate with the Center or its agents to prepare the investigator site for the inspection and will allow the Center or its agent, whenever feasible, to be present during the inspection. The investigator site and investigator will promptly resolve any discrepancies that are identified between the study data and the participant's medical records. The investigator will promptly provide copies of the inspection findings to the Center or its agent. Before response submission to the regulatory authorities, the investigator will provide the Center or its agents with an opportunity to review and comment on responses to any such findings.

### 9.1.5. Source Documents

Source documents provide evidence for the existence of the participant and substantiate the integrity of the data collected. Source documents are filed at the investigator site.

Data reported on the CRF or entered in the eCRF that are from source documents must be consistent with the source documents, or the discrepancies must be explained. The investigator may need to request previous medical or transfer records, depending on the study. Also, current medical records must be available.

A definition of what constitutes source data can be found in the study monitoring plan.

A description of the use of a computerized system is documented in the Data Management Plan.

### 9.1.6. Study and Site Start and Closure

The study start date is the date on which the clinical study will be open for the recruitment of participants.

The first act of recruitment is the date of the first participant’s first visit and will be the study start date.

The Center designee reserves the right to close the study site or terminate the study at any time for any reason at the sole discretion of the Center. Study sites will be closed upon study completion. A study site is considered closed when all required documents and study supplies have been collected, and a study-site closure visit has been performed.

The investigator may initiate study-site closure at any time upon notification to the Center or designee if requested to do so by the responsible IRB/EC or if such termination is required to protect the health of study participants.

Reasons for the early closure of a study site by the Center may include but are not limited to:

• Failure of the investigator to comply with the protocol, the requirements of the IRB/EC or local health authorities, the Center's procedures, or GCP guidelines;

• Inadequate recruitment of participants by the investigator;

• Discontinuation of further study intervention development.

Suppose the study is prematurely terminated or suspended. In that case, the Center shall promptly inform the investigators, the ECs/IRBs, the regulatory authorities, and any CRO(s) used in the study of the reason for termination or suspension, as specified by the applicable regulatory requirements. The investigator shall promptly inform the participant and ensure appropriate participant therapy and/or follow-up.

Study termination is also provided for in the clinical study agreement. If there is any conflict between the contract and this protocol, the contract will control termination rights.

### 9.1.8. Center’s Qualified Medical Personnel

The contact information for the Center's appropriately qualified medical personnel for the study is documented in the study contact list located in the supporting study documentation.

To facilitate access to appropriately qualified medical personnel on study-related medical questions or problems, participants are provided with a contact card at the time of informed consent. The contact card contains, at a minimum, protocol and study intervention identifiers, participant numbers, contact information for the investigator site, and contact details for a contact center if the investigator site staff cannot be reached to provide advice on a medical question or problem originating from another healthcare professional not involved in the participant’s participation in the study. The contact number can also be used by investigator staff if they seek advice on medical questions or problems; however, it should be used only if the established communication pathways between the investigator site and the study team are unavailable. It is therefore intended to augment, but not replace, the established communication pathways between the investigator site and the study team for advice on medical questions or problems that may arise during the study. The contact number is not intended for use by the participant directly, and if a participant calls that number, he or she will be directed back to the investigator’s site.

**9.2. Appendix B: Adverse Events: Definitions and Procedures for Recording, Evaluating, Follow-up, and Reporting**

### 9.2.1. Definition of AE

**AE Definition**

• An AE is any untoward medical occurrence in a patient or clinical study participant temporally associated with the use of study intervention, whether or not considered related to the study intervention.

• NOTE: An AE can therefore be any unfavorable and unintended sign (including an abnormal laboratory finding), symptom, or disease (new or exacerbated) temporally associated with the use of study intervention.

**Events Meeting the AE Definition**

• Any abnormal laboratory test results (hematology, clinical chemistry, or urinalysis) or other safety assessments (e.g., ECG, radiological scans, vital sign measurements), including those that worsen from baseline, considered clinically significant in the medical and scientific judgment of the investigator Any abnormal laboratory test results that meet any of the conditions below must be recorded as an AE:

• Is associated with accompanying symptoms.

• Requires additional diagnostic testing or medical/surgical intervention.

• Leads to a change in study dosing (outside of any protocol-specified dose adjustments) or discontinuation from the study, significant additional concomitant drug treatment, or other therapy.

• Exacerbation of a chronic or intermittent pre-existing condition, including either an increase in frequency and/or intensity of the condition.

• New conditions detected or diagnosed after study intervention administration, even though they may have been present before the start of the study.

• Signs, symptoms, or the clinical sequelae of a suspected drug-drug interaction.

• Signs, symptoms, or the clinical sequelae of a suspected overdose of either study intervention or concomitant medication. Overdose per se will not be reported as an AE/SAE unless it is an intentional overdose taken with possible suicidal/self-harming intent. Such overdoses should be reported regardless of sequelae.

| **Events NOT Meeting the AE Definition** | |
| --- | --- |
| • | Any clinically significant abnormal laboratory findings or other abnormal safety assessments which are associated with the underlying disease, unless judged by the investigator to be more severe than expected for the participant’s condition. |
| • | The disease/disorder being studied or expected progression, signs, or symptoms of the disease/disorder being studied, unless more severe than expected for the participant’s condition. |
| • | Medical or surgical procedure (e.g., endoscopy, appendectomy): the condition that leads to the procedure is the AE. |
| • | Situations where an untoward medical occurrence did not occur (social and/or convenience admission to a hospital). |
| • | Anticipated day-to-day fluctuations of pre-existing disease(s) or condition(s) present or detected at the start of the study that does not worsen. |

### 9.2.2. Definition of SAE

Suppose an event is not an AE per the definition above. In that case, it cannot be an SAE even if severe conditions are met (e.g., hospitalization for signs/symptoms of the disease under study, death due to the progression of the disease).

| **An SAE is defined as any untoward medical occurrence that, at any dose:** |
| --- |
| **a. Results in death** |
| **b. Is life-threatening**  The term “life-threatening” in the definition of “serious” refers to an event in which the participant was at risk of death at the time of the event. It does not refer to an event that hypothetically might have caused death if it were more severe. |
| **c. Requires inpatient hospitalization or prolongation of existing hospitalization**  Hospitalization generally signifies that the participant has been detained (usually involving at least an overnight stay) at the hospital or emergency ward for observation and/or treatment that would not have been appropriate in the physician’s office or outpatient setting.  Complications that occur during hospitalization are AEs. If a complication prolongs hospitalization or fulfils any other severe criteria, the event is serious. When in doubt as to whether “hospitalization” occurred or was necessary, the AE should be considered severe.  Hospitalization for elective treatment of a pre-existing condition that did not worsen from baseline is not considered an AE. |
| **d. Results in persistent disability/incapacity**  • The term disability means a substantial disruption of a person’s ability to conduct normal life functions.  • This definition is not intended to include experiences of relatively minor medical significance such as uncomplicated headache, nausea, vomiting, diarrhea, influenza, and accidental trauma (e.g., sprained ankle), which may interfere with or prevent everyday life functions but do not constitute a substantial disruption. |
| **e. Is a congenital anomaly/birth defect** |
| **f. Other situations:**  • Medical or scientific judgment should be exercised in deciding whether SAE reporting is appropriate in other situations, such as critical medical events that may not be immediately life-threatening or result in death or hospitalization but may jeopardize the participant or may require medical or surgical intervention to prevent one of the other outcomes listed in the above definition. These events should usually be considered severe.  • Examples of such events include invasive or malignant cancers, intensive treatment in an emergency room or at home for allergic bronchospasm, blood dyscrasias or convulsions that do not result in hospitalization, development of drug dependency or drug abuse. |

### 9.2.3. Recording/Reporting and Follow-up of AEs and/or SAEs

**AE and SAE Recording/Reporting**

The table below summarises the requirements for recording adverse events on the CRF and reporting serious adverse events on the Vaccines SAE Report Form to the Center of Genomics and Bioinformatics. These requirements are delineated for three types of events: (1) SAEs; (2) nonserious adverse events (AEs); and (3) exposure to the study intervention under study during pregnancy or breastfeeding and occupational exposure.

It should be noted that the Vaccines SAE Report Form for reporting SAE information is not the same as the AE page of the CRF. When the same data are collected, the forms must be completed in a consistent manner. AEs should be recorded using concise medical terminology, and the same AE term should be used on both the CRF and the Vaccines SAE Report Form for reporting SAE information.

| **Safety Event** | **Recorded on the CRF** | **Reported on the Vaccines SAE Report Form Within 24 Hours of Awareness** |
| --- | --- | --- |
| SAE | All | All |
| Nonserious AE | All | None |
| Exposure to the study intervention under study during pregnancy or breastfeeding and occupational exposure | **None** | All (and EDP supplemental form for EDP) |

• When an AE/SAE occurs, it is the responsibility of the investigator to review all documentation (e.g., hospital progress notes, laboratory reports, and diagnostic reports) related to the event.

• The investigator will then record all relevant AE/SAE information in the CRF.

• It is not acceptable for the investigator to send photocopies of the participant’s medical records to the Center of Genomics and Bioinformatics of the Academy Sciences of Uzbekistan instead of completing the Vaccines SAE Report Form/AE/SAE CRF page.

• There may be instances when the Center of Genomics and Bioinformatics of the Academy of Sciences of Uzbekistan requests copies of medical records for certain cases. In this case, all participant identifiers, with the exception of the participant number, will be redacted on the copies of the medical records before submission to the Center of Genomics and Bioinformatics of the Academy of Sciences of Uzbekistan.

• The investigator will attempt to establish a diagnosis of the event based on signs, symptoms, and/or other clinical information. Whenever possible, the diagnosis (not the individual signs/symptoms) will be documented as the AE/SAE.

**Assessment of Intensity**

The investigator will make an assessment of intensity for each AE and SAE reported during the study and assign it to 1 of the following categories:

| GRADE | If required on the AE page of the CRF, the investigator will use the adjectives MILD, MODERATE, SEVERE, or LIFE-THREATENING to describe the maximum intensity of the AE. For purposes of consistency, these intensity grades are defined as follows: | |
| --- | --- | --- |
| 1 | MILD | Does not interfere with the participant's usual function. |
| 2 | MODERATE | Interferes to some extent with the participant's usual function. |
| 3 | SEVERE | Interferes significantly with the participant's usual function. |
| 4 | LIFE-THREATENING | Life-threatening consequences; urgent intervention indicated. |

**Assessment of Causality**

• The investigator must assess the relationship between study intervention and each occurrence of each AE/SAE.

• A “reasonable possibility” of a relationship conveys that there are facts, evidence, and/or arguments to suggest a causal relationship rather than a relationship that cannot be ruled out.

• The investigator will use clinical judgment to determine the relationship.

• Alternative causes, such as underlying disease(s), concomitant therapy, and other risk factors, as well as the temporal relationship of the event to study intervention administration, will be considered and investigated.

• The investigator will also consult the IB and/or product information, for marketed products, in his/her assessment.

• For each AE/SAE, the investigator must document in the medical notes that he/she has reviewed the AE/SAE and has provided a causality assessment.

• There may be situations where an SAE has occurred, and the investigator has minimal information to include in the initial report to the Center. However, the investigator always makes an assessment of causality for every event before the initial transmission of the SAE data to the Center.

• The investigator may change his/her opinion of causality in light of follow-up information and send an SAE follow-up report with the updated causality assessment.

• The causality assessment is one of the criteria used when determining regulatory reporting requirements.

• If the investigator does not know whether or not the study intervention caused the event, then the event will be handled as “related to study intervention” for reporting purposes, as defined by the Center. In addition, if the investigator determines that an SAE is associated with study procedures, the investigator must record this causal relationship in the source documents and CRF and report such an assessment in the dedicated section of the Vaccines SAE Report Form and in accordance with the SAE reporting requirements.

**Follow-up of AEs and SAEs**

• The investigator is obligated to perform or arrange for the conduct of supplemental measurements and/or evaluations as medically indicated or as requested by the Center to elucidate the nature and/or causality of the AE or SAE as fully as possible. This may include additional laboratory tests, investigations, histopathological examinations, or consultation with other healthcare providers.

• If a participant dies during participation in the study or during a recognized follow-up period, the investigator will provide the Center of Genomics and Bioinformatics with a copy of any post-mortem findings, including histopathology.

• New or updated information will be recorded in the originally completed CRF.

• The investigator will submit any updated SAE data to the Center within 24 hours of receipt of the information.

### 9.2.4. Reporting of SAEs

SAE Reporting to Center of Genomics and Bioinformatics of Academy Sciences of Uzbekistan via Vaccines SAE Report Form

• Facsimile transmission of the Vaccines SAE Report Form is the preferred method to transmit this information to the Center of Genomics and Bioinformatics.

• In circumstances when the facsimile is not working, notification by telephone is acceptable with a copy of the Vaccines SAE Report Form sent by overnight mail or courier service.

• Initial notification via telephone does not replace the need for the investigator to complete and sign the Vaccines SAE Report Form pages within the designated reporting time frames.

### 9.3. Appendix C: Abbreviations

The following is a list of abbreviations that may be used in the protocol.

| **Abbreviation** | **Term** |
| --- | --- |
| 2019-nCoV | novel coronavirus 2019 |
| AE | adverse event |
| BMI | body mass index |
| CBER | Center for Biologics Evaluation and Research |
| CFR | Code of Federal Regulations |
| CI | confidence interval |
| CIOMS | Council for International Organizations of Medical Sciences |
| CONSORT | Consolidated Standards of Reporting Trials |
| COVID-19 | coronavirus disease 2019 |
| CRF | case report form |
| CRO | contract research organization |
| CSR | clinical study report |
| CT | clinical trial |
| DMC | data monitoring committee |
| DNA | deoxyribonucleic acid |
| DU | dosing unit |
| EC | ethics committee |
| eCRF | electronic case report form |
| EMA | European Medicines Agency |
| EU | European Union |
| EUA | emergency use application |
| EudraCT | European Clinical Trials Database |
| FDA | Food and Drug Administration |
| GCP | Good Clinical Practice |
| GMC | geometric mean concentration |
| GMFR | geometric mean fold rise |
| GMR | geometric mean ratio |
| GMT | geometric mean titer |
| HBc Ab | hepatitis B core antibody |
| HBsAg | hepatitis B surface antigen |
| HBV | hepatitis B virus |
| HCV | hepatitis C virus |
| HCV Ab | hepatitis C virus antibody |
| HIPAA | Health Insurance Portability and Accountability Act |
| HIV | human immunodeficiency virus |
| IB | investigator’s brochure |
| ICD | informed consent document |
| ICH | International Council for Harmonisation |
| ICU | intensive care unit |
| ID | identification |
| Ig | immunoglobulin |
| IgG | immunoglobulin G |
| IgM | immunoglobulin M |
| IMP | investigational medicinal product |
| IND | investigational new drug |
| INR | international normalized ratio |
| IP manual | investigational product manual |
| IPAL | Investigational Product Accountability Log |
| IRB | institutional review board |
| IRC | internal review committee |
| IRR | infection rate ratio |
| IRT | interactive response technology |
| IWR | interactive Web-based response |
| LLOQ | The lower limit of quantitation |
| MedDRA | Medical Dictionary for Regulatory Activities |
| MERS | Middle East respiratory syndrome |
| N/A | not applicable |
| NAAT | nucleic acid amplification test |
| NVA | nonvaccine antigen |
| PCR | polymerase chain reaction |
| PI | principal investigator |
| PPE | personal protective equipment |
| RBD | receptor-binding domain |
| RCDC | The reverse cumulative distribution curve |
| RNA | ribonucleic acid |
| RSV | respiratory syncytial virus |
| RT-PCR | reverse transcription-polymerase chain reaction |
| SAE | serious adverse event |
| saRNA | self-amplifying messenger ribonucleic acid |
| SARS | severe acute respiratory syndrome |
| SARS-CoV-2 | severe acute respiratory syndrome coronavirus 2 |
| S-IgA | secretory immunoglobulin A |
| SoA | schedule of activities |
| SOP | standard operating procedure |
| SRSD | the single reference safety document |
| SUSAR | suspected unexpected serious adverse reaction |
| TBD | to be determined |
| US | United States |
| vac | vaccination |
| VE | vaccine efficacy |
| WHO | World Health Organization |

**References**

1. COVID-19 Dashboard by the Center for Systems Science and Engineering (CSSE) at Johns Hopkins University (JHU). https://www.arcgis.com/apps/dashboards/bda7594740fd40299423467b48e9ecf6 [Accessed December 22, 2022].
2. Maharjan PM, Choe S. Plant-Based COVID-19 Vaccines: Current Status, Design, and Development Strategies  of Candidate Vaccines. *Vaccines* (2021) 9: doi: 10.3390/vaccines9090992
3. Hadj Hassine I. Covid-19 vaccines and variants of concern: A review. *Rev Med Virol* (2022) 32:e2313. doi: 10.1002/rmv.2313
4. Tregoning JS, Flight KE, Higham SL, Wang Z, Pierce BF. Progress of the COVID-19 vaccine effort: viruses, vaccines and variants versus  efficacy, effectiveness and escape. *Nat Rev Immunol* (2021) 21:626–636. doi: 10.1038/s41577-021-00592-1
5. Sohrab SS. An edible vaccine development for coronavirus disease 2019: the concept. *Clin Exp Vaccine Res* (2020) 9:164–168. doi: 10.7774/cevr.2020.9.2.164
6. Status of COVID-19 Vaccines within WHO EUL/PQ evaluation process. https://extranet.who.int/pqweb/sites/default/files/documents/Status_COVID_VAX_08November2022.pdf [Accessed November 8, 2022]
7. COVID-19 vaccine tracker and landscape. www.who.int/publications/m/item/draft-landscape-of-covid-19-candidate-vaccines [Accessed November 8, 2022]
8. Nochi T, Takagi H, Yuki Y, Yang L, Masumura T, Mejima M, Nakanishi U, Matsumura A, Uozumi A, Hiroi T, et al. Rice-based mucosal vaccine as a global strategy for cold-chain- and needle-free  vaccination. *Proc Natl Acad Sci U S A* (2007) 104:10986–10991. doi: 10.1073/pnas.0703766104
9. Azegami T, Itoh H, Kiyono H, Yuki Y. Novel transgenic rice-based vaccines. *Arch Immunol Ther Exp (Warsz)* (2015) 63:87–99. doi: 10.1007/s00005-014-0303-0
10. Christensen D, Polacek C, Sheward DJ, Hanke L, Moliner-Morro A, McInerney G, Murrell B, Hartmann KT, Jensen HE, Jungersen G, et al. Protection against SARS-CoV-2 transmission by a parenteral prime-Intranasal boost  vaccine strategy. *EBioMedicine* (2022) 84:104248. doi: 10.1016/j.ebiom.2022.104248
11. Miteva D, Peshevska-Sekulovska M, Snegarova V, Batselova H, Alexandrova R, Velikova T. Mucosal COVID-19 vaccines: Risks, benefits and control of the pandemic. World J Virol (2022) 11:221–236. doi: 10.5501/wjv.v11.i5.221
12. Kurup VM, Thomas J. Edible Vaccines: Promises and Challenges. *Mol Biotechnol* (2020) 62:79–90. doi: 10.1007/s12033-019-00222-1
13. Vela Ramirez JE, Sharpe LA, Peppas NA. Current state and challenges in developing oral vaccines. *Adv Drug Deliv Rev* (2017) 114:116–131. doi: 10.1016/j.addr.2017.04.008
14. Concha C, Cañas R, Macuer J, Torres MJ, Herrada AA, Jamett F, Ibáñez C. Disease Prevention: An Opportunity to Expand Edible Plant-Based Vaccines? *Vaccines* (2017) 5: doi: 10.3390/vaccines5020014
15. Food and Drug Administration. *Toxicity Grading Scale for Healthy Adult and Adolescent Volunteers Enrolled in Preventive Vaccine Clinical Trials*. Rockville: Centre for Biologics Evaluation and Research (2007). https://www.fda.gov/regulatory-information/search-fda-guidance-documents/toxicity-grading-scale-healthy-adult-and-adolescent-volunteers-enrolled-preventive-vaccine-clinical
